# Synergy of Experiment and Broadened Exploration of Ab Initio Calculations for Understanding of Lanthanide–Pentacyanidocobaltate Molecular Nanomagnets and Their Optical Properties

**DOI:** 10.1021/acs.inorgchem.4c02793

**Published:** 2024-09-02

**Authors:** Mikolaj Zychowicz, Hubert Dzielak, Jan Rzepiela, Szymon Chorazy

**Affiliations:** † Faculty of Chemistry, Jagiellonian University, Gronostajowa 2, 30-387 Krakow, Poland; ‡ Jagiellonian University, Doctoral School of Exact and Natural Sciences, Lojasiewicza 11, 30-348 Krakow, Poland

## Abstract

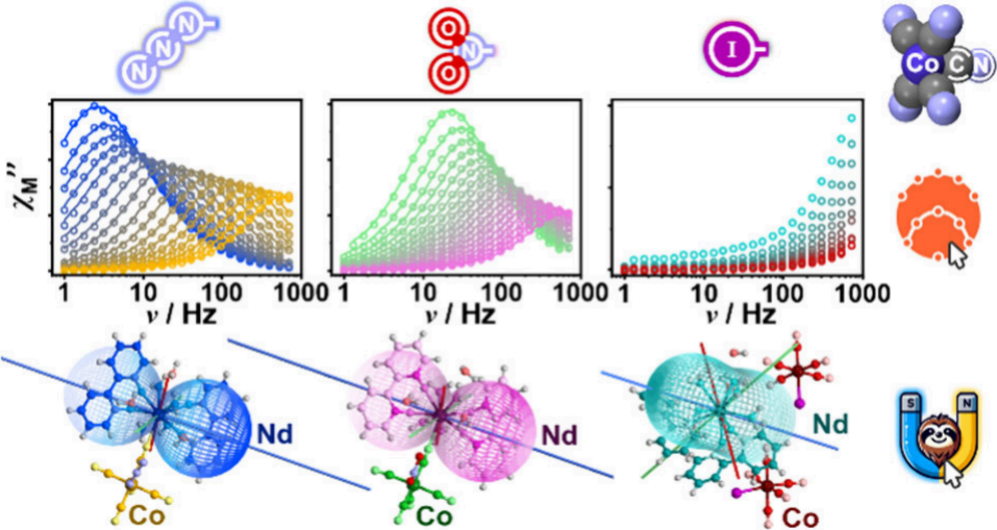

We present a synergistic experimental–theoretical methodology for the investigation of lanthanide-based single-molecule magnets (SMMs), demonstrated using the example of novel heterometallic molecules incorporating Nd^3+^/Ce^3+^ ions combined with three different, rarely explored, pentacyanidocobaltate(III) metalloligands, [Co^III^(CN)_5_(azido/nitrito-*N*/iodido)]^3–^. The theoretical part of our approach broadens the exploration of *ab initio* calculations for lanthanide(III) complexes toward the convenient simulations of such physical characteristics as directional dependences of Helmholtz energy, magnetization, susceptibility, and their thermal and field evolution, as well as light absorption and emission bands. This work was conducted using newly designed SlothPy software (https://slothpy.org). It is introduced as an open-source Python library for simulating various physical properties from first-principles based on results of electronic structure calculations obtained within popular quantum chemistry packages. The computational results were confronted with spectroscopic and *ac*/*dc*-magnetic data, the latter analyzed using previously designed relACs software. The combination of experimental and computational methods gave insight into phonon-assisted magnetic relaxation mechanisms, disentangling them from the temperature-independent quantum tunneling of magnetization and emphasizing the role of local-mode processes. This study provides an understanding of the changes in lanthanide(III) magnetic anisotropy introduced with pentacyanidocobaltates(III) modifications, theoretically exploring also potential applications of reported compounds as anisotropy switches or optical thermometers.

## Abstract 2

A novel family of heterometallic molecular materials composed of lanthanide(III) complexes and pentacyanidocobaltate(III) metalloligands, the latter bearing three different inorganic anions, was synthesized. They exhibit lanthanide-centered slow relaxation of magnetization, which was deeply investigated using synergistic experimental−theoretical methodology. It includes the broadened exploration of *ab initio* calculations using newly designed software, SlothPy, which enables the simulations of diverse physical characteristics, giving insight into complex magnetic relaxation processes and optical properties of obtained metal complexes.

## Introduction

Since the discovery of magnetic hysteresis in Mn_12_-acetate,^1^ single-molecule magnets (SMMs) have dramatically revolutionized the domain of molecular magnetism.^2,3^ Researchers, motivated by the prospect of unprecedented miniaturization in data storage^4^ along with potential applications in quantum computing^5^ and spintronics^6,7^ that SMMs offer, swiftly recognized lanthanide (Ln) ions as promising building blocks for nanomagnets.^8^ Owing to strong spin–orbit coupling, shielded valence electrons, and the vast potential for modulating the ligand field, lanthanides emerged as ideal constituents for crafting SMMs far exceeding those using d-block metal ions.^9,10^ These efforts, underpinned by the development of experimental^11^ and theoretical techniques,^12^ culminated in the principles for engineering crystal fields of suitable geometries enhancing magnetic anisotropy, giving the strongly split, highly energetic, and axial electronic energy levels.^13^ The crystal field control is usually accomplished by organic ligands equipped with electron-rich donor groups and, often, bulky frames^14^ that can induce low coordination numbers and appropriate coordination geometry,^15^ stabilizing ground states of the largest magnetic momenta.^16^ The electronic structure designed in such a way also suppresses quantum tunneling of magnetization (QTM) and enforces relaxation via the thermally activated Orbach or thermally activated QTM (TA-QTM) processes, traversing the energy barrier.^17^ Regrettably, the dominant outlook on the static crystal field effects encountered limitations despite setting an initial guideline for high-performance SMMs.^18^ It happens due to phonon-assisted Raman processes facilitating relaxations beneath the energy barrier, which are now relatively easy to estimate using *ab initio* calculations.^19^ On the contrary, the higher-order (Raman) processes are inherently challenging to predict due to their dependence on lattice dynamics and spin–phonon interactions, whose strength depends on normal modes and periodic structure.^20,21^ Consequently, significant focus is placed on simulations of phonon-assisted relaxation rates from first-principles, aiming to take control over Raman relaxations.^22−24^

Besides the common usage of organic ligands to control the crystal field of Ln-SMMs,^25−30^ it was shown that metalloligands, such as polycyanidometallates, can be an alternative, also providing a route to enhance the Ln-luminescence.^25,31−35^ Moreover, they offer chemical modifiability to meet the dual objectives of anisotropy generation (static) and relaxation rate adjustment (dynamic) by the modulation of the crystal lattice and its phonons across a broad frequency range over the whole Brillouin zone, from acoustic to optical modes, taming the QTM effect as well as controlling the Orbach and Raman processes.

Aiming to further advance the synergy of experimental and theoretical methodologies in the research on Ln-SMMs, we report here a novel, highly interactive, and extensible computational tool called SlothPy. It is an open-source Python library under constant and vigorous development that aims to become a general-utility package for molecular magnetism, integrating many routines for *ab initio* computations and analysis of physical properties. It is documented at https://slothpy.org, with the code publicly available in the online repository, https://github.com/MTZ-dev/slothpy. The main objective of our program is to conduct simulations from the first-principles of various magnetic and related optical properties solely based on electronic structure modeling within popular quantum chemistry packages. This means that the results are derived only from matrices of related operators (e.g., spin, angular momentum, and electric dipole momentum) that are directly read from output files and processed by SlothPy. To demonstrate the potential of our new package, we report its application in the investigation of novel lanthanide molecular nanomagnets based on Nd^3+^/Ce^3+^ ions crystallizing with heteroligand pentacyanidocobaltates(III), [Co^III^(CN)_5_(X)]^3–^ (X = N_3_^–^, NO_2_^–^, I^–^) which results in dinuclear {[Ln^III^(H_2_O)_4_(2,2′-bpdo)_2_][Co^III^(CN)_5_(X)]}·3H_2_O (Nd/N_3_^–^, **1**; Ce/N_3_^–^, **2**; Nd/NO_2_^–^, **3**; Ce/NO_2_^–^, **4**; 2,2′-bpdo = 2,2′-bipyridine-1,1′-dioxide) molecules or {[Ln^III^(H_2_O)_5_(2,2′-bpdo)_2_][Co^III^(CN)_5_I]}·3H_2_O (Nd, **5**; Ce, **6**) ionic salts. We present their structures and field-induced slow magnetic relaxation processes, the latter studied experimentally with the support of previously reported relACs software,^32^ and theoretically by the *ab initio* calculations explored with SlothPy software, which were also used for the elucidation of their optical properties, including photoluminescence.

## Results and Discussion

### Synthesis and Structural Studies

**Figure 1:**
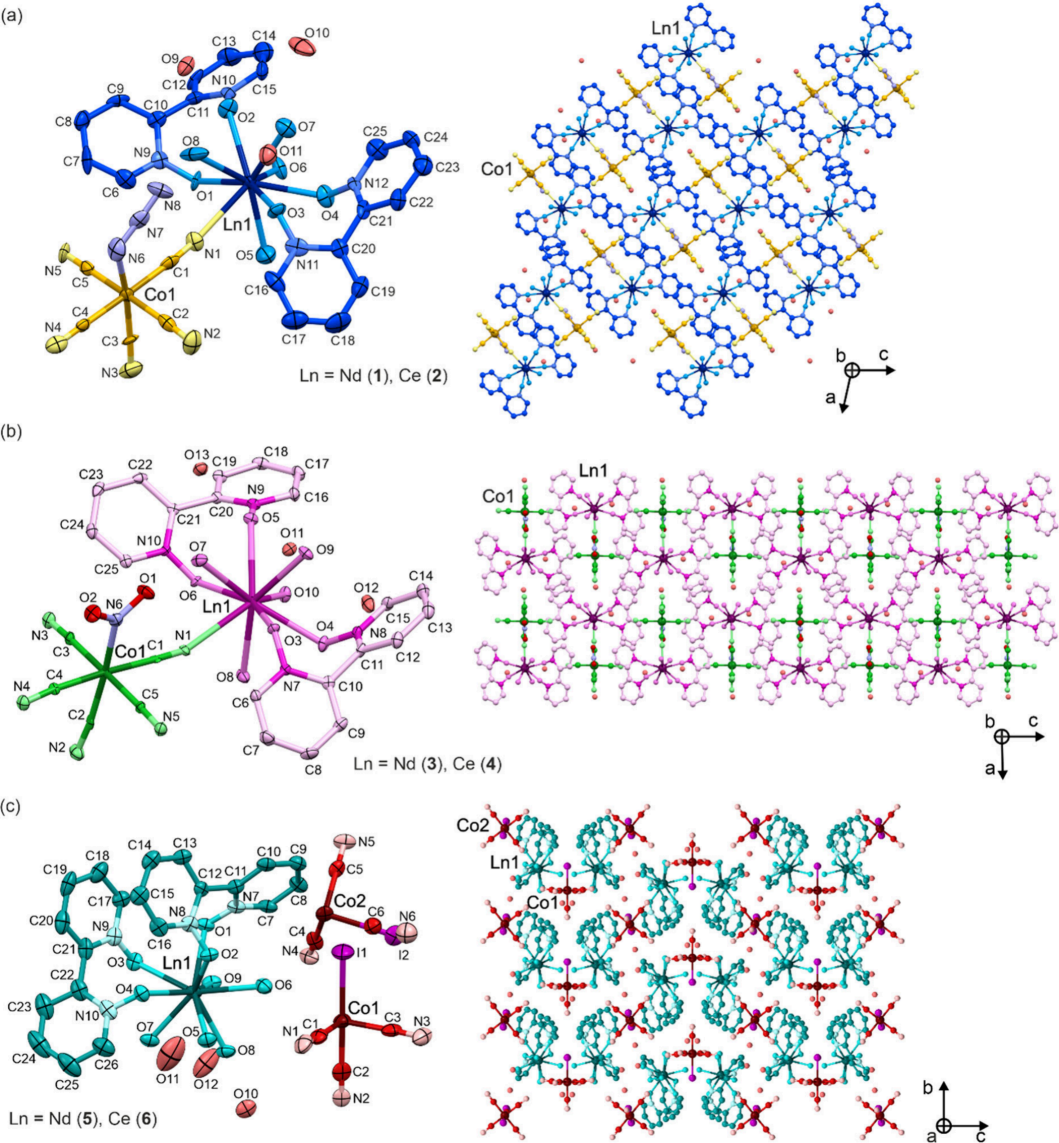
Structure of **1** and **2** (a, the example of **1**), **3** and **4** (b, the example of **4**), **5** and **6** (c, the example of **5**), including asymmetric units (left) and supramolecular networks (right). The color code used for each pair of compounds was further repeated in the figures illustrating their physical properties (see below).

All compounds, **1**–**6**, crystallize from the water–ethanol solution of lanthanide(III) chloride, 2,2′-bpdo ligands, and the pentacyanidocobaltate(III) salt (see Experimental Details and Table S1, Supporting Information). The obtained compounds were characterized by CHN elemental analysis, thermogravimetry, and IR spectra (Figures S1 and S2), their structures were determined by the single-crystal X-ray diffraction (SC-XRD) method (Figures 1 and S3–S8, Tables S2–S9), and the phase purity was checked by the powder X-ray diffraction (P-XRD) technique (Figure S9); moreover, their optical absorption and emission (the latter for NIR-emissive Nd^III^-containing **1**, **3**, and **5**) were examined (Figures S10–S13, Tables S12 and S13). **1** and **2** are dinuclear cyanido-bridged Nd^III^–[Co^III^(CN)_5_(N_3_)]^3–^ or Ce^III^–[Co^III^(CN)_5_(N_3_)]^3–^ molecules, respectively, involving nine-coordinated [Ln^III^(μ-NC)(H_2_O)_4_(2,2′-bpdo)_2_]^2–^ complexes of a capped square antiprism geometry (Figure 1a). **3** and **4** reveal similar structures, also being dinuclear Nd^III^–Co^III^ or Ce^III^–Co^III^ molecules, respectively, involving analogous nine-coordinated Ln(III) centers. They contain a different substituent in Co(III) complexes, having a nitrito-*N* ligand instead of the azido, which is also accompanied by the deformation of the Ln(III) polyhedron toward a tricapped trigonal prism (Figure 1b). **5** and **6** are ionic salts based on noncovalently bonded [Ln^III^(H_2_O)_5_(2,2′-bpdo)_2_]^3+^ and [Co^III^(CN)_5_I]^3–^ complexes as the ninth position of the capped-square-antiprismatic Ln(III) complexes is occupied by an aqua ligand instead of the bridging cyanido (Figure 1c). This also results in a different relative position of two 2,2′-bpdo ligands; they are aligned on the opposite sides of the complex in **1**–**4**, while close to each other in **5** and **6**. As they are the source of negative charges on donor atoms, such an alignment in **1**–**4** provides the axial ligand field suitable for SMM features. At the same time, the different arrangements in **5** and **6** are much worse for magnetic axiality. This finds the reflection in magnetic data (see below).

### Experimental Magnetic Characterization

**Figure 2:**
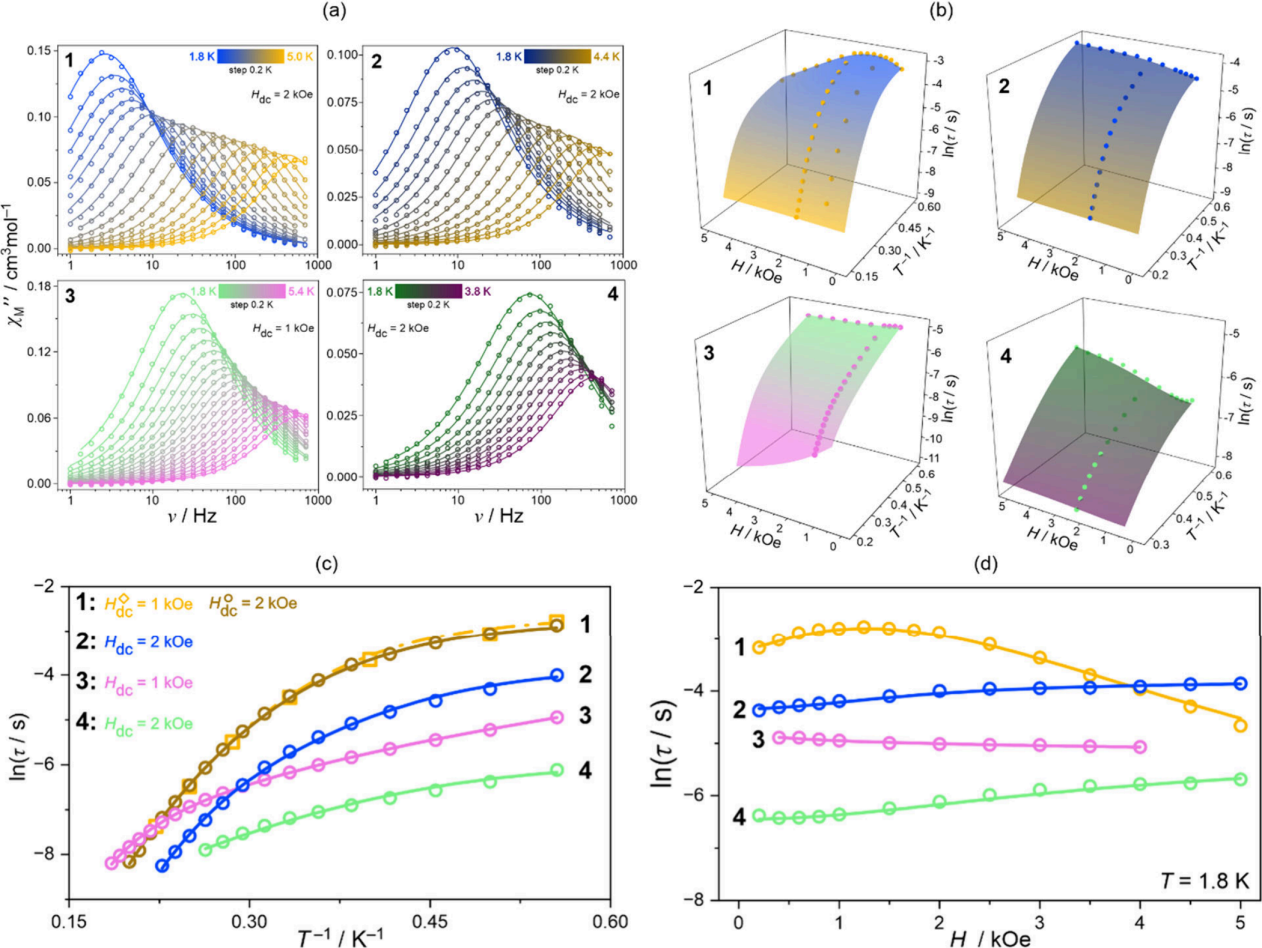
The representative set of *ac* magnetic curves of **1**–**4**: the frequency dependences of χ_M_″ at indicated temperatures, with the best-fit curves (eq 1). (a) The relaxation times in the function of *H* and *T*, shown with the best-fit multivariable surface (eq 2) and (b) the projections on the (c) τ(*T*) and (d) τ(*H*) dependences (circle points–experiment, solid lines–fits). The fitting procedures were performed using the relACs software.

We studied **1**–**6** using alternate-current (*ac*) magnetic measurements under variable direct-current (*dc*) magnetic field and temperature values (Figures 2 and S14–S25, Tables 1 and S14). The experimental data taken from the SQUID-type magnetometer were analyzed using the relACs program.^32^ We fitted complex frequency-dependent magnetic susceptibility χ_M_(ω) in the form of its real, χ_M_′, and imaginary, χ_M_″, components using the generalized Debye model, represented by the eq 1:
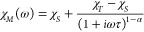
1Then we investigated the resultant multivariable dependence of the extracted relaxation times τ(*H*, *T*) considering the model captured within eq 2:^36−52^
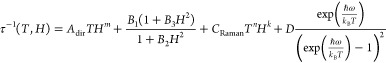
2The first term represents the so-called direct process that induces relaxation involving one-phonon absorption or emission between ground electronic states, taking into account their splitting in the external magnetic field.^39,41^ The second, known as the quantum tunneling of magnetization (QTM), depicts temperature-independent transition paths between two nearly degenerate states of opposite magnetization.^42^ It can be regarded as a two-state Landau–Zener-type process driven by internal hyperfine and dipolar magnetic fields due to the nuclear spins and magnetic momenta of surrounding atoms and complexes or by an externally applied oscillatory magnetic field.^43^ We model it with the Brons-van Vleck formula, where *B*_1_ is the zero-field tunneling rate, *B*_2_ represents the ability of the external static magnetic field to suppress the tunneling by bringing states out of resonance, and *B*_3_ is related to the concentration of the discussed surrounding sources of magnetic momenta.^40,44−46^ The third term represents a weakly field-dependent two-phonon Raman process responsible for demagnetization through a virtual excited state by absorption and emission of delocalized phonon modes mainly from the accessible acoustic spectrum.^36,39,47,48^ The last contribution is known as a local-mode process (LMP), and it is in the form of the Fourier transform of the two-phonon correlation function involving a frequency of definite pair of low-lying degenerate optical phonons simultaneously absorbed and emitted during a relaxation.^37,49−52^ Therefore, it is a second-order Raman process that involves a localized phonon mode with definite energy encapsulated in the parameter *ℏω*. We omitted a well-known Orbach-type relaxation in the fitting, which can be described by eq 3:
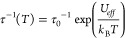
3Nevertheless, it was experimentally used to τ, within the high-temperature range, for comparison with our computations (Table 1, Figure S25). Precise rationalization of our decision and elucidation of all assigned processes will be given in connection with the results of *ab initio* calculations below. Now, we briefly discuss the experimental dependencies.

For none of the compounds did we observe a zero-*dc*-field signal in the χ_M_″(ν) for the investigated frequency range. A noticeable slow magnetic relaxation appears only under a static magnetic field. For **5** and **6**, maxima of χ_M_″(ν) fall out of the frequency window, indicating the poorest SMM performance (Figures S23 and S24). For **1**, as the magnetic field is increased at *T* = 1.8 K, there is a monotonic increase in the relaxation time, connected to the field-dependent QTM quenching realized by taking the two states of the ground doublet out of resonance (Figure S14). The relaxation time reaches its maximum at the optimal *dc* field of about 1 kOe. Then it starts to decrease rapidly due to the activation of a strong direct process with *m* = 3.120(5), which is close to the theoretically postulated value *m* = 4 for Kramers ions.^39,41^ The possible deviation is caused by the low-lying optical vibrational modes breaking the Debye approximation assuming linear phonon dispersion relation in the acoustic region.^38^ Then we studied the *T*-dependence of τ for the optimal field and the additional 2 kOe one (Figures 2 and S15). Similar theoretical models based on the Debye approximation predict Raman exponent *n* = 9 for Kramers ions whose relaxation is driven by the acoustic phonons.^38^ We found that no combination of QTM with the sole Raman process with *n* close to the postulated value can recreate the multidimensional fit of τ(*H*,*T*) (Figures S22 and S42); therefore, we turned to the description additionally utilizing the LMP process with *ℏω* = 8.9(9) cm^–1^. Keeping in mind that the *T*-power law can be regarded as originating from the sum of LMPs,^37^ in our interpretation, we split the overall two-phonon relaxation process into two parts, the usual Raman process, with *n* = 8.4(6) close to 9 involving the acoustic continuum of unlocalized phonons and a single LMP presumably corresponding to the lowest-lying optical phonon that drives the relaxation. The existence of localized optical phonons in this frequency window is later confirmed by DFT simulations (see Table S23). Moreover, we excluded potential field dependence of the two-phonon process (*k* = 0) due to the supplementary measurement for different magnetic field strengths.

Moving on to **2**, we observe the overall decrease in τ compared to **1** with a slightly stronger QTM as well as a noticeable change in the scheme of relaxation mechanisms. In particular, upon a closer inspection of the field dependence of τ (Figures 2 and S16), we see a continuous increase in the 0.2–5 kOe range without the direct process manifesting as was the case of **1** (Figures 2 and S14). On the other hand, for the arbitrarily selected *dc* field of 2 kOe, we investigated the temperature-dependent *ac* magnetic data, finding the LMP process with the critical frequency mode of *ℏω* = 9(1) cm^–1^ which is almost identical to those found in **1** (Table 1). The resulting Raman process is characterized by *n* = 9(1), which is also similar to those determined in **1**. Thus, it involves acoustic phonons and takes control in the range of the highest accessible temperatures around 4.4 K (Figure S17).

**Table 1:** Best-Fit Parameters for Relaxation Processes (Direct, QTM, Raman, and LMP; eq 2) in **1**–**4**, Obtained by Simultaneous Fitting of *H-* and *T*-Variable *ac* Magnetic Data Using the relACs Program (Figure 2), Together with the Arrhenius-type Fitting (eq 3) for the High *T*-Range *ac* Data, and the *Ab Initio* Calculated Energies of the First Excited Kramers Doublets

Fitting type	Parameter	**1**	**2**	**3**	**4**
overall fitting of Direct, QTM, Raman, and LMP processes (eq 2)	*A*_dir_/ s^–1^K^–1^Oe^–*m*^	1.2(7)·10^–10^	–	–	–
	*m*	3.120(5)	–	–	–
	*B*_1_/s^–1^	22(2)	66(3)	19(3)	533(72)
	*B*_2_/Oe^2–^	2.8(4)·10^–6^	3.8(1)·10^–7^	7.8(4)·10^–7^	1.3(4)·10^–7^
	*B*_3_/Oe^2–^	1.4(1) ·10^–6^	2.0(1)·10^–7^	1.9(2)·10^–6^	2.2(4)·10^–8^
	*C*_Raman_/s^–1^K^–n^Oe^–k^	0.004(4)	0.001(2)	1.09(2)·10^–7^	–
	*n*	8.4(6)	9(1)	8.2(2)	–
	*k*	0	0	1.41(6)	–
	*ℏω*/cm^–1^	8.9(9)	9(1)	3(1)	6(1)
	*D*/s^–1^	1.7(4)·10^3^	1(1)·10^4^	1.3(7)·10^3^	2.8(3)·10^4^
Arrhenius dependence fitting (eq 3)	*U*_eff_/cm^–1^	20.0(6)	17.9(4)	15.1(4)	6.9(3)
	τ_0_^–1^/s^–1^	8.45(22)·10^5^	1.30(26)·10^6^	1.99(24)·10^5^	3.66(56)·10^4^
*ab initio* energy of the 1st excited *m*_J_ state	*E*_1_/cm^–1^	128.28	298.25	132.15	181.44

For **3**, in field-dependent studies at 1.8 K (Figure S18), we observed unusually weak changes with an almost constant decrease of relaxation time. We could not explain such negligible variation in the relaxation time using the direct process term. It was necessary to consider the field dependence of the Raman process itself, resulting in *k* = 1.41(6), which is close to the predicted value of 2 for relaxations driven by the modulation of Zeeman interaction by phonons.^38^ Once again, temperature studies reveal the initial dominance of LMP (with a very low phonon frequency) and the increase of the Raman (*n* = 8.2) contribution with rising temperature (Figure S19). This effect is similar to the behavior observed in **1** and **2**. Finally, in **4** (Figures S19 and S20), we see a return to the monotonic increase of τ with the rising field associated with the quenching of QTM, which is relatively strong in the investigated field range. Due to the fastest relaxation times within the **1**–**4** series, it suffices to involve only the LMP process without the Raman one. However, compound **4** cannot be classified as an unusual case among the whole series as the Raman relaxation is probably operating, but it could not be reliably characterized due to the narrower temperature range of 1.8–3.8 that is accessible to be investigated by squid magnetometry (i.e., within the frequency range of 1–1000 Hz of *ac* field).

### *Ab Initio* Calculations and *SlothPy* Simulations of Magnetic Properties

**Figure 3:**
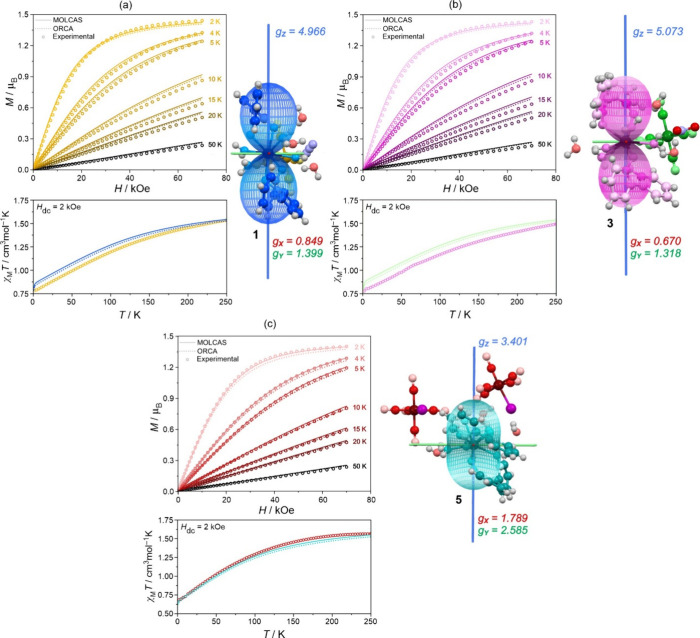
Experimental *dc* magnetic characteristics compared with the results of *ab initio* calculations and the related simulations of magnetic features for compounds (a) **1**, (b) **3**, and (c) **5**, including powder-averaged field dependences of magnetization at indicated temperatures with calculated curves using indicated programs and the visualization of molecular clusters used for calculations, shown with main magnetic anisotropy axes scaled by the corresponding values of pseudo-*g*-tensors of ground Kramers doublets with contours depicting shapes of directional magnetization for the *T* = 1.8 K and *H* = 2 kOe conditions. Simulations were done by using the SlothPy software.

To explain the magnetism of **1**–**6**, we performed relativistic calculations at the CASSCF (and NEVPT2 for **2** and **4**) level of theory using two codes, OpenMOLCAS and ORCA (see Computational Details for the theoretical background). Based on the results of the *ab initio* calculations, we simulated all magnetic and some spectroscopic properties using SlothPy software (see this section and the next one). Here, we discuss results only for Nd(III)-containing **1**, **3**, and **5**, leaving the results for Ce(III)-based **2**, **4**, and **6** in the Supporting Information. To validate the accuracy of theoretical models, we compared the simulated powder-averaged curves of magnetization in the function *dc* field with the experiment for the extensive *T*-range (2–50 K) and the χ_M_*T*(*T*) curves for *H*_dc_ = 2 kOe (Figures 3, S26, and S27, Table S15). The simulations are overall in good agreement with the experiment in the investigated range of fields and temperatures; however, perturbative NEVPT2 corrections, done for **2** and **4** (Figure S26), clearly improve the models. It is important to note that the featureless course of the χ_M_*T*(*T*) curves in low-*T* and field regimes and the apparent plateau reached for high fields in *M*(*H*) plots indicate the lack of magnetic exchange between the Ln(III) centers.

At this point, we can take a closer look at the calculated pseudo-*g*-tensors for the ground Kramers doublets as the magnitude of transversal *g*_*x*_ and *g*_*y*_ components can be correlated with the strength of the QTM effect (Tables S16–S22).^12^ All compounds are characterized by relatively large *g*_*x*_ and *g*_*y*_ components, explaining the lack of SMM behavior under the zero *dc* field. Nevertheless, all compounds, except **5**, have a considerable axial character with a dominant *g*_*z*_ contribution. This is reflected in the directional three-dimensional plots of magnetization (Figures 3 and S28–S31), in which the width of cross sections in the XY-plane can be treated as a direct measure of *g*_*x*_ and *g*_*y*_. The corresponding Z-main magnetic axes are oriented roughly along a bisection of O–Ln–O bonds with 2,2′-bpdo ligands for **1**–**4**, realizing postulates of rational axial ligand field design using opposite lying and electron-rich O-donors of organic ligands (Figures S28 and S29). The leading role of these organic ligands is also suggested by the shortest Ln–O distances that appear for the pair of O-atoms belonging to two oppositely aligned 2,2′-bpdo molecules (see Tables S4–S9 with the comment in the Supporting Information).

However, a closer insight into the relationship between the alignment of *Z*-axes and the coordination polyhedra of lanthanide centers brings a broadened description of the origin of anisotropy for the ground doublets (Figures S43–S46). All the embedded lanthanide(III) complexes can be considered as strongly distorted tricapped trigonal prisms (Table S11). With this interpretation, it is possible to draw the trigonal prismatic part of these polyhedrals, which are realized by four O-atoms from two 2,2′-bpdo ligands and two O-atoms from two coordinated water molecules (Figures S43–S46). Then it appears that the *Z*-axes are aligned close to the pseudo-*C*_3_ symmetry axes of the related trigonal prismatic part of these coordination polyhedra. This leads to the conclusion that the origin of the axial magnetic anisotropy in **1**–**4** is not straightforwardly related to the presence of two oppositely aligned 2,2′-bpdo as we found that the internal pseudo-*C*_3_ symmetry of the complex, especially of its critical part containing O-atoms from 2,2′-bpdo ligands, also plays a very important role. Interestingly, the alignment of the Z-main magnetic axis along the pseudo-*C*_3_ symmetry axis is much closer to the ideal case for compounds **1** and **2** based on two different lanthanide centers but containing the identical azido-cyanido Co(III) complex (Figures S43 and S44), whereas compounds **3** and **4**, incorporating nitrito-cyanido Co(III) metalloligands, reveal a strong deviation of the position *Z*-axis from the alignment of pseudo-*C*_3_ symmetry axis (Figures S45 and S46). In the latter case, the *Z*-axis is much closer to the direction defined by two O-atoms of oppositely aligned 2,2′-bpdo ligands. These differences can be correlated with the supramolecular interaction between the noncyanido ligand of the Co(III) complex and the ligands coordinated to the lanthanide center. In **1** and **2**, the azido group interacts rather weakly with the coordinated water molecule belonging to the mentioned trigonal prismatic part of the lanthanide complex. As a result, this trigonal prismatic entity is relatively weakly deformed, leading to their closer-to-ideal *C*_3_ symmetry that further contributes stronger to the generation of magnetic axiality governing the arrangement of the unfavorable alignment of the *Z*-axis. On the other hand, in **3** and **4**, the nitrito group reveals much stronger hydrogen bonds with the coordinated water molecules of the lanthanide complex; however, this interaction occurs for the water molecule that is not assigned to the trigonal prismatic part of the complex. Nevertheless, this effect leads to a large deformation of the mentioned trigonal prismatic entity for which the symmetry is far from the ideal *C*_3_ one. As a result, for **3** and **4**, the pseudo-*C*_3_ symmetry plays a weaker role than in **1** and **2**, and the *Z*-axis is more defined by two O-atoms of oppositely aligned 2,2′-bpdo ligands. In this context, compounds **5** and **6** reveal completely different, and much weaker, magnetic axiality as represented by the *g*-tensors for the ground doublets (Tables S20 and S21). This is due to the unfavorable alignment of two 2,2′-bpdo ligands, which are not positioned oppositely within the complex but placed on the same side of the coordination polyhedron (Figures S28 and S29). As a result, there is a lack of axially aligned ligands with negatively charged donor atoms and the anisotropy produced is poor. While the differences between compounds **1**–**4** and **5**–**6** are large due to the alignment of 2,2′-bpdo ligands, and the differences between azido-containing **1** and **2** and nitrito-containing **3** and **4** are significant due to the role of Co(III) metalloligands, one can consider the differences between Nd(III)-containing compounds (**1**, **3**) and Ce(III)-containing analogs (**2**, **4**). They are rather subtle, especially when considering the alignment of the main magnetic axes, which appear to be much more dependent on the used metalloligands and much less on the used lanthanide center. However, when comparing the pair of **1** and **2**, as well as further the pair of **3** and **4**, it can be observed that the *g*_*z*_ components of the ground doublets are always higher for Nd(III)-based compounds as expected from their terms on the ground electronic level. This can also be correlated to the higher deviation of the alignment of their main magnetic *Z*-axis from the direction given by the pseudo-*C*_3_ symmetry of the trigonal prismatic parts of the lanthanide complex. In other words, it appears that Ce(III) centers are here more sensitive to the structural deformations related to the supramolecular interactions between azido/nitrito groups of Co(III) complexes and coordinated water molecules of the second metal complex. Nevertheless, it can be stated as a conclusion for this discussion that the main role in governing the anisotropy in obtained compounds is related to the alignment of 2,2′-bpdo ligands and some of the coordinated water molecules, forming together the trigonal prismatic fragments of the coordination polyhedra, whereas the supporting modulating role can be assigned to the azido (in **1** and **2**) or nitrito (in **3** and **4**) groups of attached Co(III) complexes that amend the mentioned trigonal prismatic fragments affecting the resulting anisotropy.

The *Z*-axes point in the directions indicating the maxima of magnetization as a function of the orientation of the applied magnetic field, as presented in the 3-D plots in Figures 3 and S31. On the other hand, the Helmholtz energy dependence on the direction of the applied magnetic field (Figure S30) has an oblate donut-like shape perpendicular to the main magnetic axis along which the “hole”, i.e., a minimum, is located. The large difference between the dimensions of the resulting form within the *X*–*Y* plane and the dimension along the *Z*-axis represents the distinct anisotropy of the *g*-tensor.

Additionally, compounds **1**–**4** have contributions of maximal *m*_J_ = ± 9/2 and *m*_J_ = ± 5/2 states for Nd(III) and Ce(III), respectively, at a level of ca. 80% (Tables S16–S19). In this regard, the situation for **5** is clear; it is characterized by the poorest anisotropy (*g*_*x*_, *g*_*y*_, *g*_*z*_ = 1.7894, 2.5854, 3.4006) with a mixed ground doublet (Table S20) to which all accessible *m*_J_ states contribute due to the disadvantageous alignment of 2,2′-bpdo ligands. Therefore, even after applying the *dc* field, the QTM is too strong to observe the SMM effect. The ground doublet state of a mixed nature, with a large contribution from the *m*_J_ = ± 1/2 states, is predicted for **6**. The magnetic axiality seems, however, better than in **4**, suggesting that the observed fast relaxation is more facilitated by the phonon-assisted processes, which is possible since a heavy element (iodine) was introduced into this system.

Our theoretical findings can be confronted for **1**–**4** with subtle differences in their best-fit parameters for experimental relaxation processes. We attempted to compare the *B*_1_, i.e., a field-independent QTM part, which for Kramers ions should be proportional to *g*_*x*_^2^*H*_*x*_ + *g*_*y*_^2^*H*_*y*_.^13^ This can be done only among pairs **1**, **3** and **2**, **4** since the tunneling probability itself depends on magnetic momenta of states,^12^ which is higher for Nd(III) analogs with *m*_J_ = ± 9/2 contributions in the ground doublet compared to the maximal *m*_J_ = ± 5/2 for Ce(III). As a result, for **1** and **3**, we have (*B*_1_, *g*_*x*_, *g*_*y*_, *g*_*z*_) = (22(2) s^–1^, 0.8494, 1.3991, 4.9660), (19(3) s^–1^, 0.6696, 1.3177, 5.0727), while for **2** and **4**, (*B*_1_, *g*_*x*_, *g*_*y*_, *g*_*z*_) = (66(3) s^–1^, 0.4091, 0.6140, 3.9656), (533(72) s^–1^, 1.1045, 1.4093, 3.0674) find the reflection in the 3-D visualizations. Theoretical trends seem to agree with experiments showing slightly better axiality of **3** over **1** and a more pronounced difference in **2** over **4**, indicating the non-negligible role of subtle structural differences in the metric parameters within the pairs of compounds.

Moving on to the phonon-assisted temperature-dependent processes, our most straightforward information from the *ab initio* calculations consists of the energies of the first excited Kramers doublets. For **1**–**4**, they are situated in the 128–298 cm^–1^ range (Tables S16–S19) in opposition with the fittings of τ in the high-temperature range (eq 3), giving parameters of activation energies from 6.9 to 20.0 cm^–1^ (Table 1). Therefore, it is impossible to include the Orbach or TA-QTM processes, which give exponential activation dependence involving real states.

Thanks to SlothPy, we can also very precisely explore small states’ energy changes under a magnetic field of particular orientations, i.e., Zeeman splitting (Figures S32–S34 and Computational Details below). This is due to exact treatment with diagonalization of matrices in the full space of the *ab initio* calculated spin–orbit states. We see that the splitting for the ground multiplets of **1**–**6** is weak due to the poor axiality and small magnetic momenta of states, reaching orders of around 10 cm^–1^ in the high 10 T field. Thus, neglecting potential exchange and hyperfine interactions, there is certainly no states’ avoided level crossing that would be otherwise noticed as characteristic oscillations of τ(*H*). Analysis of the splitting in the field range of 0–5 kOe of the ground doublets of **1**–**4** (Figure S35) with field applied in the Z and XY-directions reveals that the most noticeable energy differences characterize **3** and **1**. This is in perfect correspondence with the field dependence of relaxation times (Figure 2), where there is a continuous decrease in τ for **2** and **4**. It means, according to their smaller splitting, that they are still in the QTM resonance energy window, which is reflected by the smaller values of *B*_2_ parameters, depicting the ability of the field to quench QTM, than for **1** and **3**.^40^ At the same time, **3** and **1** already reach points out of the resonance where the relaxation time starts to decrease due to the activation of phonon-assisted direct and field-dependent Raman processes, respectively.

Moreover, for such a small QTM difference postulated, e.g., between **1** and **3**, the corresponding difference in ln(τ) for *T*-dependent plots at low temperatures is too large to be elucidated without inclusion of the LMP process. For high-performance SMMs, it is typical to see QTM as a stable plateau at the lowest temperatures, which, combined with power-law (Raman) or exponential dependence (Orbach), can follow the experimental points. In our case, the plateau is never reached and plots have constant slopes that are impossible to recreate by combining Raman and QTM (Figure S42). Without conducting theoretical simulations, one could mistake the underlying processes by ascribing a stronger QTM for **3**, following logical reasoning based on relaxation times faster than **1**. But as we have seen, **3** is characterized by even better axiality and smaller transversal *g*-tensors than **1**. Therefore, we can deduce that the modulation of relaxation time at low temperatures is driven by the phonon-assisted LMP process controlled by the change of the vibrational structure introduced by substituting N_3_^–^ with NO_2_^–^ ligands. We believe that the change of *ℏω* = 8.9(9) cm^–1^ in **1** to 3(1) cm^–1^ in **3** is rather non-negligible, explaining, at least partially, a noticeable difference in both the magnitude of the relaxation times at low temperatures and the shape of the dependence. The latter is limited by the QTM for **1**, reaching a plateau around 2 K, while this is not the case for **3**. In the higher-*T* regime, where the Raman process becomes dominant (as many LMP-type processes start to be accessible), two curves even start to coincide. This change of shape is not found for the second pair of **2** and **4**, which coincides with the smaller change of *ℏω* and with the fact that **4** has drastically weaker axiality than **2**, as reflected in *B*_1_. By this analysis, we presented two pairs of analogs containing [Co^III^(CN)_5_(N_3_)]^3–^ and [Co^III^(CN)_5_(NO_2_)]^3–^ metalloligands that show slightly worse SMM properties for the former ion, but the possible explanation is a little bit different for Nd(III) and Ce(III) controlled by hard to distinguish LMP or QTM processes, respectively. This may suggest that the Co(III) metalloligand has a non-negligible yet subtle role in the slightly variable SMM features of attached lanthanide(III) centers.

Based solely on the static magnetic anisotropy, one could expect **3** to be characterized by relaxation times as long as those of **1**. However, this is not the case because of the whole phonon-driven dynamics, which is not considered in the static picture.^53^ It is especially important to consider ultralow-frequency optical and acoustic vibrations, whose significant role in spin relaxations was shown theoretically,^41^ and it is expected to be crucial for SMMs operating in the very low-temperature range of 2–5 K. We tried to use DFT methods to optimize the geometry of molecular fragments for **1**–**6** (Figures S28 and S29) and extract normal modes in the gas phase, but we obtained satisfactory results only for **1** and **2**. Simulated vibrational energies were rescaled (0.92 multiplier) to match the recorded IR spectra (Figure S41), which are usually used for validating the results from the DFT approach.^54^ We did not have experimental access to the THz range of vibrational spectra, but the results obtained from the DFT calculations could be employed to discuss the low-energy vibrations. The extracted energies of optical (at the Γ-point) vibrations (Table S23) start from 10 cm^–1^, matching parameters *ℏω* = 8.9(9) and 9(1) cm^–1^ extracted for LMP in **1** and **2**. The closest modes to those energies are the ground optical modes. They are visualized in Supporting Movies 1 and 2 and consist of the breathing of 2,2′-bpdo that leads to the modulation of the first coordination sphere of a lanthanide center. This experiment aims to show the existence of such low-lying optical modes. A comprehensive study of the nature of this kind of relaxation would include complex, periodic geometry optimization, simulation of phonons across the whole Brillouin zone covering their dispersion, and extracting spin-phonon coefficients along those distortions.^22−24^ Such studies are beyond the scope of this work. Nevertheless, we showed that the modification of [Co^III^(CN)_5_(X)]^3–^ metalloligands can slightly but non-negligibly alter relaxation times by the magnetic anisotropy control that modifies QTM, also contributing to the variation in the phonon structure especially compounds **1**–**4**, resulting in the different phonon-assisted relaxation mechanisms.

**Figure 4:**
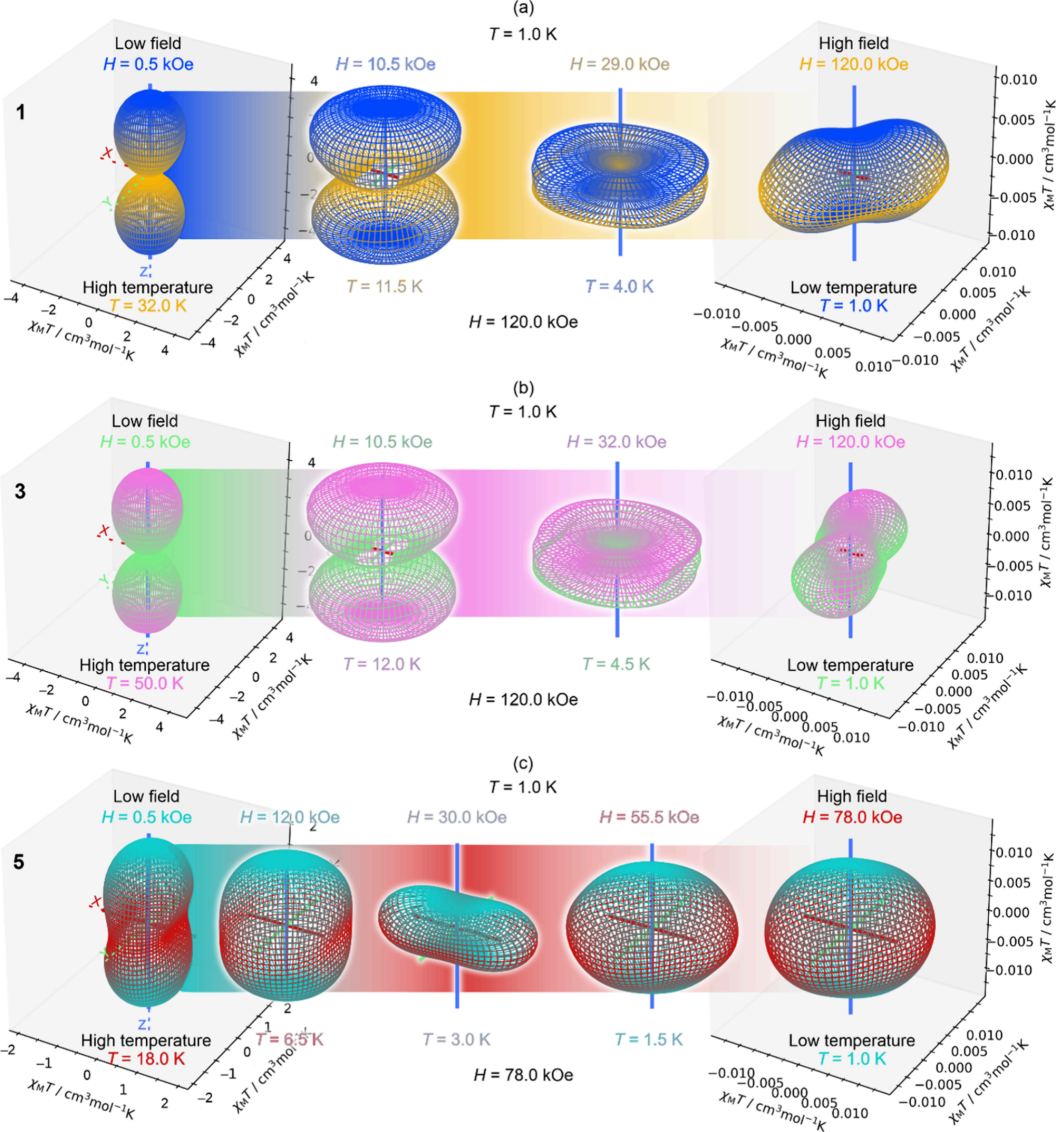
Evolution of the directional susceptibility in the form of its product with temperature, χ_M_*T*, for (a) **1**, (b) **3**, and (c) **5** under different *T*-conditions from the range of 1–50 K in a high magnetic field and various magnetic field magnitudes from the range of 0.5–120 kOe at the low temperature of 1 K. Simulations were done using the SlothPy software.

The SlothPy software allows us to explore the anisotropy of various quantities, such as Helmholtz and internal energies, magnetization, or magnetic susceptibility, together with susceptibility tensors and present them as functions of temperature and orientation of the *dc* magnetic field in three-dimensional plots (see Computational Details below). We present a representation of susceptibility changes for **1**, **3**, and **5** under different thermal conditions as directional plots for various fields (Figure 4 and Supporting Movies 3–6). We observe the *T*- and *H*-driven changes from the eas*y*-axis through easy-cone and easy-plane types of anisotropy of susceptibility. The changes result from the modulation of Zeeman splitting, the composition of states, and their magnetic momenta under the influence of the magnetic field, with thermal dependence governed by the changes in the Boltzmann population. We see that the anisotropy control can be realized with both temperature changes under a strong magnetic field and changes in a magnetic field at a very low temperature. Unsurprisingly, **5** (which is the worst SMM) displays the most exciting behavior in the related changes of magnetic anisotropy, managing to undergo even one extra complete “phase” transition compared to **1** and **3** in the given range of external stimuli. This feature, accessible to be easily investigated with our software, makes it a powerful tool for the exploration of anisotropy “phase space”, using different anisotropy descriptors.^55^ It also enables searching for different physical functionalities of compounds beyond slow magnetic relaxation, such as potential pseudocontact shift (PCS) agents, where the change in anisotropy of the susceptibility is desirable, or for various magnetic anisotropy switches (MAS).^56^

### Simulations of Optical Properties

**Figure 5:**
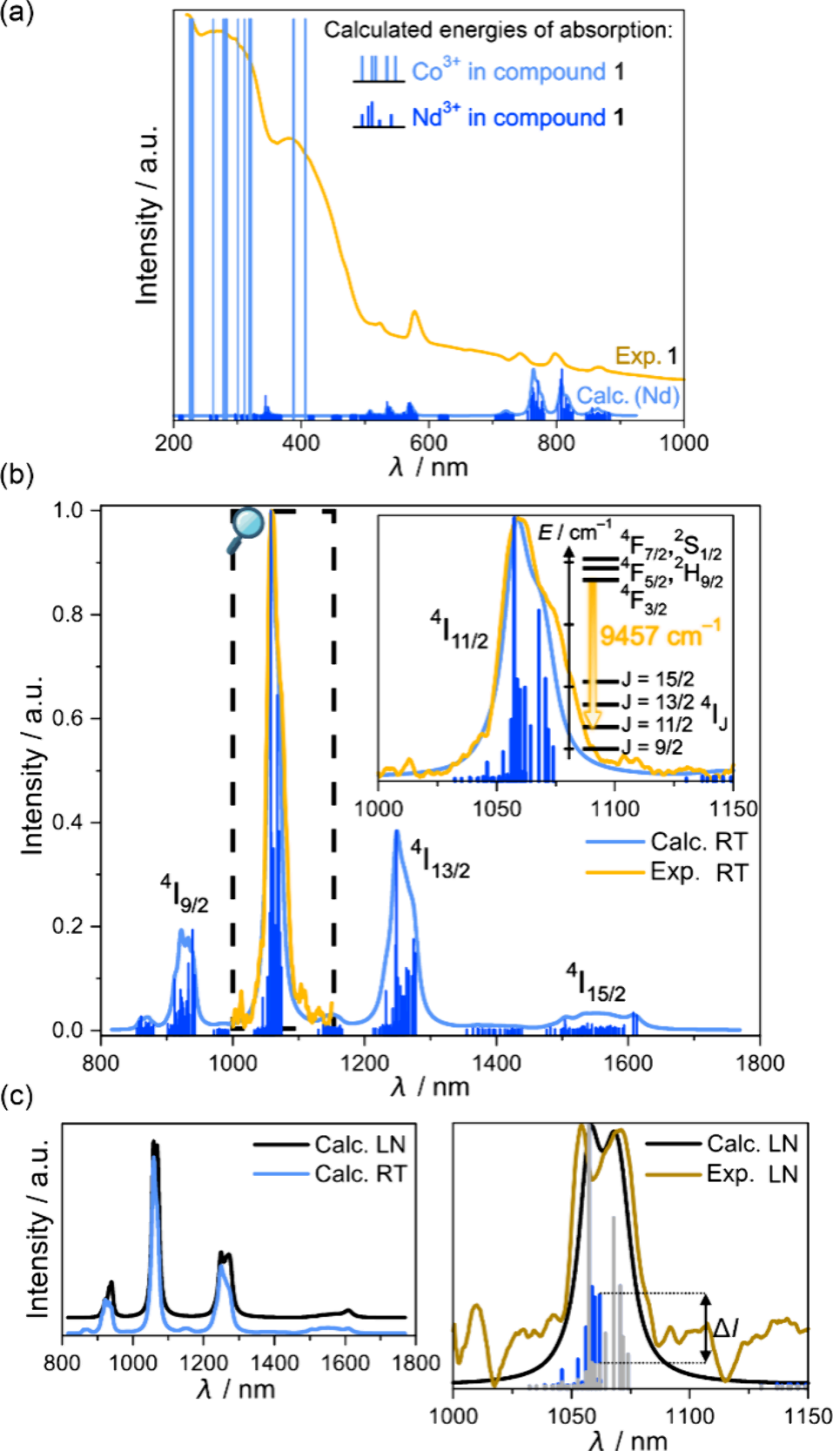
Experimental and calculated (*ab initio*) (a) light absorption and (b, c) luminescence of **1**, including calculated room-temperature (RT) wavelengths and intensities of absorption bands for Co^3+^ and Nd^3+^ ions in **1**, compared (a) with the experiment, calculated RT emission spectrum (Calc. RT) (b) with the magnified measured spectrum and (c, left) the comparison of the calculated emission spectra at RT and liquid nitrogen temperature (LN), shown with the part of the spectrum where the comparison with the experiment was done, for which the colored bars are the transition energies and intensities of the bands at RT and LN (*ΔI* represents the largest difference in the calculated intensity between RT and LN). In the inset of (b), we show the fragment of the Nd(III) energy level diagram for **1** with the indicated transition energy found in the experiment. Simulations were done using the SlothPy.

Further seeking multifunctionality within **1**–**6**, we present the results of SlothPy simulations regarding spectroscopic properties (see Computational Details for the theoretical background). **1**, **3**, and **5**, containing Nd(III), exhibit NIR photoluminescence (Figures S11–S13, Table S13). Using CASSCF results and subsequently calculated dipole moments between SOC states, used to obtain oscillator strengths, we simulated both UV–vis absorption and emission spectra (Figures 5 and S36–S40). We followed our previous methodology,^57^ extending it for temperature dependence within the simplest model scaling oscillator strengths with relative Boltzmann factors of states in the ground multiplet (for absorption) and emissive multiplet (for luminescence). Thus, the model can be regarded as a crude approximation neglecting relaxation, the vibration-driven dynamics, and intersystem crossing rates for excited states that are investigated in the ground state geometry with an assumption that the emissive multiplet manages to reach some quasi-thermal equilibrium described with the Boltzmann statistic. As this is insufficient for d-block transition metal ions, we found it useful for the initial exploration of lanthanide-based systems with well-screened f-electrons. Then we started with the simulation of energy levels at the CASSCF/NEVPT2 level of theory for [Co^III^(CN)_5_(X)]^3–^ precursors (Figure S36, 0.93 factor), including in the active space 5d orbitals of Co(III). We found that this is unable to reproduce the whole broad light absorption for **5**. Therefore, we assigned the missing region as charge-transfer transitions between I^–^ and Co^3+^, which are not elucidated in our treatment because of missing I-centered orbitals in our active space. Then we superimposed (Figure S37) experimental and CASSCF-simulated UV–vis spectra for Nd^3+^ ions at 300 K. A perfect match between weak f–f absorption bands is reached after energy scaling by 0.82. It is justified because simulations are performed for the gas phase, not including permittivity and refractive index corrections. Moreover, its lower value than for [Co^III^(CN)_5_X]^3–^ ions is rationalized by the fact that due to the size of the systems, we were not able to apply NEVPT2 corrections and enlarge the basis as was done for the investigated metalloligands.

Finally, we simulated normalized luminescence (Figures 5 and S38–S40) at RT and liquid nitrogen temperature (LN), taking into account oscillator strengths weighted with their Boltzmann factors for states beyond the emissive doublet, whose energy was set as a zero point for Boltzmann factors. Thus, at lower temperatures, we observe the decay of the hot-transition intensities, recreating the appearance of a second band (1075 nm) and the thermally induced change of its intensity, which can be helpful for eventual usage in luminescent thermometry.^33^

## Conclusions

In conclusion, this work provided an overview of the interplay of experimental and theoretical methods in elucidating intricate properties of lanthanide molecular nanomagnets, taking the novel family of Nd(III) and Ce(III) complexes attached to three different pentacyanidocobaltate(III) metalloligands as the example. The cyanido Co(III) complexes are diamagnetic and thus serve as molecular spacers to isolate magnetically anisotropic lanthanide centers in the crystal lattice, also contributing to the modulation of their first coordination spheres.^58,59^ By substituting the sixth position on Co(III) sides, we achieved the subtle modulation of the ligand field geometry and vibrational structure, thus contributing to the variation of the crystal field axiality and dynamical phonon-assisted magnetic relaxation pathways. Concerning the magnetic axiality, we found that the alignment of the Z-main magnetic axis is governed not only by the alignment of 2,2′-bpdo ligands, bearing crucial negatively charged O-donor atoms, but also by the pseudo-*C*_3_ symmetry of the trigonal prismatic fragments (containing O-atoms from 2,2′-bpdo ligands and two coordinated water molecules) that can be defined within the coordination polyhedra of obtained lanthanide complexes. Moreover, we found that the different substituent on the sixth position in Co(III) complexes (azido or nitrito) affects the anisotropy by amending the mentioned trigonal prismatic fragments through supramolecular interactions with coordinated water molecules.

In this work, we also introduced SlothPy software, an innovative open-source Python library, for intuitive and interactive scripting-like investigation of magnetic and related spectroscopic properties of such lanthanide-based molecular nanomagnets. It explores the relativistic *ab initio* calculations and, together with the previously demonstrated relACs program for analyzing experimental data,^32^ provides a tool for simulating a plethora of intriguing magnetic-related properties and theoretically explains the complex magnetic relaxation processes.

Thanks to these computational tools and their illuminating focus on intuitive visualization of investigated processes, we could rationalize a complicated mixture of relaxation processes, finding a crucial role for the local-mode process (LMP), which can be easily mistaken with the QTM effect. This is particularly important for moderately performing SMMs, such as the obtained compounds, where the phonons driving the magnetic relaxation can occur at very low frequencies. In this context, one can deduce the possible ways to improve the SMM characteristics as the critical LMPs, accelerating the magnetic relaxation, were identified as being related to the simultaneous movement of a few molecular components, mainly solvent molecules (both coordinated and those of crystallization) and aromatic rings of 2,2′-bpdo ligands (see Supporting Movies). Therefore, one way to slow down magnetic relaxation is to increase the energy of the above-mentioned LMPs which might be realized by stiffening the supramolecular framework exploring, e.g., 2,2′-bpdo-derivatives that can be involved in stronger noncovalent interactions, including more expanded aromatic components providing more efficient π–π stacking or the insertion of functional groups, such as hydroxy- or amino-, providing stronger hydrogen bonds. The other way will be to make attempts to crystallize the related lanthanide complexes without the solvent molecules of crystallization, which are weakly bonded and contribute to the disadvantageous low-energy vibrations. The strategy to remove them postsynthetically can be also an option, especially since it was presented to be an effective route to improve SMM characteristics of lanthanide(III)–hexacyanidobaltate(III) coordination systems,^33,34^ but it might be a fruitful route only in the case when the crystallinity and ordered arrangement of lanthanide(III) complexes will be preserved.

Besides the discussion of the LMP process, we also theoretically described the observed trends of QTM changes within the series and, therefore, reliably extracted the information about the phonon-assisted processes. Thus, we demonstrated significant limitations of considerations based purely on static ligand field anisotropy and axiality of low-lying states, which can overestimate the SMM performance, as they do not take into account phonon-assisted relaxation routes. We showcased the predictive power of SlothPy, providing the evolution of anisotropy of such properties as Helmholtz energy, magnetization, or magnetic susceptibility under variable (*T*,*H*)-conditions, along with spectroscopic studies on luminescence and absorption spectra in seek of potential and desirable multifunctionalities such as the applications as PCS, MAS, or in optical thermometry. The future challenge will be to broaden the potential of the SlothPy software toward performing the *in silico* simulations to get insight into the properties expected after the structural modification before the synthetic work. This will demand periodic structure optimization and calculations of, e.g., spin dynamics, taking into account the modified periodic structure. The work along this line is in progress.

## Computational Details

For the computational part of our work, we employed two popular quantum chemistry codes: OpenMolcas (ver. 23.06)^60−62^ and ORCA 5.0.3.^63,64^ In the first part, we conducted SA-CASSCF (i.e., State Average Complete Active Space Self-Consistent Field)-type calculations, including scalar relativistic effects within the two-component second-order Douglas-Kroll-Hess (DKH2) Hamiltonian framework. The *ab initio* calculations at this point were performed for the whole molecular fragments of **1**–**6**, including the closest-to-coordination-fragments water molecules of crystallization, using the crystal structure taken directly from the single-crystal X-ray diffraction (SC-XRD) experiments without any further geometry optimization (see the used molecular fragments in Figures S28 and S29). Our models adopted relativistic Atomic Natural Orbital basis sets of the ANO-RCC type with VTZP quality for central lanthanide ion, VDZP for atoms in the first coordination sphere, and VDZ for others employing Cholesky decomposition of ERI-s (electron repulsion integrals) with the 1.0 × 10^–8^ threshold in the OpenMolcas.^65−67^ Within the ORCA software, we used novel SARC2-DKH-QZVP basis sets for lanthanides^68^ and DKH-def2-TZVP for other atoms, together with RIJK resolution of identity approximation with relevant auxiliary basis sets for coulomb and exchange integrals to speed up the CASSCF calculations significantly.^69^ The active space was composed of seven 4f-orbitals of Ce(III) centers with 1 active electron, CAS(1in7), for **2**, **4**, and **6** or 3 active electrons, CAS(3in7) for Nd(III) analogs, i.e., **1**, **3**, and **5**. We worked on 7 doublet spin-adapted states for Ce(III) or 35 quartet and 112 doublet spin-adapted states for Nd(III), which arise from different possible active electrons’ distributions in 4f orbitals. In the SA-CASSCF (i.e., state-average) procedure, we assigned equal weights to all roots of the same multiplicity (OpenMolcas) or all of them (ORCA). Additionally, due to the small number of states to be corrected (7 for the case of Ce(III)-containing systems) and the absence of heavy iodine atoms (appearing in **5** and **6**), we managed to apply SC-NEVPT2 (Strongly Contracted N-Electron Valence State Second Order Perturbation Theory) corrections for **2** and **4** compounds containing Ce(III) centers but lacking iodine atoms.^70^ In the final step, the previously optimized “spin-free” states were mixed within the Restricted Active Space State Interaction (RASSI) submodule of OpenMolcas^71^ by Spin–Orbit-Coupling (SOC) within the atomic mean-field (AMFI) approximation^72^ and the corresponding method adopting quasi-degenerate perturbation theory in the ORCA SOC submodule. The resulting matrices of spin, angular, and dipole momenta between SOC states (14 for Ce(III) and 364 for Nd(III) centers), along with the SOC coupling matrix itself, are then read and used by the newly designed SlothPy software to simulate various magnetic and spectroscopic properties.

In this regard, consideration of magnetic properties within the SlothPy program is based on the construction of Zeeman Hamiltonian by adding to the *ab initio* SOC matrix Zeeman interaction with the magnetic fields of various magnitudes |*B⃗*| and orientations: *B⃗* = |*B⃗*| · *e⃗*, where *e⃗* is a unit directional vector (eq 4):
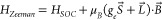
4In the next step, *H*_*Zeeman*_ is diagonalized using unitary transformation *U* (eq 5):
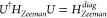
5The matrix *H*_*Zeeman*_^*diag*^ in the new basis has diagonal energies *E*_*i*_(*B⃗*) of the Zeeman states that can be directly used for visualization of the Zeeman splitting or calculation of the Helmholtz free energy (eq 6):
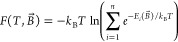
6Magnetization is obtained as the directional derivative of *F* by the well-known formula (eq 7):
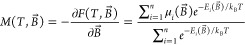
7Here, we take the advantage of the fact that the Zeeman interaction, *μ*_*B*_(*g*_*e*_*S⃗* + *L⃗*) · *B⃗* , is linear in *B⃗*, therefore  where in a given applied field *μ*_*i*_(*B⃗*) are diagonal elements of (eq 8):
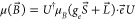
8i.e., the initial magnetic momenta in the direction of *e⃗* transformed by the previously designated unitary transformation to the new basis. Magnetic susceptibility is further calculated using numerical differentiation of *M*(*T*, *B⃗*) using finite difference method (eq 9):^73,74^
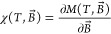
9

To recreate powder measurements, the magnetization is averaged over a grid of directions over the hemisphere, while the susceptibility is averaged over x, y, and z directions of the magnetic field as it is sufficient for the second-rank tensor.

Spectroscopic intensities are simply obtained as proportional to the oscillator strength *f*_*AB*_ between states for which transition occurs and is calculated from the *ab initio* dipole moments between SOC states ⟨*A*|*R*_*α*_|*B*⟩ using eq 10:
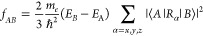
10Each *f*_*AB*_ is then, scaled taking into account the Boltzmann population of states, i.e., , where for absorption spectra, *E*_0_ is an energy of the ground state, while for emission (photoluminescence), *E*_0_ is an energy of the ground state of the emissive multiplet, for Nd(III) ^4^F_3/2_, and only states higher in energy are considered with transition to states with lower energies, for Nd(III), they belong to ^4^I_15/2_, ^4^I_13/2_, ^4^I_11/2_, and ^4^I_9/2_.

SlothPy software is a young package that is under constant, vigorous development. Its rich documentation, showcasing its current abilities, can be found on the official Web site https://slothpy.org/, which is dynamically updated with each release. We hope that during its evolution, it will become an attractive alternative to widely used spin Hamiltonian codes such as Phi^75^ or EasySpin,^76^ offering similar, performant, and extended capabilities of simulations from the first-principles to the state-of-the-art SINGLE_ANISO^77^ module for researchers in the field of molecular magnetism who prefer to work in a Python environment that seamlessly integrates into their custom data pipelines.

All magnetic and luminescence simulations, the results of which are gathered in figures and tables of this article’s main text and Supporting Information, were conducted using SlothPy.

Additionally, to support the elucidation of the UV–vis absorption spectra region dominated by d–d electronic transitions within the applied cyanido metalloligands, we performed similar computations at the NEVPT2 theory level and with TZVPP basis quality for [Co^III^(CN)_5_(X)]^3–^ (X = N_3_, NO_2_, I) fragments of **1**, **3**, and **6** with five 3d orbitals in active space and six active electrons , CAS(6in5).

Finally, to make a connection to the postulated magnetic relaxation involving low optical modes (within the LMP process), we tried to optimize the geometry of the investigated molecules in the gas phase using fragments consistent with the CASSCF approach but utilizing DFT with PBE0^78^ functional and D3 dispersion correction^79^ with ORCA software. We only managed to obtain reliable results of the optimization for **1** and **2**, and with the possibility of calculating the Hessian analytically, we were able to extract normal modes and their frequencies only.

## Supporting Information Available

Experimental details, basic characterization with detailed structural studies, complete *dc* and *ac* magnetic characteristics, expanded results of the *ab initio* calculations with the related simulations of physical properties, their comparisons with spectroscopic characteristics, and the results of DFT calculations for IR spectra (PDF)
Supporting movie visualizing key optical vibrational modes for slow magnetic relaxation processes (MP4)
Supporting movie visualizing key optical vibrational modes for slow magnetic relaxation processes (MP4)
Supporting movies visualizing the evolution of magnetic susceptibility under different thermal conditions and magnetic field magnitudes (ZIP)
