# Supplementary material for: Synergy of Experiment and Broadened Exploration of Ab Initio Calculations for Understanding of Lanthanide–Pentacyanidocobaltate Molecular Nanomagnets and Their Optical Properties
Source: Inorg Chem. 2024 Sep 2;63(41):19213–26. doi: 10.1021/acs.inorgchem.4c02793 (PMC11483780; doi:10.1021/acs.inorgchem.4c02793)
Supplement: Supplementary file 1 — ic4c02793_si_001.pdf [file ic4c02793_si_001.pdf]

SUPPORTING INFORMATION

Synergy of Experiment and Broadened Exploration of Ab Initio Calculations for Understanding of Lanthanide–Pentacyanidocobaltate Molecular Nanomagnets and their Optical Properties

Mikolaj Zychowicz,[1,2,]* Hubert Dzielak,[1] Jan Rzepiela,[1,2] Szymon Chorazy[1,]*

[1]Faculty of Chemistry, Jagiellonian University, Gronostajowa 2, 30-387 Krakow, Poland
[2]Doctoral School of Exact and Natural Sciences, Jagiellonian University, Lojasiewicza 11, 30-348 Krakow, Poland

*Corresponding authors: simon.chorazy@uj.edu.pl, mikolaj.zychowicz@uj.edu.pl

Experimental Details including selected details of basic characterization of compounds **1**–**6**. (Table S1)	S3
Thermogravimetric curves (TG) for crystalline samples of compounds **1**–**6**. (Figure S1)	S8
Infrared (IR) absorption spectra of selected crystals of compounds **1**–**6**. (Figure S2)	S9
Crystal data and structure refinement parameters for compounds **1**, **2,** and **3**. (Table S2)	S10
Crystal data and structure refinement parameters for compounds **4**, **5,** and **6**. (Table S3)	S11
Additional structural views of **1** and **2**. (Figure S3)	S12
The views on the asymmetric units of **1** and **2**. (Figure S4)	S13
Selected bond lengths and angles in the crystal structure of compound **1**. (Table S4)	S14
Selected bond lengths and angles in the crystal structure of compound **2**. (Table S5)	S15
Additional structural views of **3** and **4**. (Figure S5)	S16
The views on the asymmetric units of **3** and **4**. (Figure S6)	S17
Selected bond lengths and angles in the crystal structure of compound **3**. (Table S6)	S18
Selected bond lengths and angles in the crystal structure of compound **4**. (Table S7)	S19
Additional structural views of **5** and **6**. (Figure S7)	S20
The views on the asymmetric units of **5** and **6**. (Figure S8)	S21
Selected bond lengths and angles in the crystal structure of compound **5**. (Table S8)	S22
Selected bond lengths and angles in the crystal structure of compound **6**. (Table S9)	S23
Comment to Figures S3–S8 and Tables S4–S9 – detailed structural data of **1**–**6** and its implications to the expected magnetic anisotropy of embedded lanthanide(III) centers.	S24
Results of Continuous Shape Measure analysis for six-coordinated Co(III) complexes in **1–6**. (Table S10)	S25
Results of Continuous Shape Measure analysis for nine-coordinated Ln(III) complexes in **1–6**. (Table S11)	S26
Comparison of experimental and calculated P-XRD patterns of the powder samples of **1**–**6**. (Figure S9)	S27
Solid-state UV-vis-NIR absorption spectra of **1**–**6** and assignment of the peaks related to f-f electronic transitions of Nd(III) centers. (Figure S10, Table S12).	S28
Solid-state excitation and emission spectra of compound **1**. (Figure S11)	S29
Solid-state excitation and emission spectra of compound **3**. (Figure S12)	S30
Solid-state excitation and emission spectra of compound **5**. (Figure S13)	S31
Assignment of vis-to-NIR excitation peaks of **1**, **3**, and **5** to Nd(III) f-f electronic transitions. (Table S13)	S32
Field (*dc*) variable alternating-current (*ac*) magnetic characteristics of **1** at T = 1.8 K. (Figure S14)	S33
Temperature variable alternating-current (*ac*) magnetic characteristics of **1** at H_{dc} = 2 kOe. (Figure S15)	S34
Field (*dc*) variable alternating-current (*ac*) magnetic characteristics of **2** at T = 1.8 K. (Figure S16)	S35
Temperature variable alternating-current (*ac*) magnetic characteristics of **2** at H_{dc} = 2 kOe. (Figure S17)	S36
Field (*dc*) variable alternating-current (*ac*) magnetic characteristics of **3** at T = 1.8 K. (Figure S18)	S37
Temperature variable alternating-current (*ac*) magnetic characteristics of **3** at H_{dc} = 1 kOe. (Figure S19)	S38
Field (*dc*) variable alternating-current (*ac*) magnetic characteristics of **4** at T = 1.8 K. (Figure S20)	S39
Temperature variable alternating-current (*ac*) magnetic characteristics of **4** at H_{dc} = 2 kOe. (Figure S21)	S40
Three-dimensional views of the hypersurface of multivariable $\tau(H, T)$ dependences of **1**–**4**. (Figure S22)	S41
Representative *ac* magnetic characteristics of **5**. (Figure S23)	S42
Representative *ac* magnetic characteristics of **6**. (Figure S24)	S43
Results of the fitting of the high-T range of the T-dependences of relaxation times of **1**–**4** using only the Arrhenius-type contribution related to a possible Orbach relaxation process. (Figure S25)	S44
The best-fit parameters of the 3-D fitting of both H- and T-dependent relaxation times of **1**–**4**. (Table S14)	S45
Experimental *dc* magnetic characteristics of **1**–**4**, compared with the calculated curves. (Figure S26)	S46
Experimental *dc* magnetic characteristics of **5** and **6**, compared with the calculated curves. (Figure S27)	S47

Supplementary information regarding *dc* magnetic measurements for **1**–**6** and their analysis. (Table S15) S48
Energy splitting and pseudo-*g*-tensor components of the $^4I_{9/2}$ ground-state term of Nd^{III} centers in **1**, shown with the compositions of ground Kramers doublets, calculated in MOLCAS and ORCA. (Table S16) S49
Energy splitting and pseudo-*g*-tensor components of the $^2F_{5/2}$ ground-state term of Ce^{III} centers in **2**, shown with the compositions of ground Kramers doublets, calculated in MOLCAS and ORCA. (Table S17) S50
Energy splitting and pseudo-*g*-tensor components of the $^4I_{9/2}$ ground-state term of Nd^{III} centers in **3**, shown with the compositions of ground Kramers doublets, calculated in MOLCAS and ORCA. (Table S18) S51
Energy splitting and pseudo-*g*-tensor components of the $^2F_{5/2}$ ground-state term of Ce^{III} centers in **4**, shown with the compositions of ground Kramers doublets, calculated in MOLCAS and ORCA. (Table S19) S52
Energy splitting and pseudo-*g*-tensor components of the $^4I_{9/2}$ ground-state term of Nd^{III} centers in **5**, shown with the compositions of ground Kramers doublets, calculated in MOLCAS and ORCA. (Table S20) S53
Energy splitting and pseudo-*g*-tensor components of the $^2F_{5/2}$ ground-state term of Ce^{III} centers in **6**, shown with the compositions of ground Kramers doublets, calculated in MOLCAS and ORCA. (Table S21) S54
Energy splitting and pseudo-*g*-tensor components of the $^2F_{5/2}$ term of Ce^{III} centers in **2** and **4**, shown with the compositions of ground Kramers doublets, calculated using NEVPT2 method in ORCA. (Table S22) S55
Fragments of the crystal structures, containing the magnetic Nd(III) center, of **1**, **3**, and **5**, which were used for the *ab initio* calculations shown with the determined main magnetic axes. (Figure S28) S56
Fragments of the crystal structures, containing the magnetic Ce(III) center, of **2**, **4**, and **6**, which were used for the *ab initio* calculations shown with the determined main magnetic axes. (Figure S29) S57
Dependences of Helmholtz energy of **1**–**6** on the direction of *dc* field, with the main magnetic axes of the ground Kramers doublets, simulated using the SlothPy software from *ab initio* calculations. (Figure S30) S58
Dependences of magnetization of **1**–**6** on the direction of *dc* field, with the main magnetic axes of the ground Kramers doublets, simulated using the SlothPy software from *ab initio* calculations. (Figure S31) S59
Zeeman splitting of Kramers doublets within the ground multiplets of **1** and **2** in the function of magnetic field strength, averaged over the grid and applied along the Z main magnetic axis. (Figure S32) S60
Zeeman splitting of Kramers doublets within the ground multiplets of **3** and **4** in the function of magnetic field strength, averaged over the grid and applied along the Z main magnetic axis. (Figure S33) S61
Zeeman splitting of Kramers doublets within the ground multiplets of **5** and **6** in the function of magnetic field strength, averaged over the grid and applied along the Z main magnetic axis. (Figure S34) S62
Zeeman splitting of the ground Kramers doublet in the function of the magnetic field applied in the direction of Z and X+Y magnetic axes for **1**–**4**, and the energy differences within the doublet. (Figure S35) S63
Ab initio calculated wavelengths of light absorption bands for Co^{3+} ions in $[Co^{III}(CN)_5(N_3)]^{3-}$ complexes of **1**, $[Co^{III}(CN)_5(NO_2)]^{3-}$of **2** (b), and $[Co^{III}(CN)_5I]^{3-}$of **3**, compared with the experimental solid-state UV-vis-NIR absorption spectra of the precursor complexes. (Figure S36) S64
Ab initio (CASSCF) calculated wavelengths of light absorption bands for Co^{3+} and Nd^{3+} ions embedded in **1**, **3**, and **5**, compared with their experimental solid-state UV-vis-NIR spectra. (Figure S37) S65
The *ab initio* calculated emission spectrum of **1** at room temperature with the magnification of the area where the experimental spectrum was recorded, the comparison of the calculated emission spectra at RT and liquid nitrogen temperature (LN), compared also with the experiment at LN. (Figure S38) S66
The *ab initio* calculated emission spectrum of **3** at room temperature with the magnification of the area where the experimental spectrum was recorded, the comparison of the calculated emission spectra at RT and liquid nitrogen temperature (LN), compared also with the experiment at LN. (Figure S39) S67
The *ab initio* calculated emission spectrum of **5** at room temperature with the magnification of the area where the experimental spectrum was recorded, the comparison of the calculated emission spectra at RT and liquid nitrogen temperature (LN), compared also with the experiment at LN. (Figure S40) S68
DFT-calculated and experimental infrared (IR) spectra of compounds **1** and **2**. (Figure S41) S69
DFT-calculated energies of vibrational modes for compounds **1** and **2**. (Table S23) S70
Comparison of curves representing different potential combinations of typical magnetic relaxation processes (Orbach, QTM, Raman, LMP) with experimental relaxation times for **3**. (Figure S42) S72
Description of Supporting Movies. S73
Additional discussion on scaling of the computational results to the experimental data. S74
Additional visualization of the *ab-initio*-calculated main magnetic axes in the molecule of **1**. (Figure S43) S75
Additional visualization of the *ab-initio*-calculated main magnetic axes in the molecule of **2**. (Figure S44) S76
Additional visualization of the *ab-initio*-calculated main magnetic axes in the molecule of **3**. (Figure S45) S77
Additional visualization of the *ab-initio*-calculated main magnetic axes in the molecule of **4**. (Figure S46) S78
References to the Supporting Information. S79

Experimental Details

Materials

Cobalt(III) chloride hexahydrate, sodium azide, 2,2'-bipyridine-1,1'-dioxide, ammonium chloride, aqueous ammonia solution (conc. ca 25%), potassium cyanide, hydroiodic acid, glacial acetic acid, hydrochloric acid, absolute ethanol (99.9% purity), potassium nitrite were purchased from commercial sources (TCI, Merck and others) and used as received unless otherwise noted.

Synthesis of Metal-Cyanido Precursors

The syntheses of polycyanidometallate precursors were performed starting from the previously published procedures that were adapted and optimized. This synthetic part included the preparation of pentaamina-azidocobalt(III) chloride,[S1] which was further used for the synthesis of potassium azidopentacyanidocobaltate(III).[S2] The latter was used for the synthesis of bimetallic compounds with lanthanide ions (see below) but also it was employed in the preparation of two other precursors, namely potassium pentacyanidonitrito-*N*-cobaltate(III) and potassium pentacyanidoiodocobaltate(III) complexes.[S3] The scheme illustrating the mentioned metal-cyanido precursors, as well as the main synthetic conditions and reactant used, is presented below (Scheme S1). The optimized synthetic procedures of metal-cyanido precursors are described below.

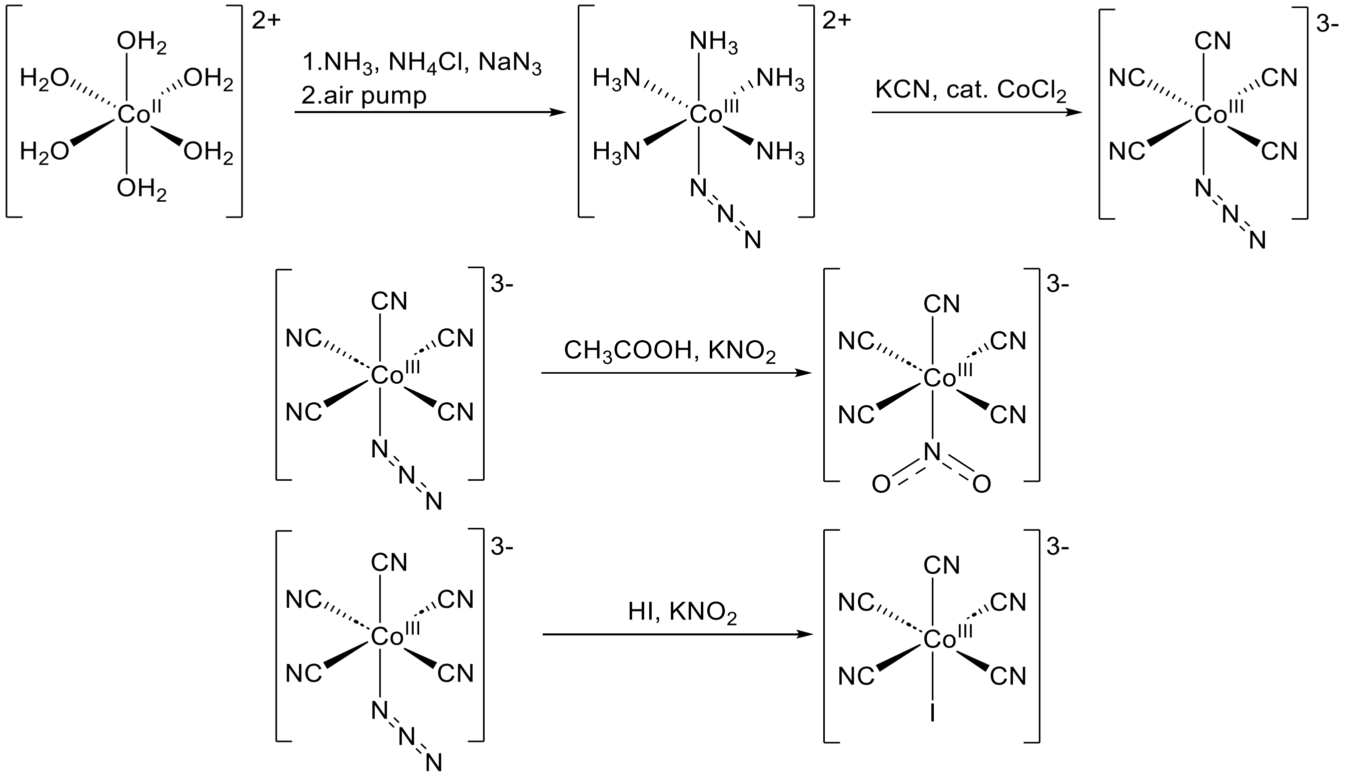

Scheme 1. The graphical summary of synthetic pathways employed in the preparation of three metal-cyanido precursors (given on the right side of each reaction) that were further used in the syntheses of compounds **1**–**6**.

Synthesis of $K_3[Co^{III}(CN)_5(N_3)]\cdot 2H_2O$

Colorless solution of sodium azide (6.01 g, 92.4 mmol, 3.7 eq.), aqueous ammonia (19.2 mL of 25% solution by mass), and ammonium chloride (12.00 g, 224.3 mmol, 8.9 eq.) in 36 mL of distilled water was slowly added while stirring to the red solution of cobalt(II) chloride (5.99 g, 25.2 mmol, 1.0 eq.) in 15 mL of distilled water. Upon this addition, the color of the solution changed from dark pink to brown. Then, the air was bubbled for 10 hours at ambient temperature through the resulting solution. A color change was again observed from brown to dark pink. The air-flow setup was removed, and the solution was heated at 80 °C on a hotplate while stirring for an hour. After cooling down, the solution was placed in a fridge at 4 °C for 5 hours. The suspension was vacuum-filtered, and the resulting solid was washed first with 40 mL of 4 M hydrochloric acid (10 mL of concentrated HCl and 30 mL of

distilled water) and secondly with 50 mL of ethanol. Dark pink crystalline powder of $[Co^{III}(N_3)(NH_3)_5]Cl_2$ was obtained after drying. Yield (based on Co): 4.20–5.94 g (63.6–91.7%, depending on the synthesis). Recrystallization was performed to confirm the product's structure and composition by single-crystal X-ray diffraction (SC-XRD) method (details of the crystal structure not reported here). To do this, 200 mg of the powder sample of $[Co^{III}(N_3)(NH_3)_5]Cl_2$ was dissolved at approx. 60 °C in a minimal amount of distilled water, and the solution was left at 4 °C overnight. Elemental analysis. Calculated for the formula found by SC-XRD (i.e., $[Co^{III}(N_3)(NH_3)_5]Cl_2$, molar mass: 257.01 g/mol): C: 0.0%, N: 43.6%, H: 5.9%. Experimentally found: C: 0.0%, N: 43.3%, H: 5.9%.

To the suspension of $[Co^{III}(N_3)(NH_3)_5]Cl_2$ (3.50 g, 13.6 mmol, 1.0 eq.) in 21 mL of distilled water, a light brown solution containing potassium cyanide (4.79 g, 73.6 mmol, 5.4 eq.) and the catalytic amount of cobalt(II) chloride hexahydrate (21 mg, 0.16 mmol) in 15 mL of water was added with stirring. A color change from dark pink to dark yellow was observed. The reaction was left keeping a continuous stirring for 45 minutes, after that 53 mL of ethanol was added, and the reaction mixture was left in the fridge at 4 °C for crystallization. After 24 hours, dark yellow crystals of $K_3[Co^{III}(CN)_5(N_3)]\cdot 2H_2O$ were formed. They were vacuum-filtered, washed with cold ethanol, and dried. Yield: 3.15–4.08 g (60.3–78.0%, depending on the synthesis). The crystal structure and composition were confirmed by the SC-XRD analysis (details of the crystal structure not reported here). Elemental analysis. Calculated for the formula found by SC-XRD (i.e., $K_3[Co^{III}(CN)_5(N_3)]\cdot 2H_2O$, molar mass: 384.37 g/mol): C: 15.6%, N: 29.1%, H: 1.0%. Experimentally found: C: 15.2%, N: 28.7%, H: 1.0%.

Synthesis of $K_3[Co^{III}(CN)_5(NO_2)]$

To the solution of $K_3[Co^{III}(CN)_5(N_3)]\cdot 2H_2O$ (1.50 g, 3.9 mmol, 1.0 eq.) in 3 mL of glacial acetic acid and 5 mL of water, solid potassium nitrite (1.01 g, 11.9 mmol, 3.1 eq.) was added in the portion-wise manner. The mixture was vigorously stirred during the addition of reactants, and gases evolved. A color change from dark yellow to lighter yellow was observed. Then, the reaction mixture was heated on a hotplate to 45 °C and stirred for 20 minutes. After the solution cooled down, 10 mL of cold (4 °C) ethanol was added, which led to the precipitation of the light yellow powder of $K_3[Co^{III}(CN)_5(NO_2)]$. The product was vacuum-filtered and dried. Yield: 1.29–1.31 g (93.9–95.3%, depending on the synthesis). Recrystallization was performed; 30.0 mg of product was dissolved in 0.44 mL of distilled water, and then 0.5 mL of ethanol was added. After 24 hours in the fridge at 4 °C, the crystals of $K_3[Co^{III}(CN)_5(NO_2)]$ formed. Their crystal structure was determined by the SC-XRD analysis (details of the crystal structure not reported here). Elemental analysis. Calculated for the formula found by SC-XRD (i.e., $K_3[Co^{III}(CN)_5(NO_2)]$, molar mass: 352.32 g/mol): C: 17.1%, N: 23.8%, H: 0.0%. experimentally found: C: 17.2%, N: 23.2%, H: 0.3%. The discrepancy in the elemental analysis (in the N- and H-percentages) is due to the hygroscopic nature of this precursor which gradually absorbs a small amount of water from the air (it can be estimated to be less than 0.5 H_2O per the formula unit); however, when stored in the dried place, the composition of this precursor can be considered as anhydrous.

Synthesis of $K_3[Co^{III}(CN)_5I]$

To the solution of $K_3[Co^{III}(CN)_5(N_3)]\cdot 2H_2O$ (1.50 g, 3.9 mmol, 1.0 eq.) in 7 mL of 57% hydroiodic acid and 4 mL of water, solid potassium nitrite (1.01 g, 11.9 mmol, 3.1 eq.) was added in the portion-wise manner. The mixture was vigorously stirred during the addition of reactants, and gases evolved. A color change from dark yellow to brownish red was observed. The reaction mixture was then heated on a hotplate to 40 °C and stirred for 20 min. After the solution cooled to room temperature, 40 mL of cold (–18 °C) ethanol was added, precipitating the brownish-red powder of $K_3[Co^{III}(CN)_5I]$. The product was vacuum-filtered and dried. During drying, it was noted that the product gradually gave off small amounts of iodine fumes. To prevent further decomposition, the product was stored in the fridge. Yield: 0.47 g (25.6%). In the air, together with the slow but gradual decomposition, this precursor absorbs water molecules. Elemental analysis. Calculated for the composition of the precursors after a few hours in the air (i.e., $K_3[Co^{III}(CN)_5I]\cdot 2H_2O$, molar mass: 469.26 g/mol): C: 12.8%, N: 14.9%, H: 0.9%. Experimentally found: C: 12.4%, N: 14.9%, H: 0.3%. The discrepancy in the elemental analysis (in the H-percentage) is due to the partial decomposition of the precursor upon staying in the air; however, when stored in the fridge under the dried

conditions, the precursor (freshly taken from the fridge) could be successfully used for the repeatable syntheses of compounds **5** and **6** (see below).

Synthesis of 1–6

In a small vial (7.5 mL volume), 2,2'-bipyridine-1,1'-dioxide (28.2 mg, 0.15 mmol, 1.5 eq.) was dissolved in 1.5 mL of distilled water and 1.5 mL of ethanol. To this ligand solution, a lanthanide salt was added as a solution of $Nd^{III}Cl_3·6H_2O$ (35.9 mg, 0.10 mmol, 1.0 eq.) in the case of **1**, **3,** and **5**, or $Ce^{III}Cl_3·7H_2O$ (37.3 mg, 0.10 mmol, 1.0 eq.) in case of **2**, **4** and **6**, in 1 mL of water. In samples containing Ce(III), yellow coloration was observed. To the resulting mixture, the solution of a cobalt(III)–cyanido precursor was added, i.e., $K_3[Co^{III}(CN)_5(N_3)]·2H_2O$ (38.4 mg, 0.10 mmol, 1.0 eq.) for **1** and **2**, $K_3[Co^{III}(CN)_5(NO_2)]$ (35.2 mg, 0.10 mmol, 1.0 eq.) for **3** and **4**, $K_3[Co^{III}(CN)_5I]$ (46.9 mg, 0.10 mmol, 1.0 eq.) for **5** and **6**, in 0.5 mL of distilled water. The vial was closed and left for crystallization for approximately 24 hours. Crystals of **1**–**6** were vacuum-filtered, then washed with ethanol and diethyl ether. Obtained crystals were characterized by elemental analysis and further by the SC-XRD analysis which provided the formulas of respective compounds. These results, including formulas and the related elemental analyses as well as the colors of crystals and yields, are summarized in Table S1. The phase purity of the obtained materials was checked by the powder X-ray diffraction (P-XRD) method (Figure S9), while the determination of the composition of air-dried samples was additionally supported by the TG studies (Figure S1).

It is worth commenting here that 1.5 equivalents of the organic ligand were used instead of 2 which was done to prevent the formation of an ionic salt with 4 organic ligands per each lanthanide(III) center as observed for hexacyanidocobaltate(III)-based similar molecular systems.[S4]

Table S1. Selected details of basic characterization of obtained bimetallic lanthanide(III)–pentacyanidocobaltate(III) compounds (L = 2,2'-bipyridine-1,1'-dioxide). The color of the column was selected to be different and characteristic of each compound; thus, it was later employed for the graphical presentation of other related figures and tables of the indicated compound.

**Compound**	**1**	**2**	**3**	**4**	**5**	**6**
**Formula** (taken from the SC-XRD analysis)	$\{[Nd^{III}(H_2O)_4L_2][Co^{III}(CN)_5(N_3)]\}·3H_2O$	$\{[Ce^{III}(H_2O)_4L_2][Co^{III}(CN)_5(N_3)]\}·3H_2O$	$\{[Nd^{III}(H_2O)_4L_2][Co^{III}(CN)_5(NO_2)]\}·3H_2O$	$\{[Ce^{III}(H_2O)_4L_2][Co^{III}(CN)_5(NO_2)]\}·3H_2O$	$\{[Nd^{III}(H_2O)_5L_2][Co^{III}(CN)_5I]\}·3H_2O$	$\{[Ce^{III}(H_2O)_5L_2][Co^{III}(CN)_5I]\}·3H_2O$
**Color of the sample**	dark yellow	dark yellow	pale pink	yellow	red	red
**Yield**	62.8 mg (72.3%)	56.0 mg (65.8%)	43.8 mg (49.7%)	59.1 mg (67.3%)	67.2 mg (69.2%)	64.8 mg (67.0%)
**Elemental analysis calculated from SC-XRD**	C: 34.21% N: 19.15% H: 3.44%	C: 34.37% N: 19.24% H: 3.46%	C: 34.05% N: 15.89% H: 3.43%	C: 34.21% N: 15.96% H: 3.45%	C: 30.62% N: 12.85% H: 3.29%	C: 30.75% N: 12.91% H: 3.30%
**Found** (exp.) **elemental analysis**[1]	C: 34.78% N: 19.72% H:3.54%	C: 34.93% N: 19.89% H: 3.65%	C: 34.02% N: 16.16% H: 3.58%	C: 34.72% N: 16.47% H: 3.64%	C: 31.23% N: 13.23% H: 3.33%	C: 31.54% N: 13.23% H: 3.30%

[1]The discrepancies occur between the experimental and calculated (based on the structural models from SC-XRD analyses) elemental analyses. In all such cases (**1**, **2**, **5**, and **6**), the experimental C- and N-percentages are higher than expected for the composition determined by the SC-XRD analyses. This suggests that the amount of water

molecules of crystallization is lower for the powder samples used for the CHN elemental analyses than those found in the SC-XRD analyses (which were performed by taking crystals directly from the mother solution, see below). This effect can be attributed to the gradual removal of the part of them upon long exposition to the air conditions which agrees with the results of TG (see Figure S1 below). These changes in the exact solvent content do not affect the significantly structural features as proven by the P-XRD data performed for the air-dried samples (Figure S9); however, to reliably perform structure-property correlations, the most sensitive magnetic measurements were executed on the relatively freshly-prepared samples covered by a protectant (see below).

Structural Studies

Single crystal X-ray diffraction (SC-XRD) analysis of all compounds was performed at 100(2) K with Bruker D8 Quest Eco Photo50 CMOS diffractometer, equipped with CRYOStream 800 Plus extension. Optical components included the molybdenum X-ray lamp (Mo Kα, 0.71073 Å), TRIUMPH monochromator, and CPAD Photon II detector. Single crystals of compounds **1**–**6** were taken directly from the mother solution, covered in APIEZON N grease, and mounted on the Micro Mounts™ holder which was inserted on the dedicated mounting of the diffractometer. The structures were solved by an intrinsic phasing method using a SHELXT program[S5] inside the Apex3 software. The crystal structures were refined in WinGx[S6] ver. 2021.3 by application of a weighted full-matrix least squares method on F^2. All non-hydrogen atoms were refined anisotropically, and hydrogen atoms' positions were calculated in their ideal position for aromatic hydrogen and refined using the riding model. In contrast, hydrogens of water molecules were found directly from electron density maps. Some DFIX-, ISOR-, and DELU-type restraints were applied to obtain proper geometry, keeping also the convergence of the refinement process.

The structural data for **1**–**6** was deposited in the CCDC database with the following numbers: 2331572, 2331573, 2331571, 2331576, 2331574, and 2331575, for **1**, **2**, **3**, **4**, **5**, and **6**, respectively. The representative parameters of crystal data and structure refinement are summarized in Tables S2 and S3, while the selected metric parameters of the crystal structures are shown in Tables S4–S9. Graphical presentation of the crystal structures of **1–6** is presented in Figures 1 and S3–S8. The related figures were prepared using Mercury 2022.2.0 software.

Powder X-ray diffraction (P-XRD) measurements were performed using a Bruker D8 Advance ECO diffractometer equipped with the copper X-ray lamp (Cu Kα, 1.5419 Å) and an SSD160 detector. Filtered and air-dried samples of **1**–**6** were ground and inserted in a glass capillary, which was later mounted in the diffractometer. All these measurements were performed at room temperature in the transmission mode using a rotating capillary system that prevents the appearance of a problem with the preferred orientation.

Physical Techniques

Measurements of physicochemical properties were performed on freshly prepared air-dried powder samples, with the additional protectant when necessary.

Elemental analyses were performed using the Elemental Vario Micro Cube CHN analyzer.

The infrared (IR) absorption spectra were collected on a Nicolet iN10 MX FT-IR microscope in a transmission mode, spanning the range of energies from 4000 to 675 cm^{-1} on transparent BaF_2 plates.

Thermogravimetric (TG) measurements were performed on a NETZSCH TG 209 F1 Libra apparatus under nitrogen flow of 20 mL·min^{-1} at a heating rate of 1 °C·min^{-1} in the temperature range of 20–440 °C, using an aluminum crucible as a sample holder.

Solid-state magnetic properties were measured on Quantum Design SQUID magnetometer MPMS®3, equipped with a cooling system EverCool®. Samples were prepared using the following procedure. Powdered or crushed crystals were placed at the bottom of the capsule, then submerged in paraffin oil (used to protect the sample from solvent removal as well as the orientation of the crystals under a strong magnetic field), and tightly secured in place by cotton. The capsule was closed with a lid filled with cotton. The resulting capsule was bonded with cotton thread into a straw holder and secured by Kapton tape.

Solid-state UV-vis-NIR absorption spectra were measured in the 220−1000 nm range on a Shimadzu UV-3600i plus spectrometer for powdered samples dispersed in paraffin oil and placed between quartz plates.

Solid-state photoluminescent properties were measured using an FS5 spectrofluorometer (Edinburgh Instruments) equipped with an Xe lamp (150 W) and thermoelectrically cooled InGaAs-1650 analog NIR detector with the lock-in-amplifier. Fluoracle software was used to record measurements. An SC-10 module equipped with a quartz cuvette as a sample holder was used for room-temperature measurements. A low-temperature setup employed an SC-70 module with q5000 quartz tubes submerged in liquid nitrogen.

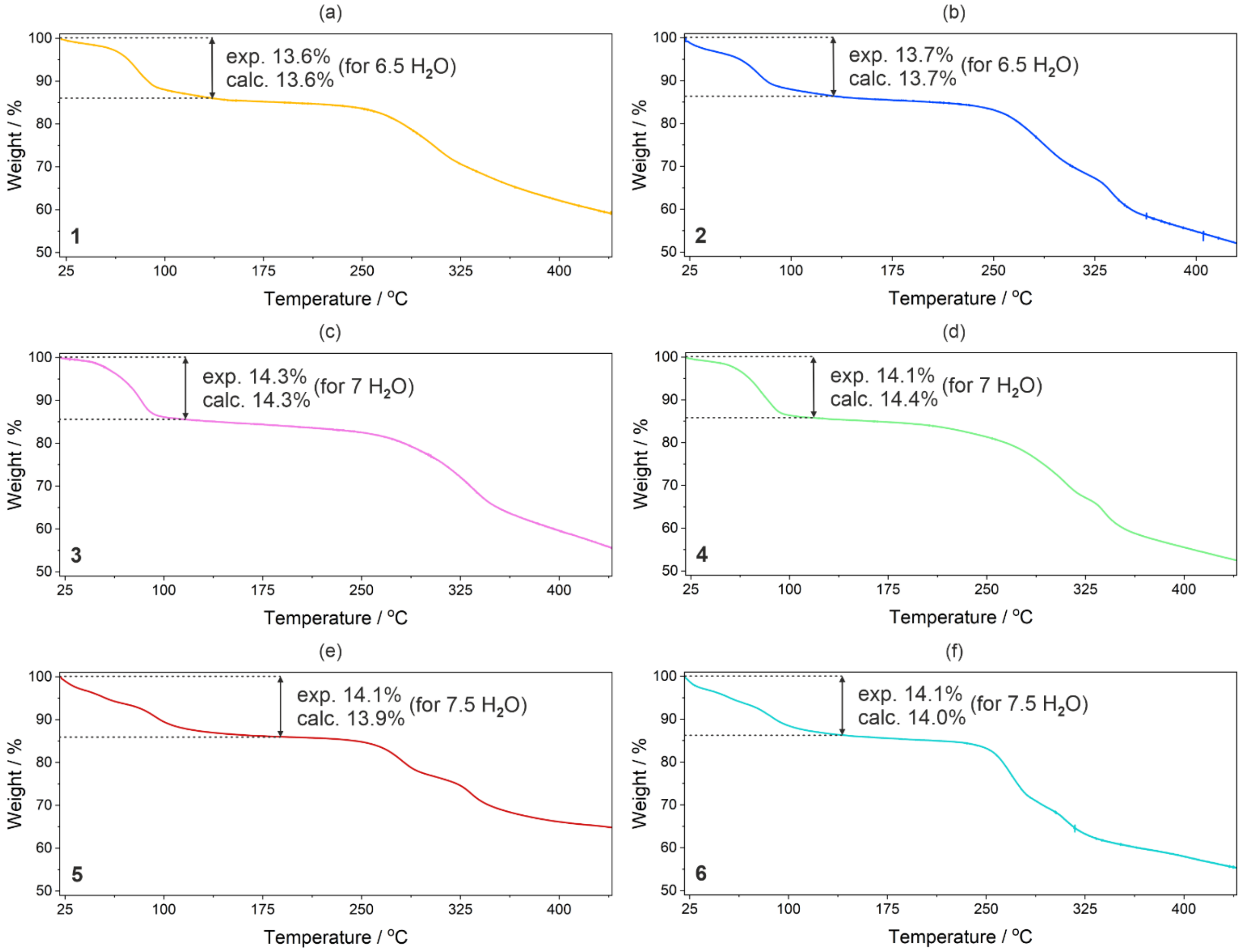

Figure S1. Thermogravimetric curves (TG) for crystalline samples of compounds **1**–**6** (a–f, respectively), collected in the 20–440 °C temperature range. Calculated and experimental steps in weight loss, related to water removal, are depicted.

Comment to Figure S1. TG measurements were performed for crystalline powdered samples, which were filtered and left in the air for a few hours. According to the elemental CNH, the air-stable phase loses a small amount of water during air-drying (see Table S1) when compared with the solvent content found in the respective SC-XRD analyses. This effect is particularly visible for compounds **1**, **2**, **5**, and **6**; and identical conclusions can be formulated from the presented TG curves. For **1** and **2**, the amount of water molecules per the formula unit is ca. 6.5 which is ca. 0.5 less than in the formula found in the structural model (Table S1). The identical situation is observed in **5** and **6**, where ca. 7.5 water molecules per the formula unit can be deduced from the TGA whereas 8 such molecules were observed in the SC-XRD analyses. These partial losses of water molecules do not, however, the crystal structures as found in the P-XRD data (Figure S9). This effect is not observed for **3** and **4** where the full set of solvent molecules seem to be very stable at room temperature, being gradually removed after heating at ca. 40 °C and above.

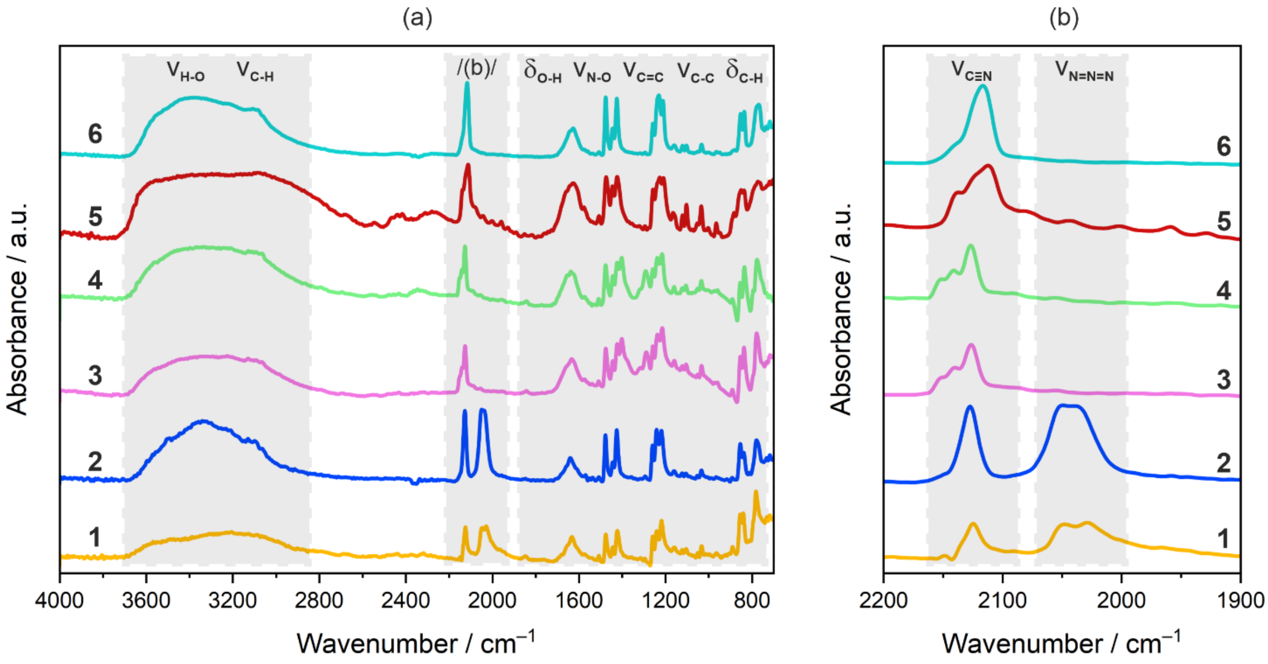

Figure S2. Infrared (IR) absorption spectra of selected crystals of compounds **1**–**6** in the broad range 4000–700 cm^{-1} (a) and the limited range 2200–1900 cm^{-1} (b), in which bands related to vibrations of cyanido and azido ligands are magnified.

Comment to Figure S2. IR absorption in the 4000−3000 cm^{-1} range is assigned to ν(O−H) of water molecules and ν(C−H) of the 2,2'-bipiridine-1,1'-dioxide ligands. The 1750–670 cm^{-1} range is composed of many characteristic absorption peaks, mostly of the ligand nature, such as δ(O−H) around 1700 cm^{-1}, ν(N–O) in the range of 1500–1400 cm^{-1}, ν(C=C) in the range of 1400 cm^{-1}, ν(C–C) placed in multiple parts of the fingerprint region, and aromatic δ(C−H) around 700 cm^{-1}. For the comparison of experimental spectra for 1 and 2 to the simulated ones with DFT methods, see Figure S41.

Table S2. Crystal data and structure refinement parameters for compounds **1**, **2,** and **3**.

Compound	1	2	3
Formula	$C_{25}H_{30}CoN_{12}NdO_{11}$	$C_{25}H_{30}CeCoN_{12}O_{11}$	$C_{25}H_{30}CoN_{10}NdO_{13}$
Formula weight / g·mol^{-1}	877.78	873.66	881.76
T / K	100(2)		
λ / Å	0.71073 (Mo Kα)		
Crystal system	monoclinic	monoclinic	monoclinic
Space group	$P\ 2_1/n$	$P\ 2_1/n$	$P\ 2_1/c$
a / Å	19.8776(12)	19.9479(14)	12.6884(19)
b / Å	7.8619(4)	7.8639(6)	7.9156(12)
c / Å	21.4270(11)	21.4450(13)	31.820(5)
α / °	90	90	90
β / °	102.630(2)	102.695(3)	90.430(5)
γ / °	90	90	90
V / Å^3	3267.5(3)	3281.8(4)	3195.8(8)
Z	4	4	4
ρ_{calc} / g·cm^{-1}	1.784	1.768	1.833
Crystal shape	plate	plate	plate
Crystal size / mm x mm x mm	0.100 x 0.080 x 0.030	0.040 x 0.030 x 0.003	0.050 x 0.040 x 0.040
Absorption coefficient / cm^{-1}	2.156	1.951	2.207
F(000)	1756	1748	1764
θ range / °	2.768–25.025	2.526–25.027	2.045–26.047
Limiting indices	$-17 < h < 23$<br>$-9 < k < 9$<br>$-25 < l < 22$	$-23 < h < 23$<br>$-9 < k < 9$<br>$-25 < l < 25$	$-15 < h < 15$<br>$-9 < k < 8$<br>$-39 < l < 39$
Collected reflections	11980	35517	33930
Unique reflections	5709	5678	6284
R_{int}	0.0519	0.0893	0.0860
Completeness / %	98.6	97.7	99.4
Data/restraints/parameters	5709/71/493	5678/53/493	6284/62/493
GOF on F^2	1.042	1.129	1.182
Final *R* indices<br>(R_1 for [$I > 2\sigma(I)$]<br>wR_2 for all data)	R_1=0.052<br>wR_2=0.1107	R_1=0.056<br>wR_2=0.0936	R_1=0.1861<br>wR_2=0.4252
Largest diff. peak and hole / e·Å^{-3}	1.205/–1.467	1.384/–1.896	9.813/–8.268

Table S3. Crystal data and structure refinement parameters for compounds **4**, **5,** and **6**.

Compound	4	5	6
Formula	$C_{25}H_{30}CeCoN_{10}O_{13}$	$C_{25}H_{32}CoIN_9NdO_{12}$	$C_{25}H_{32}CeCoIN_9O_{12}$
Formula weight / g·mol^{-1}	877.64	980.66	976.54
T / K	100(2)		
λ / Å	0.71073 (Mo Kα)		
Crystal system	monoclinic	monoclinic	monoclinic
Space group	$P\ 2_1/c$	$C\ 2/c$	$C\ 2/c$
a / Å	12.8271(4)	12.0633(9)	12.075(4)
b / Å	7.8484(2)	20.6436(16)	20.763(6)
c / Å	31.9184(10)	27.153(2)	27.189(9)
α / °	90	90	90
β / °	90.8240(10)	97.500(3)	97.471(9)
γ / °	90	90	90
V / Å^3	3212.96(16)	6704.1(9)	6759(4)
Z	4	8	8
ρ_{calc} / g·cm^{-1}	1.814	1.943	1.919
Crystal shape	block	block	block
Crystal size / mm x mm x mm	0.190 x 0.180 x 0.100	0.046 x 0.033 x 0.026	0.050 x 0.040 x 0.030
Absorption coefficient / cm^{-1}	1.996	3.021	2.807
F(000)	1756	3848	3832
θ range / °	2.553–26.391	1.968–25.023	1.962–26.021
Limiting indices	$-16 < h < 16$<br>$-9 < k < 9$<br>$-39 < l < 39$	$-14 < h < 13$<br>$-24 < k < 24$<br>$-32 < l < 32$	$-14 < h < 14$<br>$-25 < k < 25$<br>$-33 < l < 33$
Collected reflections	39810	41399	38751
Unique reflections	6573	5925	6663
R_{int}	0.0287	0.0998	0.1322
Completeness / %	99.8	99.9	99.9
Data/restraints/parameters	6573/29/493	5925/44/507	6663/51/507
GOF on F^2	1.363	1.095	1.092
Final R indices<br>(R_1 for [$I > 2\sigma(I)$]<br>wR_2 for all data)	R_1=0.0478<br>wR_2=0.1023	R_1=0.058<br>wR_2=0.1332	R_1=0.0955<br>wR_2=0.1984
Largest diff. peak and hole / e·Å^{-3}	1.753/–2.662	2.624/–1.581	2.972/–2.391

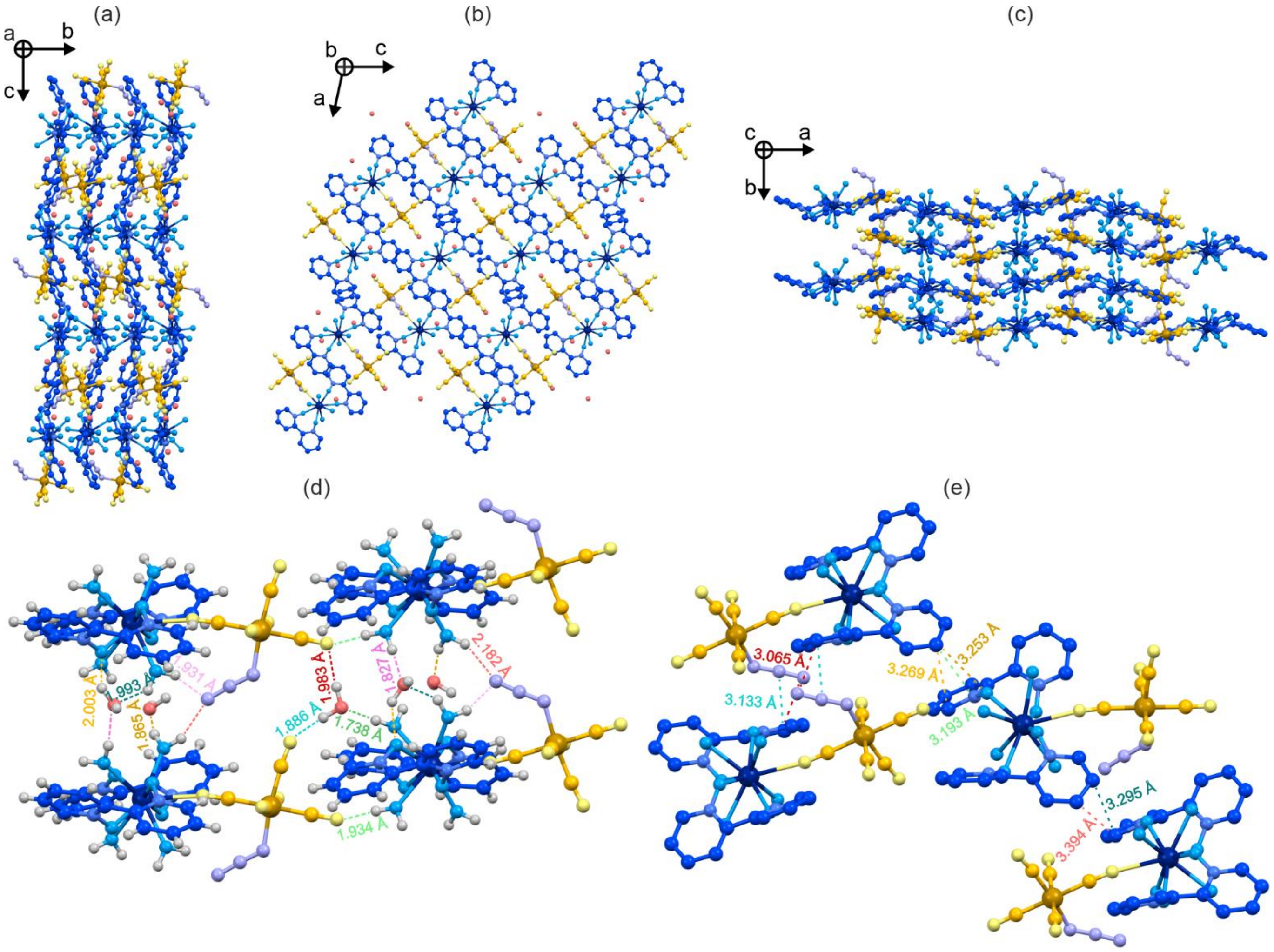

Figure S3. Additional structural views of **1** and **2**. Panels (a)–(c) present views of the crystal structures of **1** (selected to be presented in this figure as the representative of two isostructural compounds **1** and **2**) along the crystallographic axes *a*, *b*, and *c*, accordingly. In (d), hydrogen bonds controlling the supramolecular arrangement of dinuclear molecules were presented together with corresponding interatomic distances. The (e) panel visualizes other non-covalent interactions between molecules of the structure. Hydrogen atoms were omitted for clarity in panels (a)–(c) and (e). The color code of atoms was used as identical as shown in Figures 1 and S4 (see them for labeling scheme).

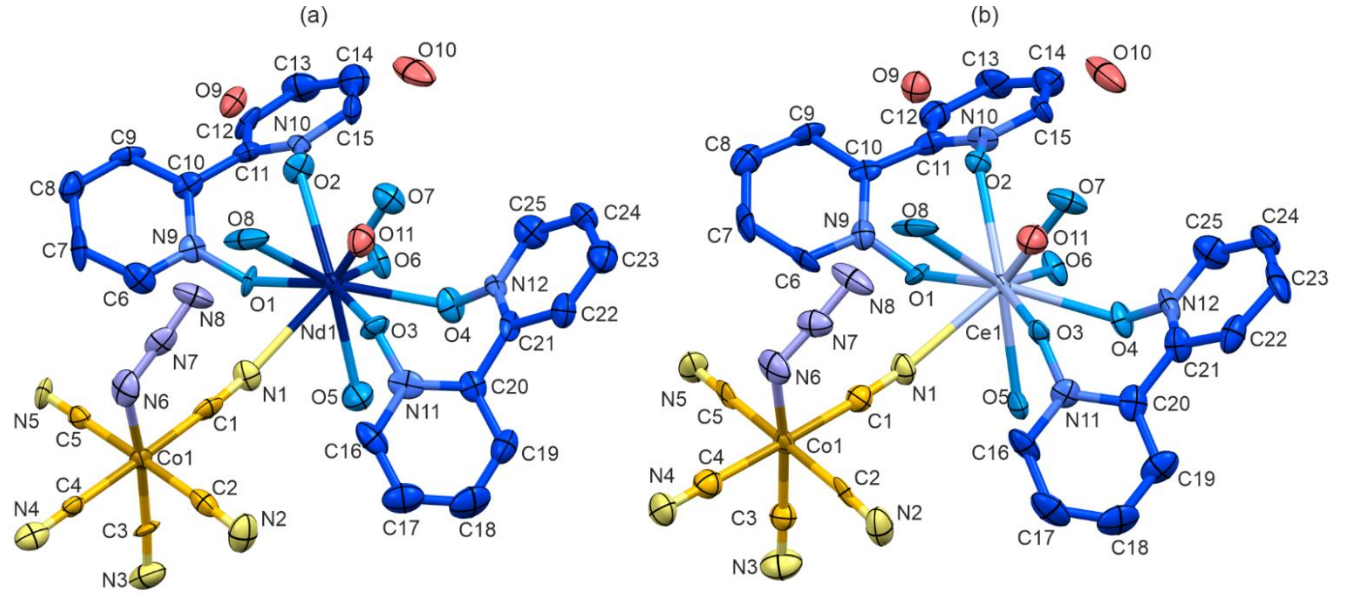

Figure S4. The views on the asymmetric units of **1** (a) and **2** (b). The atoms are presented with their thermal ellipsoids set at the 90% probability level. All non-hydrogen atoms of the structures are labeled. Hydrogen atoms were omitted for the sake of clarity.

Table S4. Selected bond lengths and angles in the crystal structure of compound **1**.

Bond lengths of the first coordination sphere of metal centers in {[$Nd^{III}(H_2O)_4$(2,2'-bipyridine-1,1'-dioxide)$_2$][$Co^{III}(CN)_5(N_3)$]}·$3H_2O$ (compound 1) / Å					
**Nd1–O1**	2.404(4)	**Nd1–O5**	2.515(5)	**Co1–C4**	1.896(7)
**Nd1–O3**	2.445(5)	**Nd1–O2**	2.518(5)	**Co1–C1**	1.902(7)
**Nd1–O4**	2.452(5)	**Nd1–O7**	2.522(5)	**Co1–C2**	1.906(8)
**Nd1–O8**	2.480(5)	**Nd1–N1**	2.675(6)	**Co1–C5**	1.916(7)
**Nd1–O6**	2.497(5)	**Co1–C3**	1.850(7)	**Co1–N6**	1.992(6)
**Angles between bonds of the first coordination sphere of metal centers in {[$Nd^{III}(H_2O)_4$(2,2'-bipyridine-1,1'-dioxide)$_2$][$Co^{III}(CN)_5(N_3)$]}·$3H_2O$ (compound 1) / °**					
**O1–Nd1–O3**	149.94(16)	**O8–Nd1–O2**	115.02(16)	**O7–Nd1–N1**	122.87(17)
**O1–Nd1–O4**	131.28(16)	**O6–Nd1–O2**	66.62(16)	**C3–Co1–C4**	127.93(17)
**O3–Nd1–O4**	68.72(15)	**O5–Nd1–O2**	74.17(16)	**C3–Co1–C1**	85.6(3)
**O1–Nd1–O8**	85.47(18)	**O1–Nd1–O7**	145.73(16)	**C4–Co1–C1**	94.9(3)
**O3–Nd1–O8**	81.89(17)	**O3–Nd1–O7**	135.01(16)	**C3–Co1–C2**	178.8(3)
**O4–Nd1–O8**	142.42(18)	**O4–Nd1–O7**	66.66(15)	**C4–Co1–C2**	86.5(3)
**O1–Nd1–O6**	68.05(15)	**O8–Nd1–O7**	72.87(16)	**C1–Co1–C2**	90.3(3)
**O3–Nd1–O6**	135.53(16)	**O6–Nd1–O7**	74.27(18)	**C3–Co1–C5**	90.8(3)
**O4–Nd1–O6**	66.90(16)	**O5–Nd1–O7**	102.18(15)	**C4–Co1–C5**	89.5(3)
**O8–Nd1–O6**	138.76(17)	**O2–Nd1–O7**	137.30(17)	**C1–Co1–C5**	91.2(3)
**O1–Nd1–O5**	84.93(16)	**O1–Nd1–N1**	67.06(16)	**C2–Co1–C5**	87.7(3)
**O3–Nd1–O5**	84.47(16)	**O3–Nd1–N1**	76.09(16)	**C3–Co1–N6**	175.7(3)
**O4–Nd1–O5**	67.44(16)	**O4–Nd1–N1**	73.88(16)	**C4–Co1–N6**	173.4(3)
**O8–Nd1–O5**	133.98(16)	**O8–Nd1–N1**	122.07(17)	**C1–Co1–N6**	87.8(3)
**O6–Nd1–O5**	76.41(17)	**O6–Nd1–N1**	67.62(18)	**C2–Co1–N6**	91.7(3)
**O1–Nd1–O2**	68.06(15)	**O5–Nd1–N1**	129.87(17)	**C5–Co1–N6**	93.8(3)
**O3–Nd1–O2**	129.28(16)	**O2–Nd1–N1**	66.38(17)	**O7–Nd1–N1**	90.3(3)

Table S5. Selected bond lengths and angles in the crystal structure of compound **2**.

Bond lengths of the first coordination sphere of metal centers in {[Ce^{III}(H_2O)$_4$(2,2'-bipyridine-1,1'-dioxide)$_2$][Co^{III}(CN)$_5$(N_3)]}·3H_2O (compound 2) / Å					
**Ce1–O1**	2.428(4)	**Ce1–O2**	2.544(4)	**Co1–C1**	1.907(7)
**Ce1–O4**	2.479(4)	**Ce1–O5**	2.547(5)	**Co1–C4**	1.908(7)
**Ce1–O3**	2.480(4)	**Ce1–O7**	2.551(5)	**Co1–C2**	1.915(7)
**Ce1–O6**	2.518(5)	**Ce1–N1**	2.680(5)	**Co1–C5**	1.923(7)
**Ce1–O8**	2.520(5)	**Co1–C3**	1.858(7)	**Co1–N6**	1.993(6)
**Angles between bonds of the first coordination sphere of metal centers in {[Ce^{III}(H_2O)$_4$(2,2'-bipyridine-1,1'-dioxide)$_2$][Co^{III}(CN)$_5$(N_3)]}·3H_2O (compound 2) / °**					
**O1–Ce1–O4**	132.01(15)	**O3–Ce1–O5**	83.98(16)	**O2–Ce1–N1**	66.49(17)
**O1–Ce1–O3**	150.07(14)	**O6–Ce1–O5**	76.65(16)	**O5–Ce1–N1**	127.81(17)
**O4–Ce1–O3**	68.11(14)	**O8–Ce1–O5**	134.06(16)	**O7–Ce1–N1**	95.7(3)
**O1–Ce1–O6**	68.13(15)	**O2–Ce1–O5**	146.19(16)	**C3–Co1–C1**	84.3(3)
**O4–Ce1–O6**	67.50(15)	**O1–Ce1–O7**	134.76(15)	**C3–Co1–C4**	178.7(3)
**O3–Ce1–O6**	135.53(15)	**O4–Ce1–O7**	72.89(15)	**C1–Co1–C4**	87.0(3)
**O1–Ce1–O8**	84.86(17)	**O3–Ce1–O7**	66.43(15)	**C3–Co1–C2**	90.1(3)
**O4–Ce1–O8**	142.33(17)	**O6–Ce1–O7**	102.51(15)	**C1–Co1–C2**	91.2(3)
**O3–Ce1–O8**	82.24(16)	**O8–Ce1–O7**	74.23(17)	**C4–Co1–C2**	89.4(3)
**O6–Ce1–O8**	138.46(16)	**O2–Ce1–O7**	67.52(15)	**C3–Co1–C5**	88.0(3)
**O1–Ce1–O2**	67.37(14)	**O5–Ce1–O7**	136.83(16)	**C1–Co1–C5**	90.7(3)
**O4–Ce1–O2**	115.96(14)	**O1–Ce1–N1**	75.94(15)	**C4–Co1–C5**	175.8(3)
**O3–Ce1–O2**	129.38(15)	**O4–Ce1–N1**	121.88(16)	**C2–Co1–C5**	172.7(3)
**O6–Ce1–O2**	74.46(15)	**O3–Ce1–N1**	74.18(15)	**C3–Co1–N6**	91.6(3)
**O8–Ce1–O2**	66.02(15)	**O6–Ce1–N1**	129.67(17)	**C1–Co1–N6**	88.4(3)
**O1–Ce1–O5**	85.84(16)	**O8–Ce1–N1**	67.60(17)	**C4–Co1–N6**	93.5(3)
**O4–Ce1–O5**	67.02(15)	**O3–Ce1–O5**	122.13(16)	**C2–Co1–N6**	90.3(3)

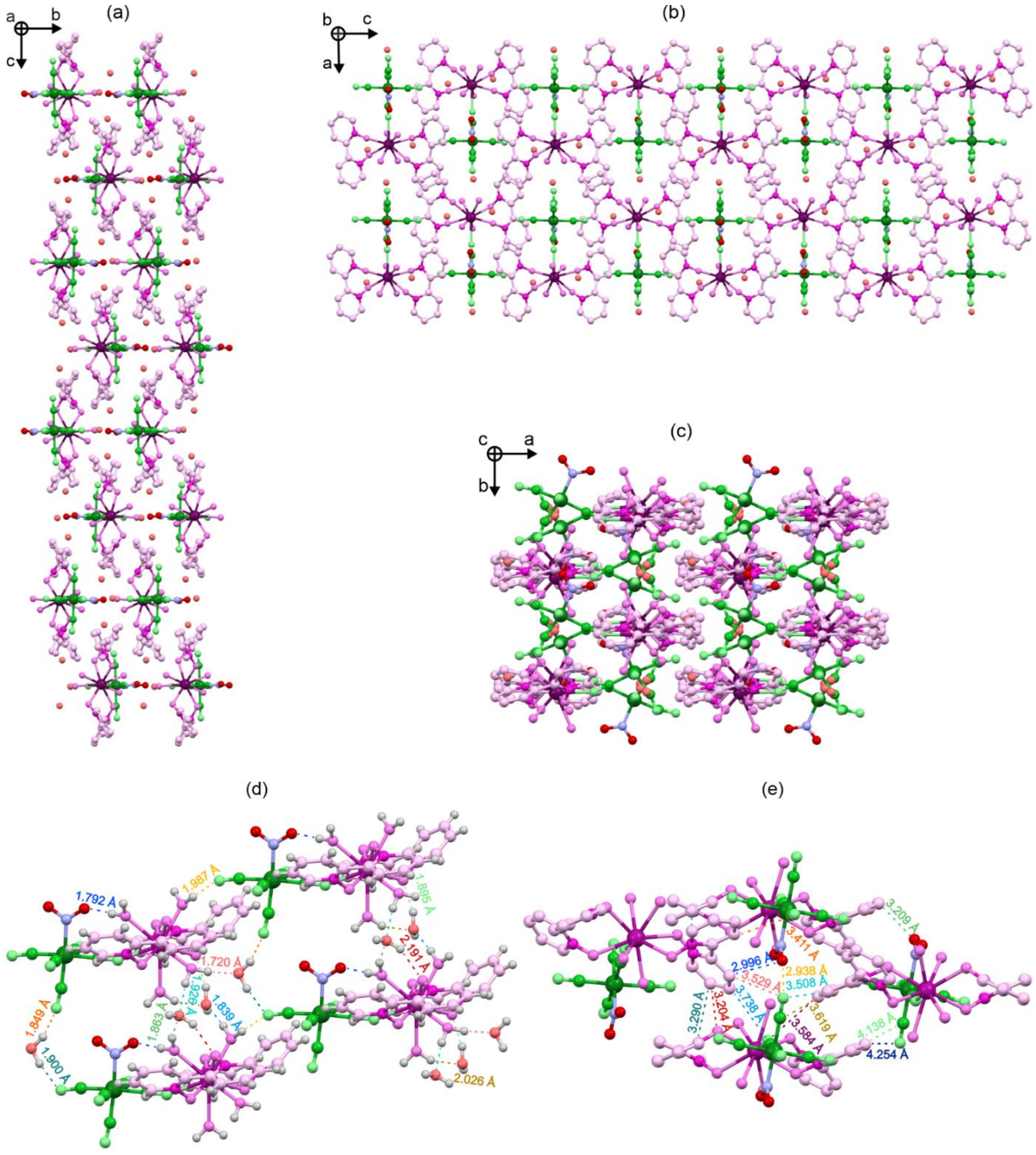

Figure S5. Additional structural views of **3** and **4**. Panels (a)–(c) present views of the crystal structures of **4** (selected to be presented in this figure as the representative of two isostructural compounds **3** and **4**, especially that comes from a better quality SC-XRD data) along the crystallographic axes *a*, *b*, and *c*, accordingly. In (d), hydrogen bonds controlling the supramolecular arrangement of dinuclear molecules were presented together with corresponding interatomic distances. The (e) panel visualizes other non-covalent interactions between molecules of the structure. Hydrogen atoms were omitted for clarity in panels (a)–(c) and (e). The color code of atoms was used as identical as shown in Figures 1 and S6 (see them for labeling scheme).

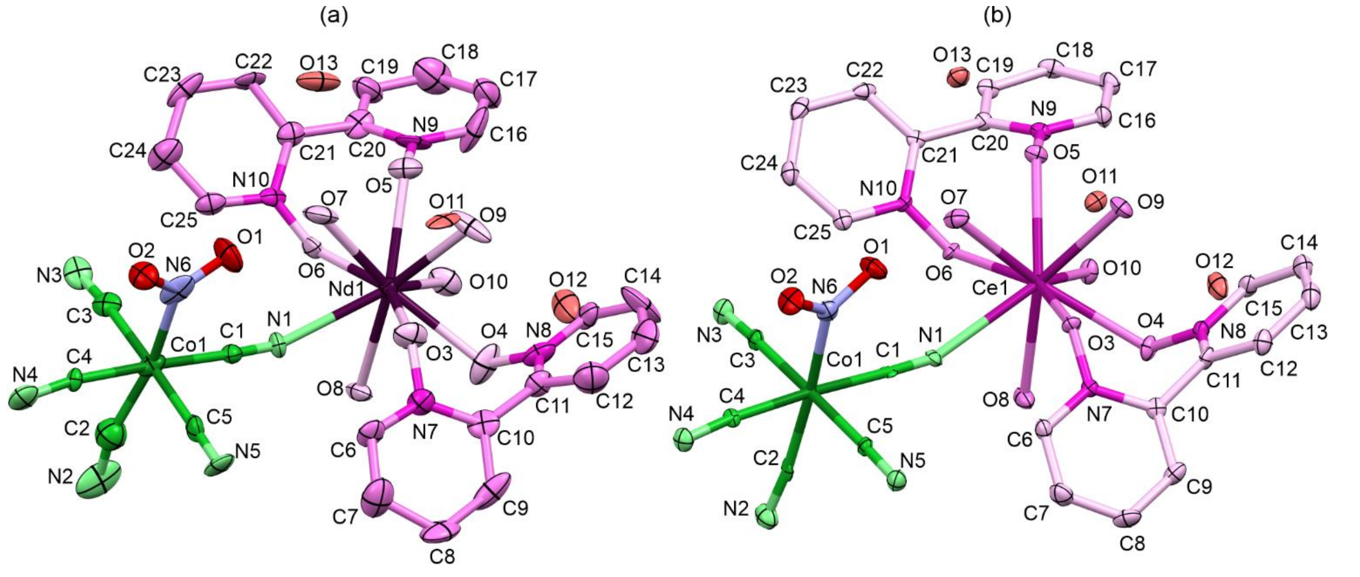

Figure S6. The views on the asymmetric units of **3** (a) and **4** (b). The atoms are presented with their thermal ellipsoids set at the 50% probability level. All non-hydrogen atoms of the structures are labeled. Hydrogen atoms were omitted for the sake of clarity.

Table S6. Selected bond lengths and angles in the crystal structure of compound **3**.

Bond lengths of the first coordination sphere of metal centers in {[$Nd^{III}(H_2O)_4$(2,2'-bipyridine-1,1'-dioxide)$_2$][$Co^{III}(CN)_5(NO_2)$]}·3H_2O (compound 3) / Å					
**Nd1–O6**	2.39(2)	**Nd1–O3**	2.48(2)	**Co1–C2**	1.850(18)
**Nd1–O7**	2.45(2)	**Nd1–O10**	2.53(2)	**Co1–C3**	1.90(3)
**Nd1–O4**	2.45(2)	**Nd1–N1**	2.61(2)	**Co1–C5**	1.92(3)
**Nd1–O5**	2.477(19)	**Nd1–O8**	2.684(19)	**Co1–C1**	1.94(3)
**Nd1–O9**	2.48(2)	**Co1–C4**	1.82(2)	**Co1–N6**	1.99(3)
**Angles between bonds of the first coordination sphere of metal centers in {[$Nd^{III}(H_2O)_4$(2,2'-bipyridine-1,1'-dioxide)$_2$][$Co^{III}(CN)_5(NO_2)$]}·3H_2O (compound 3) / °**					
**O6–Nd1–O7**	79.7(8)	**O4–Nd1–O10**	69.8(7)	**O10–Nd1–O8**	67.4(6)
**O6–Nd1–O4**	134.0(7)	**O5–Nd1–O10**	72.5(7)	**N1–Nd1–O8**	67.9(7)
**O7–Nd1–O4**	146.3(8)	**O9–Nd1–O10**	100.5(7)	**C4–Co1–C2**	75.8(13)
**O6–Nd1–O5**	67.1(7)	**O3–Nd1–O10**	139.3(8)	**C4–Co1–C3**	90.2(11)
**O7–Nd1–O5**	67.2(7)	**O6–Nd1–N1**	77.0(7)	**C2–Co1–C3**	88.4(14)
**O4–Nd1–O5**	119.3(8)	**O7–Nd1–N1**	68.2(8)	**C4–Co1–C5**	88.7(11)
**O6–Nd1–O9**	129.8(8)	**O4–Nd1–N1**	114.4(8)	**C2–Co1–C5**	90.0(14)
**O7–Nd1–O9**	75.6(9)	**O5–Nd1–N1**	126.3(7)	**C3–Co1–C5**	178.2(13)
**O4–Nd1–O9**	79.0(9)	**O9–Nd1–N1**	129.4(7)	**C4–Co1–C1**	176.2(11)
**O5–Nd1–O9**	63.2(8)	**O3–Nd1–N1**	74.9(7)	**C2–Co1–C1**	100.4(13)
**O6–Nd1–O3**	149.4(7)	**O10–Nd1–N1**	130.0(8)	**C3–Co1–C1**	89.2(11)
**O7–Nd1–O3**	78.8(8)	**O6–Nd1–O8**	73.5(7)	**C5–Co1–C1**	91.8(11)
**O4–Nd1–O3**	70.3(7)	**O7–Nd1–O8**	132.4(7)	**C4–Co1–N6**	92.6(11)
**O5–Nd1–O3**	122.5(7)	**O4–Nd1–O8**	71.1(8)	**C2–Co1–N6**	168.2(13)
**O9–Nd1–O3**	64.2(7)	**O5–Nd1–O8**	131.0(7)	**C3–Co1–N6**	89.8(11)
**O6–Nd1–O10**	70.0(7)	**O9–Nd1–O8**	150.1(8)	**C5–Co1–N6**	91.7(11)
**O7–Nd1–O10**	136.6(7)	**O3–Nd1–O8**	106.3(7)	**C1–Co1–N6**	91.2(11)

Table S7. Selected bond lengths and angles in the crystal structure of compound **4**.

Bond lengths of the first coordination sphere of metal centers in {[$Ce^{III}(H_2O)_4$(2,2'-bipyridine-1,1'-dioxide)$_2$][$Co^{III}(CN)_5(NO_2)$]}·$3H_2O$ (compound 4) / Å					
**Ce1–O6**	2.445(4)	**Ce1–O9**	2.549(4)	**Co1–C4**	1.898(5)
**Ce1–O3**	2.484(4)	**Ce1–O5**	2.576(4)	**Co1–C1**	1.899(5)
**Ce1–O4**	2.511(4)	**Ce1–O8**	2.643(4)	**Co1–C3**	1.907(5)
**Ce1–O10**	2.519(4)	**Ce1–N1**	2.664(4)	**Co1–C5**	1.913(5)
**Ce1–O7**	2.525(4)	**Co1–C2**	1.879(6)	**Co1–N6**	1.970(5)
**Angles between bonds of the first coordination sphere of metal centers in {[$Ce^{III}(H_2O)_4$(2,2'-bipyridine-1,1'-dioxide)$_2$][$Co^{III}(CN)_5(NO_2)$]}·$3H_2O$ (compound 4) / °**					
**O6–Ce1–O3**	150.92(12)	**O4–Ce1–O5**	120.26(12)	**O5–Ce1–N1**	126.90(13)
**O6–Ce1–O4**	134.62(12)	**O10–Ce1–O5**	73.49(12)	**O8–Ce1–N1**	66.77(13)
**O3–Ce1–O4**	68.85(12)	**O7–Ce1–O5**	68.88(12)	**C2–Co1–C4**	82.6(2)
**O6–Ce1–O10**	68.77(12)	**O9–Ce1–O5**	64.07(12)	**C2–Co1–C1**	93.0(2)
**O3–Ce1–O10**	139.09(12)	**O6–Ce1–O8**	78.48(13)	**C4–Co1–C1**	175.6(2)
**O4–Ce1–O10**	70.92(12)	**O3–Ce1–O8**	101.72(13)	**C2–Co1–C3**	87.7(2)
**O6–Ce1–O7**	80.35(13)	**O4–Ce1–O8**	68.58(13)	**C4–Co1–C3**	91.1(2)
**O3–Ce1–O7**	78.01(13)	**O10–Ce1–O8**	69.61(13)	**C1–Co1–C3**	88.8(2)
**O4–Ce1–O7**	145.02(13)	**O7–Ce1–O8**	131.21(13)	**C2–Co1–C5**	90.7(2)
**O10–Ce1–O7**	138.53(13)	**O9–Ce1–O8**	145.06(13)	**C4–Co1–C5**	89.1(2)
**O6–Ce1–O9**	129.95(12)	**O5–Ce1–O8**	135.83(12)	**C1–Co1–C5**	90.8(2)
**O3–Ce1–O9**	64.11(12)	**O6–Ce1–N1**	79.54(12)	**C3–Co1–C5**	178.4(2)
**O4–Ce1–O9**	76.48(13)	**O3–Ce1–N1**	74.13(13)	**C2–Co1–N6**	175.7(2)
**O10–Ce1–O9**	99.53(13)	**O4–Ce1–N1**	112.76(13)	**C4–Co1–N6**	93.3(2)
**O7–Ce1–O9**	79.33(13)	**O10–Ce1–N1**	129.97(13)	**C1–Co1–N6**	91.1(2)
**O6–Ce1–O5**	65.98(12)	**O7–Ce1–N1**	66.42(13)	**C3–Co1–N6**	91.2(2)
**O3–Ce1–O5**	122.14(12)	**O9–Ce1–N1**	130.37(13)	**C5–Co1–N6**	90.4(2)

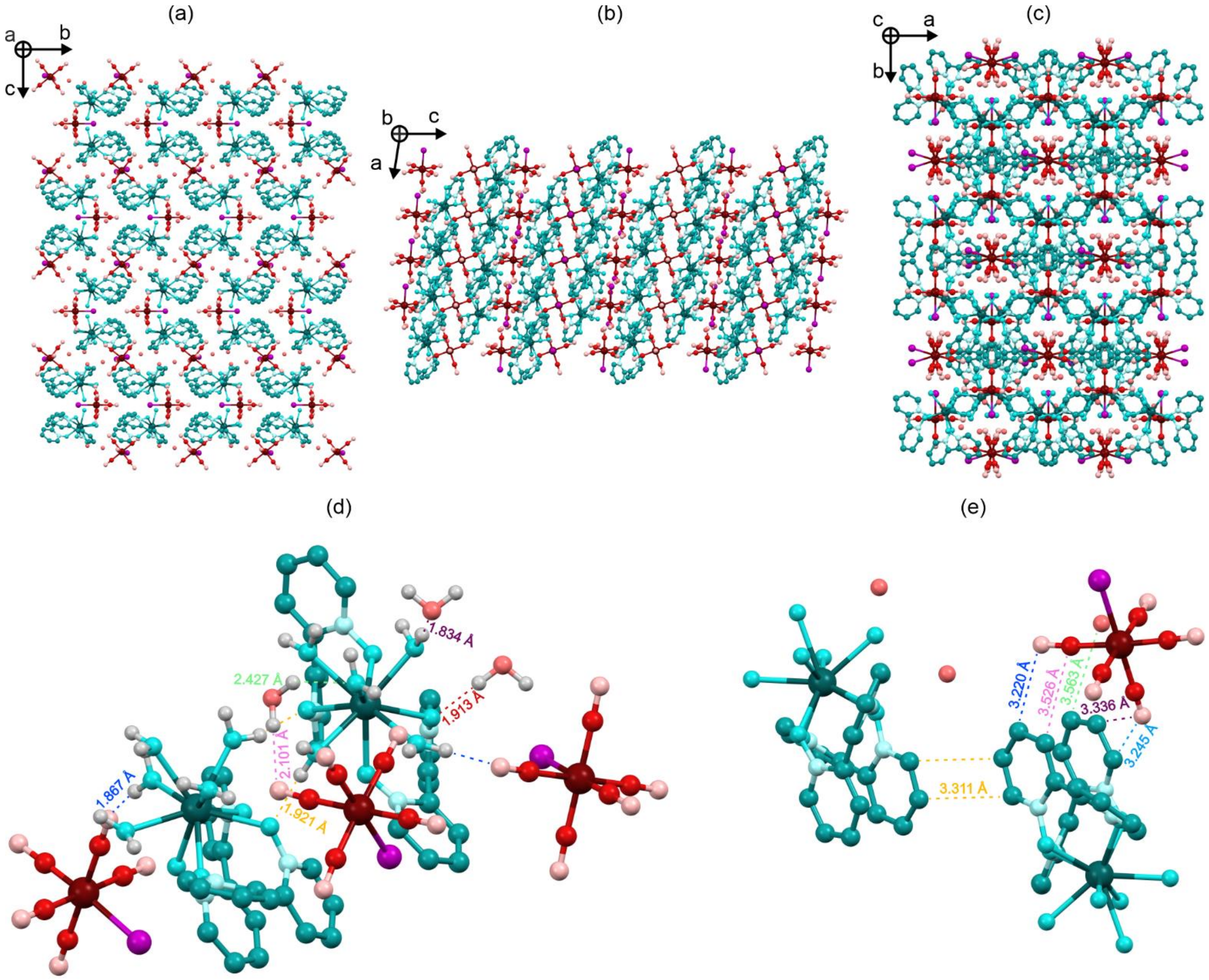

Figure S7. Additional structural views of **5** and **6**. Panels (a)–(c) present views of the crystal structures of **5** (selected to be presented in this figure as the representative of two isostructural compounds **5** and **6**) along the crystallographic axes *a*, *b*, and *c*, accordingly. In (d), hydrogen bonds controlling the supramolecular arrangement of metal complexes were presented together with corresponding interatomic distances. The (e) panel visualizes other non-covalent interactions between molecules of the structure. Hydrogen atoms were omitted for clarity in panels (a)–(c) and (e). The color code of atoms was used as identical as shown in Figures 1 and S8 (see them for labeling scheme).

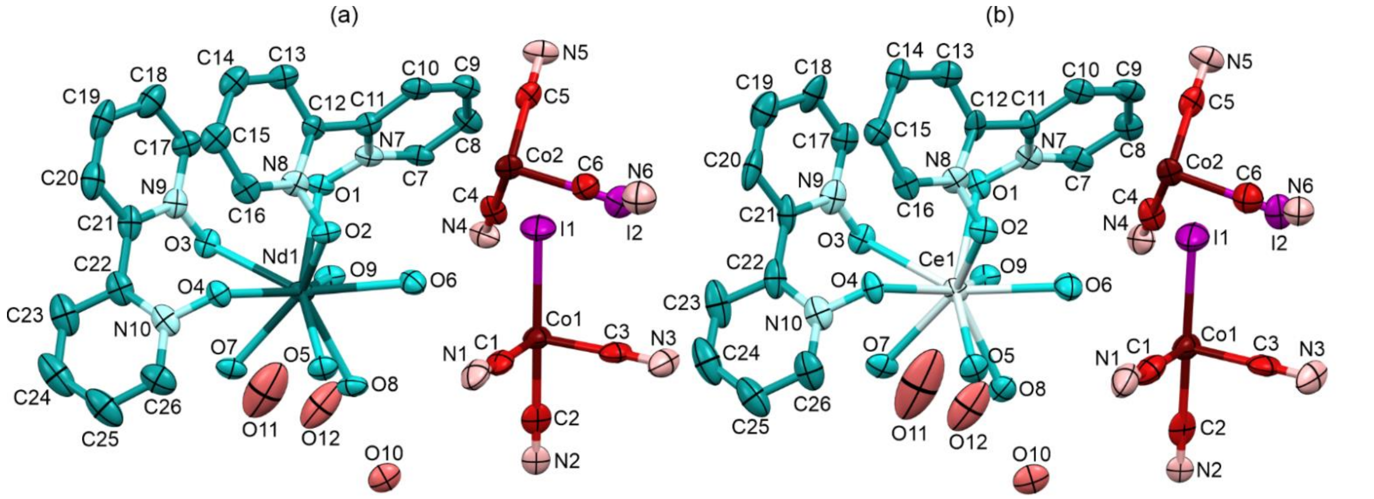

Figure S8. The views on the asymmetric units of **5** (a) and **6** (b). The atoms are presented with their thermal ellipsoids set at the 50% probability level. All non-hydrogen atoms of the structures are labeled. Hydrogen atoms were omitted for the sake of clarity.

Table S8. Selected bond lengths and angles in the crystal structure of compound **5**.

Bond lengths of the first coordination sphere of metal centers in {[Nd^{III}(H_2O)$_5$(2,2'-bipyridine-1,1'-dioxide)$_2$][Co^{III}(CN)$_5$I]}·3H_2O (compound 5) / Å					
**Nd1–O4**	2.434(6)	**Nd1–O3**	2.508(6)	**Co1–N2**	1.929(18)
**Nd1–O1**	2.442(5)	**Nd1–O7**	2.512(6)	**Co2–C4**	1.933(11)
**Nd1–O5**	2.471(6)	**Nd1–O8**	2.579(6)	**Co2–I2**	2.595(2)
**Nd1–O6**	2.486(6)	**Co1I1**	2.6102(19)	**Co2–C6**	1.753(19)
**Nd1–O2**	2.493(5)	**Co1–C1**	1.890(12)	**Co2–C5**	1.897(9)
**Nd1–O9**	2.503(6)	**Co1–C3**	1.909(11)		
**Angles between bonds of the first coordination sphere of metal centers in {[Nd^{III}(H_2O)$_5$(2,2'-bipyridine-1,1'-dioxide)$_2$][Co^{III}(CN)$_5$I]}·3H_2O (compound 5) / °**					
**O4–Nd1–O1**	105.8(2)	**O1–Nd1–O3**	70.2(2)	**O2–Nd1–O8**	130.1(2)
**O4–Nd1–O5**	70.8(2)	**O5–Nd1–O3**	134.5(2)	**O9–Nd1–O8**	67.98(19)
**O1–Nd1–O5**	139.9(2)	**O6–Nd1–O3**	145.4(2)	**O3–Nd1–O8**	125.0(2)
**O4–Nd1–O6**	133.8(2)	**O2–Nd1–O3**	104.73(18)	**O7–Nd1–O8**	64.4(2)
**O1–Nd1–O6**	76.7(2)	**O9–Nd1–O3**	79.19(19)	**C6–Co2–C5**	89.0(7)
**O5–Nd1–O6**	78.6(2)	**O4–Nd1–O7**	77.2(2)	**C6–Co2–C4**	92.6(8)
**O4–Nd1–O2**	68.9(2)	**O1–Nd1–O7**	133.09(19)	**C5–Co2–C4**	90.9(4)
**O1–Nd1–O2**	67.22(18)	**O5–Nd1–O7**	86.2(2)	**C5–Co2–I2**	89.3(3)
**O5–Nd1–O2**	75.0(2)	**O6–Nd1–O7**	134.9(2)	**C4–Co2–I2**	87.3(3)
**O6–Nd1–O2**	70.3(2)	**O2–Nd1–O7**	145.0(2)	**C1–Co1–C3**	91.6(4)
**O4–Nd1–O9**	145.1(2)	**O9–Nd1–O7**	81.9(2)	**C1–Co1–C2**	91.7(3)
**O1–Nd1–O9**	69.59(19)	**O3–Nd1–O7**	68.4(2)	**C3–Co1–C2**	89.1(3)
**O5–Nd1–O9**	135.5(2)	**O4–Nd1–O8**	124.5(2)	**C1–Co1–I1**	88.3(3)
**O6–Nd1–O9**	80.1(2)	**O1–Nd1–O8**	129.65(19)	**C3–Co1–I1**	90.9(3)
**O2–Nd1–O9**	131.8(2)	**O5–Nd1–O8**	68.2(2)	**C2–Co1–I1**	180
**O4–Nd1–O3**	67.21(19)	**O6–Nd1–O8**	70.5(2)		

Table S9. Selected bond lengths and angles in the crystal structure of compound **6**.

Bond lengths of the first coordination sphere of metal centers in {[$Ce^{III}(H_2O)_5$(2,2'-bipyridine-1,1'-dioxide)$_2$][$Co^{III}(CN)_5I$]}·$3H_2O$ (compound 6) / Å					
**Ce1–O4**	2.466(9)	**Ce1–O7**	2.540(10)	**Co1–C2**	1.95(3)
**Ce1–O1**	2.472(9)	**Ce1–O9**	2.546(9)	**Co2–C6**	1.83(2)
**Ce1–O5**	2.513(9)	**Ce1–O8**	2.632(10)	**Co2–C5**	1.913(15)
**Ce1–O6**	2.519(10)	**Co1–I1**	2.620(3)	**Co2–C4**	1.931(16)
**Ce1–O2**	2.532(9)	**Co1–C1**	1.90(2)	**Co2–I2**	2.600(3)
**Ce1–O3**	2.537(9)	**Co1–C3**	1.914(18)		
**Angles between bonds of the first coordination sphere of metal centers in {[$Ce^{III}(H_2O)_5$(2,2'-bipyridine-1,1'-dioxide)$_2$][$Co^{III}(CN)_5I$]}·$3H_2O$ (compound 6) / °**					
**O4–Ce1–O1**	105.2(3)	**O1–Ce1–O7**	133.8(3)	**O2–Ce1–O8**	131.1(3)
**O4–Ce1–O5**	70.9(3)	**O5–Ce1–O7**	85.1(3)	**O3–Ce1–O8**	124.8(3)
**O1–Ce1–O5**	140.2(3)	**O6–Ce1–O7**	134.1(3)	**O7–Ce1–O8**	63.8(3)
**O4–Ce1–O6**	133.9(3)	**O2–Ce1–O7**	144.9(3)	**O9–Ce1–O8**	67.7(3)
**O1–Ce1–O6**	77.3(3)	**O3–Ce1–O7**	68.7(3)	**C6–Co2–C5**	90.0(12)
**O5–Ce1–O6**	78.7(3)	**O4–Ce1–O9**	145.6(3)	**C6–Co2–C4**	91.2(13)
**O4–Ce1–O2**	68.6(3)	**O1–Ce1–O9**	69.3(3)	**C5–Co2–C4**	90.4(6)
**O1–Ce1–O2**	66.6(3)	**O5–Ce1–O9**	135.5(3)	**C5–Co2–I2**	89.5(4)
**O5–Ce1–O2**	75.7(3)	**O6–Ce1–O9**	79.4(3)	**C4–Co2–I2**	87.4(5)
**O6–Ce1–O2**	70.8(3)	**O2–Ce1–O9**	130.8(3)	**C1–Co1–C3**	90.7(7)
**O4–Ce1–O3**	66.1(3)	**O3–Ce1–O9**	80.6(3)	**C1–Co1–C2**	91.8(5)
**O1–Ce1–O3**	70.7(3)	**O7–Ce1–O9**	83.0(3)	**C3–Co1–C2**	88.6(4)
**O5–Ce1–O3**	133.2(3)	**O4–Ce1–O8**	125.0(3)	**C1–Co1–I1**	88.2(5)
**O6–Ce1–O3**	146.6(3)	**O1–Ce1–O8**	129.8(3)	**C3–Co1–I1**	91.4(4)
**O2–Ce1–O3**	103.9(3)	**O5–Ce1–O8**	68.5(3)	**C2–Co1–I1**	180
**O4–Ce1–O7**	77.5(3)	**O6–Ce1–O8**	70.3(3)		

Comment to Figures S3–S8 and Tables S4–S9 – detailed structural data of 1–6 and its implications to the expected magnetic anisotropy of embedded lanthanide(III) centers

More precise structural views (than those presented in the main text in Figure 1) of **1**–**6**, which include hydrogen bonds between all molecular components and π-stacking interactions between the ligands stabilizing the 3-D supramolecular networks, can be found in Figures S3–S8. These supporting figures provide also better insight into the details of coordination spheres of lanthanide(III) centers. Since the oblate electron density can characterize both Nd(III) and Ce(III) magnetic centers, we are particularly interested in generating an axial ligand field that would stabilize m_J states of the ground multiplet with the highest possible magnetic momenta. Our initial assumption is that the arrangement of electron-rich donor oxygen atoms of 2,2'-bpdo ligands with partial negative charges will eventually be a decisive factor for the axiality of the generated crystal field. Thus, investigating some interesting bond lengths and angles is instructive at this stage (they are given in Tables S4–S9). As the former is concerned, we can only observe that in **1**–**4**, Ln–N bonds corresponding to the interactions with metalloligands are longer, over 2.60 Å, and weaker than Ln–O bonds, ranging from 2.42 to 2.58 Å. At the same time, there is no clear distinction or trend along the series of compounds and between Ln–O bonds of ligand and water molecules. We can instead follow the angles between two pairs of opposite oxygen atoms belonging to different 2,2'-bpdo ligands as a measure of axiality. The angles are almost identical and follow the same pattern for **1**, **3**, **5** – Nd(III) and **2**, **4**, **6** – Ce(III) analogs; therefore, we discuss precisely the first series here; for the second, please refer to the respective Tables (above). For the first pair of the opposite atoms, in **1**: O1–Nd1–O4 = 129.28 °, **3**: O3–Nd1–O5 = 122.50 °, **5**: O1–Nd1–O4 = 104.78 ° indicating the best arrangement for **1** and the worst for **5**, nevertheless angles of the second pairs are, in **1**: O2–Nd1–O3 = 131.28 °, **3**: O6–Nd1–O4 = 134.00 °, **5**: O2–Nd1–O3 = 105.80 ° follow reversed behavior for **2** and **1** – in the case of **2** being slightly more linear. It is, therefore, ambiguous to judge the relative performance of the investigated molecules purely by this heuristic method. As it will turn out later, thanks to thorough experimental characteristics and theoretical simulations, **5** is indeed the worst-performing nanomagnet in this series, as the angles may suggest, while the differences between **1** and **3** are much more subtle.

It is worth mentioning here that, after the calculations of the main magnetic axes and their alignment within the metal complex, we found that the origin of significant anisotropy in obtained compounds **1**–**4** is first related to the favorable opposite alignment of two 2,2'-bpdo ligands, and, second, related to the role of pseudo-C_3 symmetry of the trigonal prismatic fragments of eight-coordinated lanthanide complexes that can be described as strongly distorted tricapped trigonal prisms (see the main text for expanded discussion in this regard).

Table S10. Results of Continuous Shape Measure (CShM) analysis for six-coordinated cobalt(III) complexes in **1–6** (for details regarding the presented parameters, see the comment below Table S11).

**Compound: metal center** (Figures S4–S8)	**CShM parameter***					
	**HP-6**	**PPY-6**	**OC-6**	**TPR-6**	**JPPY-6**	**Geometry**
**1: Co1**	32.731	27.779	**0.217**	14.746	31.435	**OC-6**
**2: Co1**	32.340	27.572	**0.234**	14.762	31.197	**OC-6**
**3: Co1**	30.955	26.256	**0.632**	14.959	30.396	**OC-6**
**4: Co1**	31.461	28.145	**0.166**	15.801	32.001	**OC-6**
**5: Co1**	33.422	28.699	**1.407**	16.976	31.815	**OC-6**
**5: Co2**	33.301	28.990	**1.468**	16.933	32.225	**OC-6**
**6: Co1**	33.786	28.609	**1.426**	16.912	31.746	**OC-6**
**6: Co2**	33.452	29.259	**1.348**	16.977	32.493	**OC-6**

Table S11. Results of Continuous Shape Measure (CShM) analysis for nine-coordinated lanthanide(III) complexes in **1–6**. Only the five most likely coordination polyhedra are presented.

**Compound: metal center** (Figures S4–S8)	**CShM parameter***					
	**JCSAPR-9**	**CSAPR-9**	**JTCTPR-9**	**TCTPR-9**	**MFF-9**	**Geometry**
**1: Nd1**	1.446	**0.671**	2.532	1.073	1.447	**CSAPR-9**
**2: Ce1**	1.521	**0.685**	2.572	1.102	1.486	**CSAPR-9**
**3: Nd1**	2.040	1.292	3.584	**1.214**	1.545	**TCTPR-9**
**4: Ce1**	2.059	1.279	3.130	**1.208**	1.327	**TCTPR-9**
**5: Nd1**	1.288	**0.344**	3.045	1.350	0.821	**CSAPR-9**
**6: Ce1**	1.328	**0.411**	3.091	1.510	0.858	**CSAPR-9**

*Continuous Shape Measure (CShM) parameters:[S7,S8]

six-coordinated complexes

CShM HP-6 – a parameter corresponding to the hexagon (D_{6h} symmetry)

CShM PPY-6 – a parameter corresponding to the pentagonal pyramid (C_{5v})

CShM OC-6 – a parameter corresponding to the octahedron (O_h)

CShM TPR-6 – a parameter corresponding to the trigonal prism (D_{3h})

CShM JPPY-6 – a parameter corresponding to the Johnson pentagonal pyramid (C_{5v})

nine-coordinated complexes[S9]

CShM JCSAPR-9 – a parameter corresponding to the Johnson capped square antiprism (C_{4v})

CShM CSAPR-9 – a parameter corresponding to the capped square antiprism (C_{4v})

CShM JTCTPR-9 – a parameter corresponding to the Johnson tricapped trigonal prism (D_{3h})

CShM TCTPR-9 – a parameter corresponding to the tricapped trigonal prism (D_{3h})

CShM MFF-9 – a parameter corresponding to the Muffin (C_s)

Comment to Tables S10 and S11: Continuous Shape Measure (CShM) analysis for Co^{III}, Ce^{III}, and Nd^{III} complexes was performed using the SHAPE software ver. 2.1.21.[S7] The CShM parameter represents the distortion from an ideal geometry. It equals 0 for the model polyhedron. Multiple geometries with similar parameters in the case of Nd1 in **3** and Ce1 in **4** suggest that the structure is a mixture of the polyhedra with such parameters.

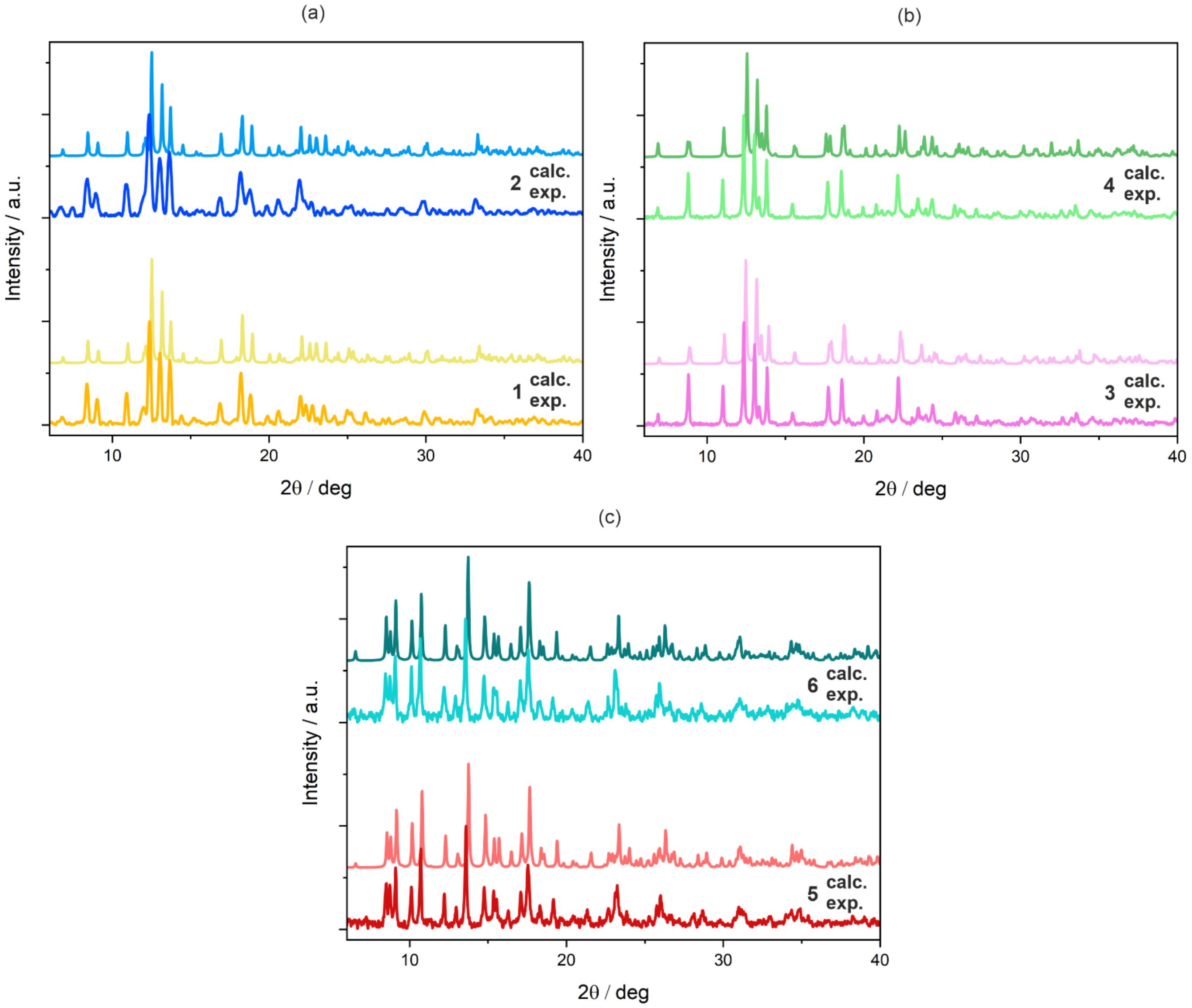

Figure S9. Comparison of experimental (exp.) and calculated (calc.) P-XRD patterns of the powder samples of **1**, **2** (a), **3**, **4** (b), **5**, and **6** (c) presented in the 2θ range of 5–40°. Calculated P-XRD patterns were simulated from the single crystal X-ray diffraction performed at 100(2) K (see Tables S2 and S3) using the Mercury (version 2022.2.0) software.

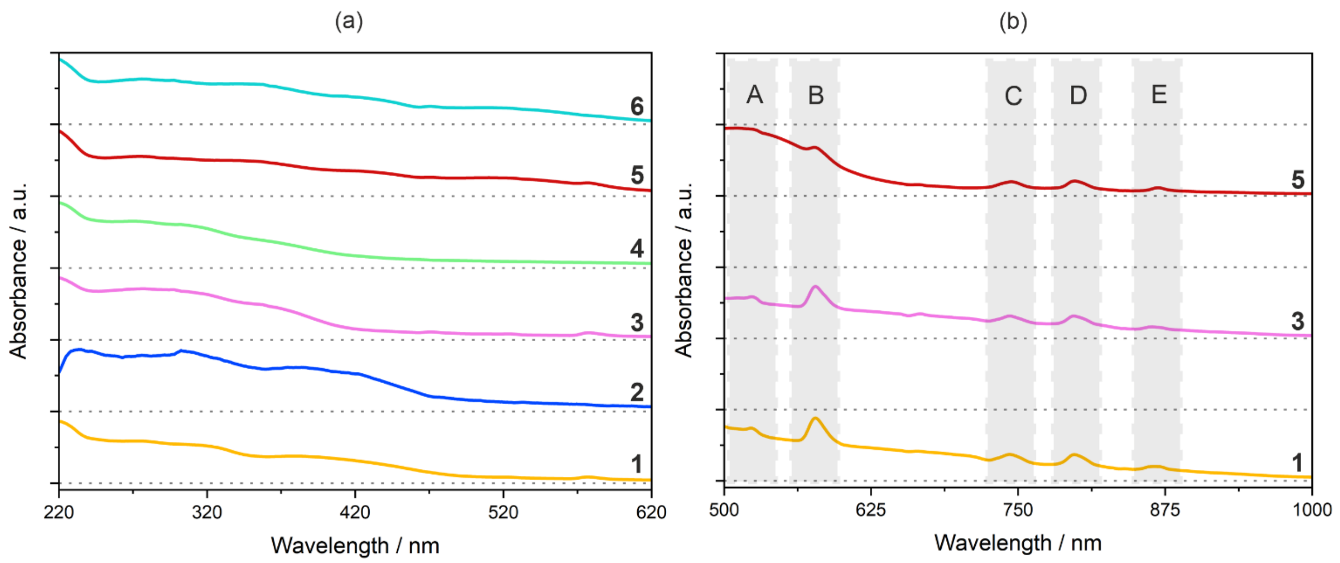

Figure S10. Solid-state UV-vis-NIR absorption spectra of **1**–**6**, recorded at room temperature in the 220–1000 nm wavelength range. Spectra have been divided into two ranges, including those showing absorption for the whole series of compounds in the 220–620 nm region (a), and the second one spanning the region from 500 to 1000 nm for the Nd(III)-containing samples exhibiting non-negligible light absorption for this part of the spectrum (b). Tiny sharp peaks in part (b) are labeled and described in Table S12 (below). The spectra of part (b) were measured separately with the larger amount of the respective sample as the related peaks are a few times weaker than the broad absorption bands shown in part (a). For theoretical simulations of the light absorption spectra of **1**, **3,** and **5**, please see Figures S36 and S37.

Table S12. Electron transitions associated with light absorption in the visible-to-NIR region of the spectra shown in Figure S10 for compounds containing Nd(III) centers, i.e., **1**, **3**, and **5**. The labels for the bands are indicated on the spectra in Figure S10b (see above).

Label for the absorption band (Figure S10b)	Peak maximum wavelength			Corresponding f-f electronic transitions[S10,S11]
	**1**	**3**	**5**	
A	523.0 nm	523.5 nm	525.5 nm	Nd^{III}: $^4I_{9/2} \longrightarrow {}^4G_{7/2}$, $^4G_{9/2}$, $^2K_{13/2}$
B	577.5 nm	577.5 nm	577.0 nm	Nd^{III}: $^4I_{9/2} \longrightarrow {}^2G_{7/2}$, $^4G_{5/2}$
C	743.0 nm	743.0 nm	744.0 nm	Nd^{III}: $^4I_{9/2} \longrightarrow {}^4S_{3/2}$, $^4F_{7/2}$
D	797.5 nm	797.5 nm	798.0 nm	Nd^{III}: $^4I_{9/2} \longrightarrow {}^2H_{9/2}$, $^4F_{5/2}$
E	867.0 nm	864.0 nm	869.0 nm	Nd^{III}: $^4I_{9/2} \longrightarrow {}^4F_{3/2}$

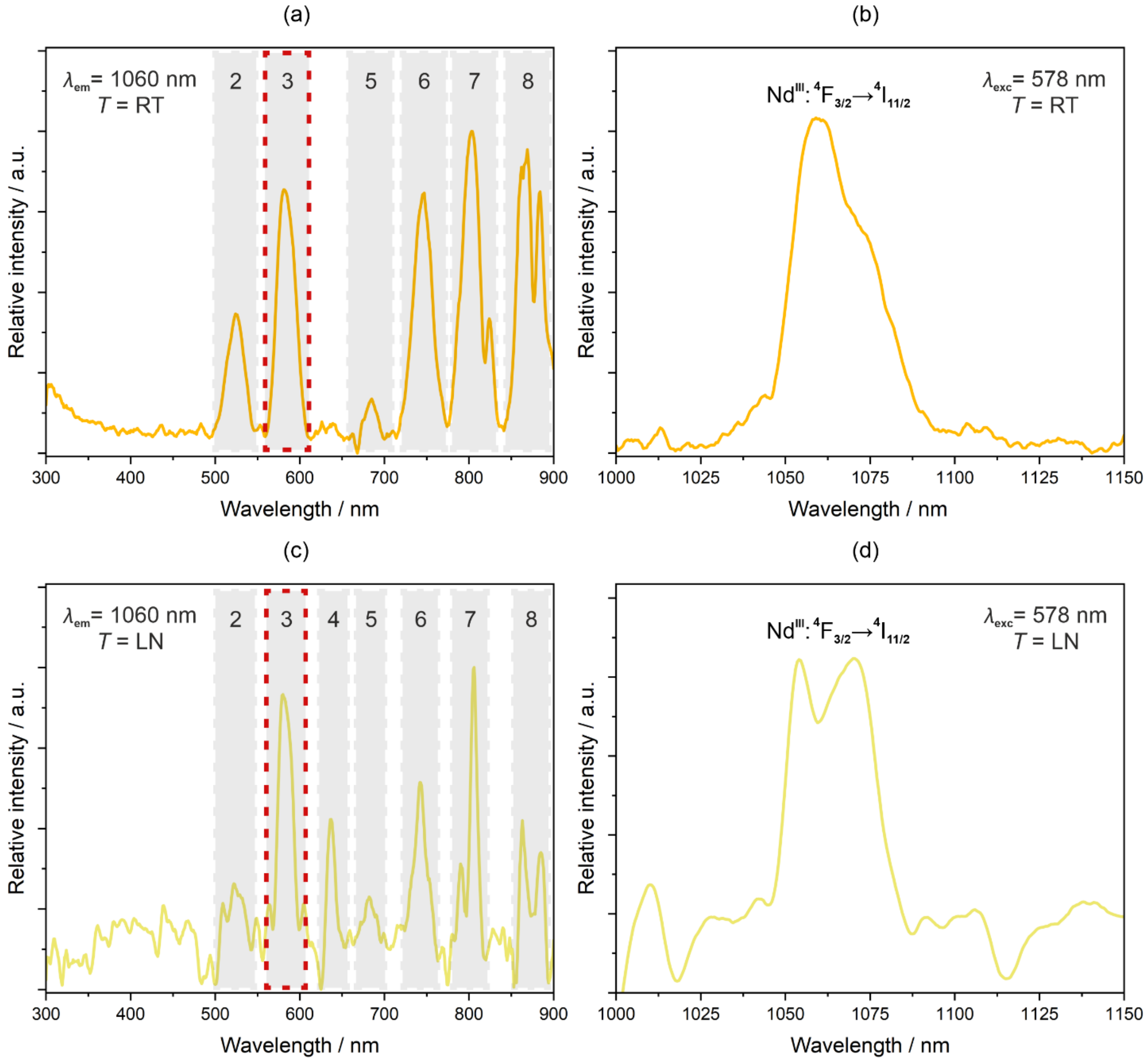

Figure S11. Solid-state excitation and emission spectra of compound **1**. Parts (a) and (c) present the excitation spectra for the monitored emission wavelength at 1060 nm, while parts (b) and (d) contain the emission spectra for an excitation wavelength of 578 nm (marked in red on the excitation spectra). Spectra were recorded at two temperatures, i.e., parts (a) and (b) correspond to room temperature (RT), while parts (c) and (d) correspond to the temperature of boiling liquid nitrogen (LN). The origin of the emission peak is depicted on the graph. The peaks in the excitation spectra are labeled (2–8); the corresponding assignment of these peaks to specific f-f electronic transitions is given in Table S13.

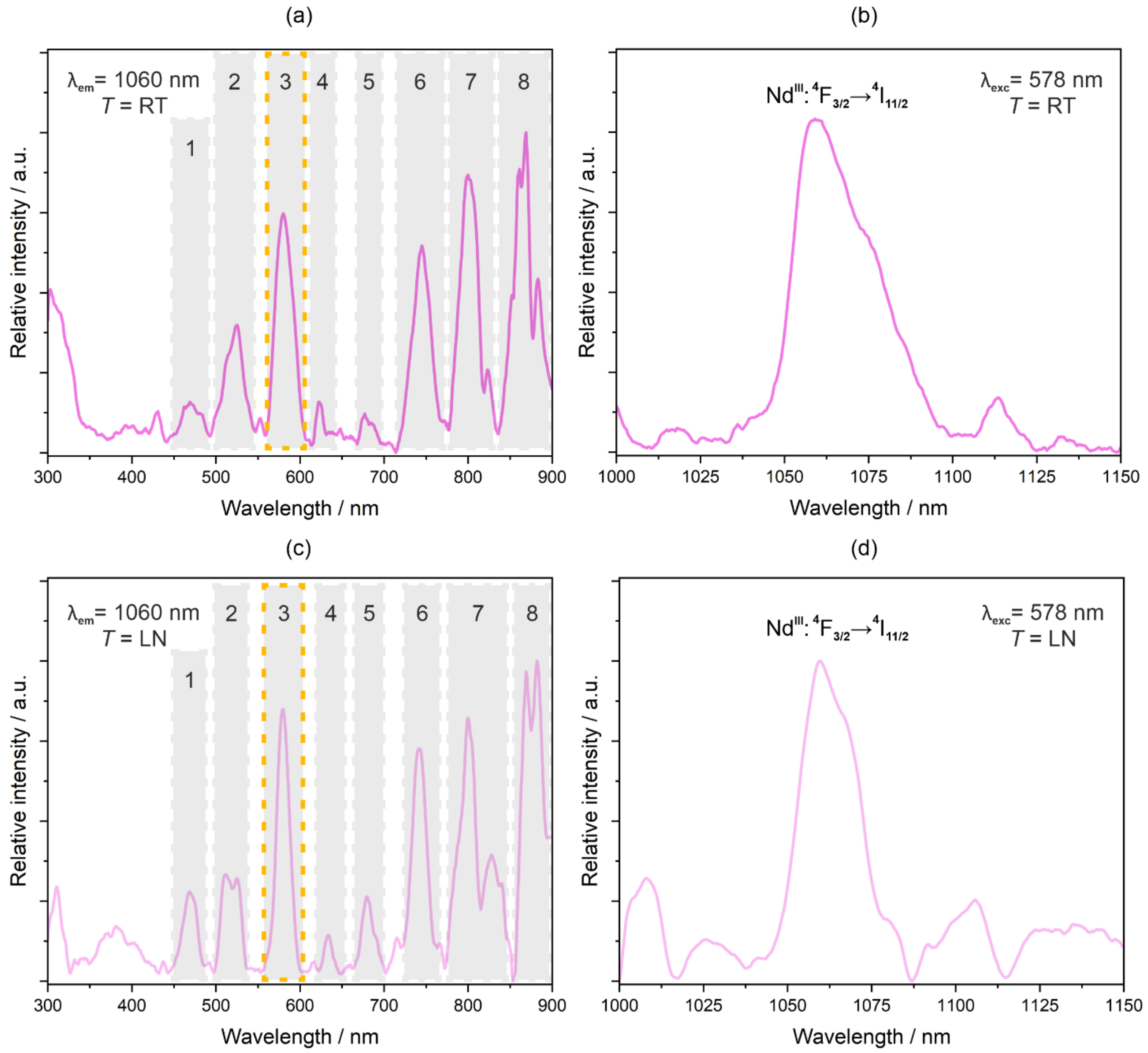

Figure S12. Solid-state excitation and emission spectra of compound **3**. Parts (a) and (c) present the excitation spectra for the monitored emission wavelength at 1060 nm, while parts (b) and (d) contain the emission spectra for an excitation wavelength of 578 nm (marked in orange on the excitation spectra). Spectra were recorded at two temperatures, i.e., parts (a) and (b) correspond to room temperature (RT), while parts (c) and (d) correspond to the temperature of boiling liquid nitrogen (LN). The origin of the emission peak is depicted on the graph. The peaks in the excitation spectra are labeled (1–8); the corresponding assignment of these peaks to specific f-f electronic transitions is given in Table S13.

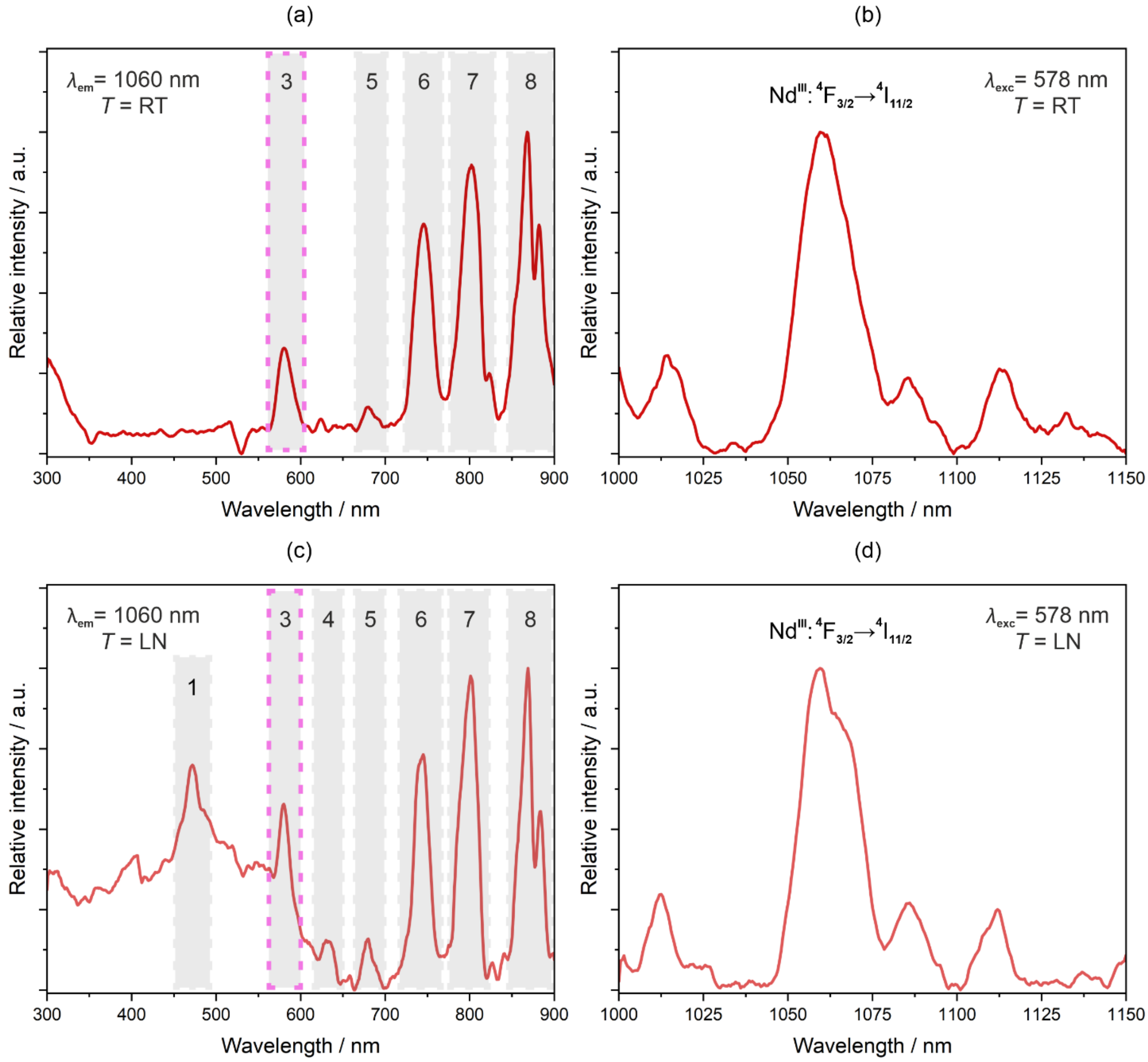

Figure S13. Solid-state excitation and emission spectra of compound **5**. Parts (a) and (c) present the excitation spectra for the monitored emission wavelength at 1060 nm, while parts (b) and (d) contain the emission spectra for an excitation wavelength of 578 nm (marked in pink on the excitation spectra). Spectra were recorded at two temperatures, i.e., parts (a) and (b) correspond to room temperature (RT), while parts (c) and (d) correspond to the temperature of boiling liquid nitrogen (LN). The origin of the emission peak is depicted on the graph. The peaks in the excitation spectra are labeled (1, and 3–8); the corresponding assignment of these peaks to specific f-f electronic transitions is given in Table S13.

Comment on Figures S11–S13: Due to the overall weak photoluminescence in reported compounds, it was challenging to get a reliable signal using the accessible experimental setup equipped with InGaAs-1650 analog NIR detector. Thus, only the strongest Nd(III)-centered NIR emission at around 1060 nm could be gathered. Even for this emission, the spectra had to be recorded by measuring lanthanum(III) chloride as a background to subtract the artifacts appearing for the used optical system. In the region of ca. 400 nm in excitation spectra recorded at LN temperature, a weak band, potentially originating from energy transfer from cobalt(III)–cyanido complexes to Nd(III) centers arises. However, with the employed experimental setup, this pathway for NIR emission could not be undoubtedly confirmed.

Table S13. Assignment of excitation peaks in the vis-NIR range of compounds **1**, **3**, and **5** to the specific Nd(III)-based f-f electronic transitions, gathered with the values of wavelengths of related emission maxima at RT (room temperature) and LN (liquid nitrogen temperature, see Figures S11–S13).

**Label for the excitation band (Figures S11–S13)**	**Temperature**	**Wavelengths of emission maxima**			**Corresponding f-f electronic transitions**[S10,S11]
		**1**	**3**	**5**	
1	RT	—	469 nm	—	Nd^{III}: $^4I_{9/2} \rightarrow {}^2G_{9/2}, {}^4G_{11/2}, {}^2K_{15/2}$
	LN	—	469 nm	472 nm	
2	RT	524 nm	525 nm	—	Nd^{III}: $^4I_{9/2} \rightarrow {}^4G_{7/2}, {}^4G_{9/2}, {}^2K_{13/2}$
	LN	522 nm	512 nm	—	
3	RT	581 nm	580 nm	580 nm	Nd^{III}: $^4I_{9/2} \rightarrow {}^2G_{7/2}, {}^4G_{5/2}$
	LN	580 nm	580 nm	580 nm	
4	RT	—	622 nm	—	Nd^{III}: $^4I_{9/2} \rightarrow {}^2H_{11/2}$
	LN	637 nm	634 nm	630 nm	
5	RT	685 nm	677 nm	680 nm	Nd^{III}: $^4I_{9/2} \rightarrow {}^4F_{9/2}$
	LN	682 nm	680 nm	680 nm	
6	RT	747 nm	745 nm	746 nm	Nd^{III}: $^4I_{9/2} \rightarrow {}^4S_{3/2}, {}^4F_{7/2}$
	LN	742 nm	741 nm	745 nm	
7	RT	803 nm	800 nm	802 nm	Nd^{III}: $^4I_{9/2} \rightarrow {}^2H_{9/2}, {}^4F_{5/2}$
	LN	806 nm	800 nm	801 nm	
8	RT	869 nm	869 nm	868 nm	Nd^{III}: $^4I_{9/2} \rightarrow {}^4F_{3/2}$
	LN	863 nm	882 nm	869 nm	

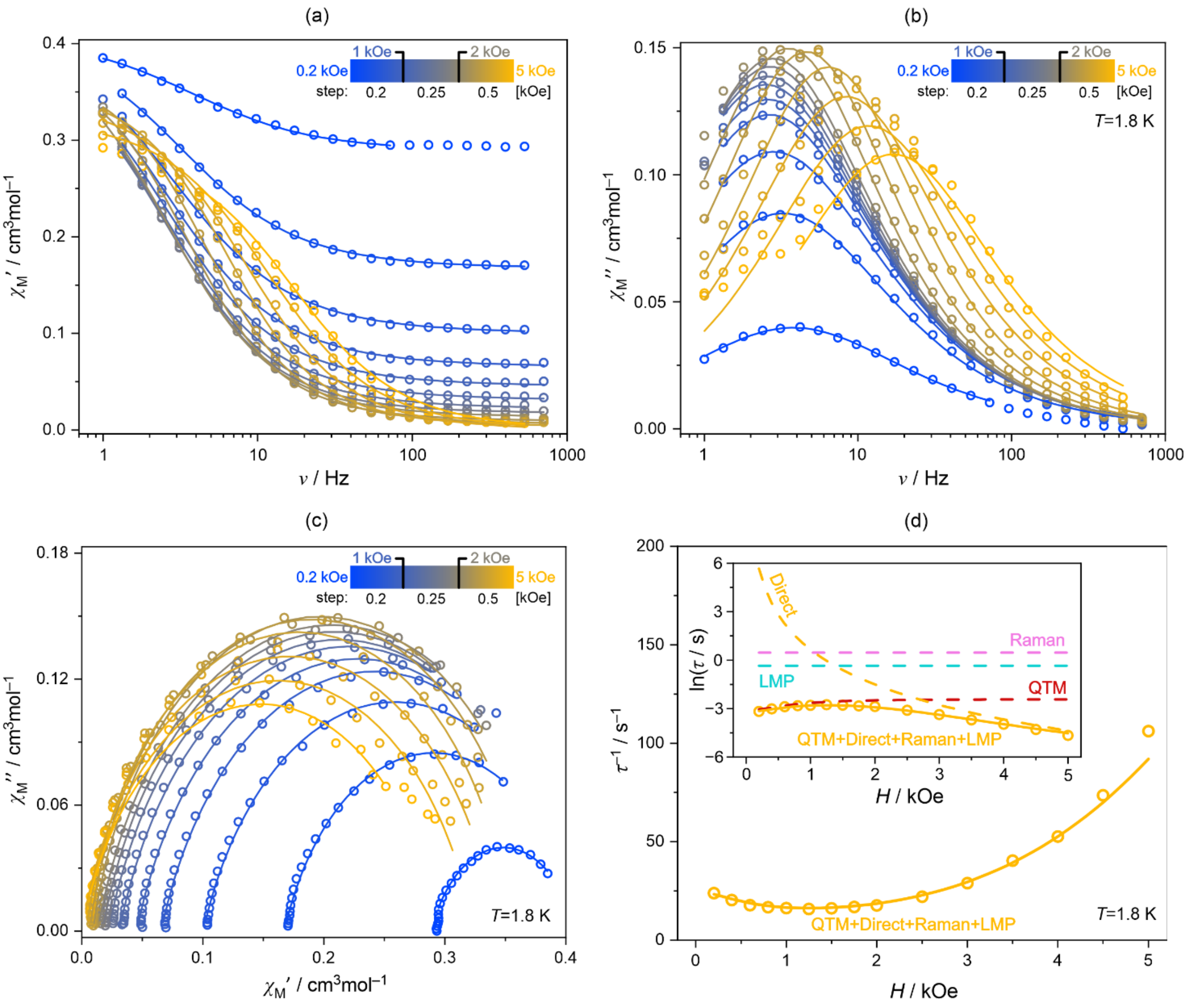

Figure S14. Field (direct-current, *dc*) variable alternating-current (*ac*) magnetic characteristics of **1** at T = 1.8 K (H_{ac} = 3 Oe), including the frequency dependences of the in-phase, χ_M', (a) and the out-of-phase, χ_M'', (b) components of complex magnetic susceptibility at the indicated *dc* fields, shown together with the corresponding Argand plots (c), and the field dependence of resulting relaxation times (d, presented in the form of $\tau^{-1}(H)$ in the main part and the form of ln(τ) versus H in the inset). Solid lines in (a–c) represent the best-fit curves corresponding to the generalized Debye model (equation (1) in the main article). The solid line in (d) represents the best fit to the model (equation (2) in the main article) involving four different magnetic relaxation processes, including the Direct process, QTM, Raman process, and local-mode process (LMP). The contributions from each of these processes to the overall magnetic relaxation are depicted by dashed lines (d, the inset). The fitting procedure was done simultaneously with the fitting of the related temperature dependence of relaxation time (Figure S15). The resulting best-fit parameters are gathered in Tables 1 and S14. All steps of the analysis were performed using the relACs program.[S12]

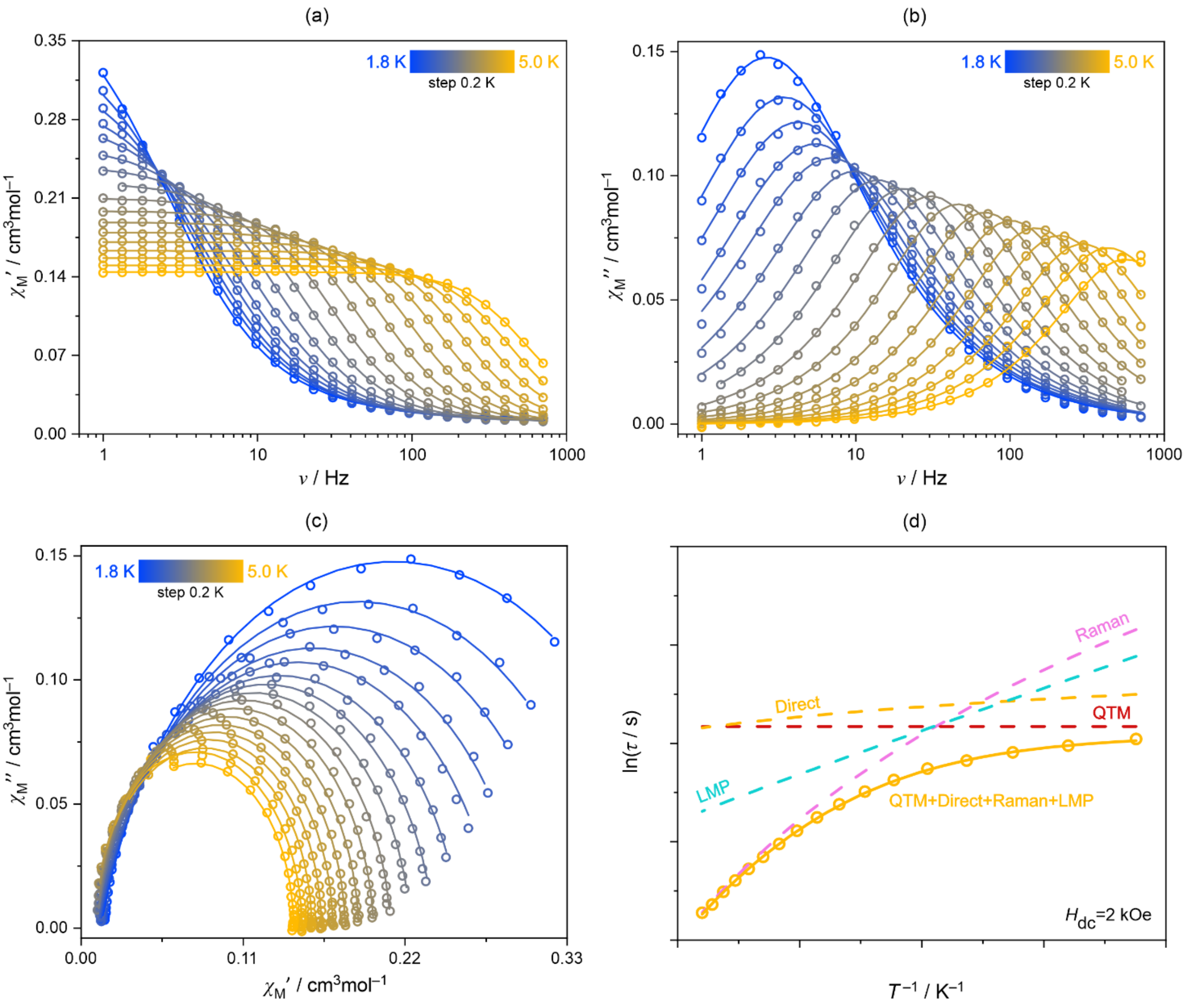

Figure S15. Temperature variable alternating-current (*ac*) magnetic characteristics of **1** at H_{dc} = 2 kOe (H_{ac} = 3 Oe), including the frequency dependences of the in-phase, χ_M', (a) and the out-of-phase, χ_M'', (b) components of complex magnetic susceptibility at the indicated temperatures, shown together with the corresponding Argand plots (c), and the temperature dependence of resulting relaxation times (d). Solid lines in (a–c) represent the best-fit curves corresponding to the generalized Debye model (equation (1) in the main article). The solid line in (d) represents the best fit to the model (equation (2) in the main article) involving four different magnetic relaxation processes, including the Direct process, QTM, Raman process, and local-mode process (LMP). The contributions from each of these processes to the overall magnetic relaxation are depicted by dashed lines. The fitting procedure was done simultaneously with the fitting of the related field dependence of relaxation time (Figure S14). The resulting best-fit parameters are gathered in Tables 1 and S14. All steps of the analysis were performed using the relACs program.[S12]

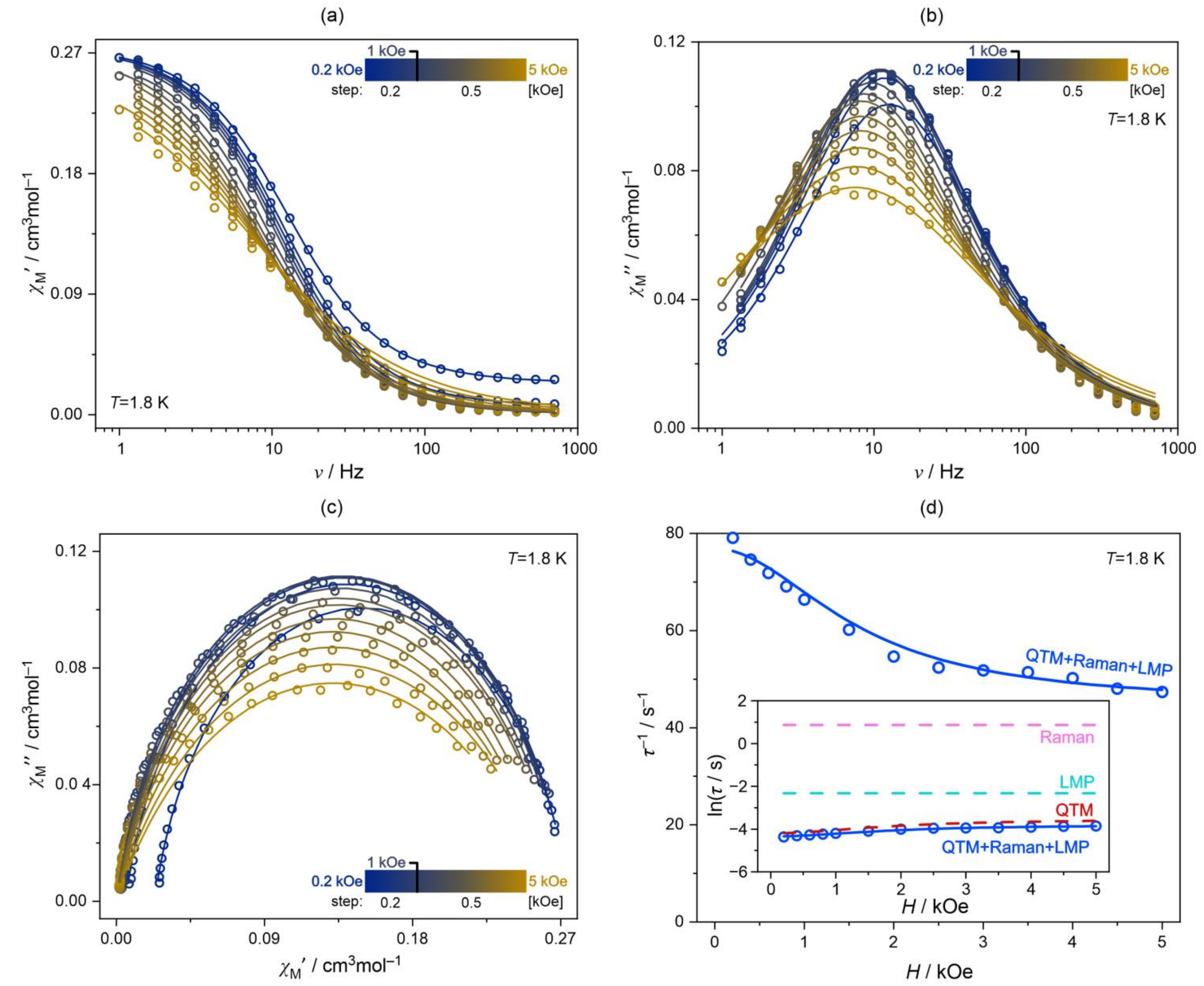

Figure S16. Field (direct-current, *dc*) variable alternating-current (*ac*) magnetic characteristics of **2** at T = 1.8 K (H_{ac} = 3 Oe), including the frequency dependences of the in-phase, χ_M', (a) and the out-of-phase, χ_M'', (b) components of complex magnetic susceptibility at the indicated *dc* fields, shown together with the corresponding Argand plots (c), and the field dependence of resulting relaxation times (d, presented in the form of $\tau^{-1}(H)$ in the main part and the form of ln(τ) versus H in the inset). Solid lines in (a–c) represent the best-fit curves corresponding to the generalized Debye model (equation (1) in the main article). The solid line in (d) represents the best fit to the model (equation (2) in the main article) involving four different magnetic relaxation processes, including the Direct process, QTM, Raman process, and local-mode process (LMP) (the Direct process was not necessary to be included for the case of this compound). The contributions from each of these processes to the overall magnetic relaxation are depicted by dashed lines (d, the inset). The fitting procedure was done simultaneously with the fitting of the related temperature dependence of relaxation time (Figure S17). The resulting best-fit parameters are gathered in Tables 1 and S14. All steps of the analysis were performed using the relACs program.[S12]

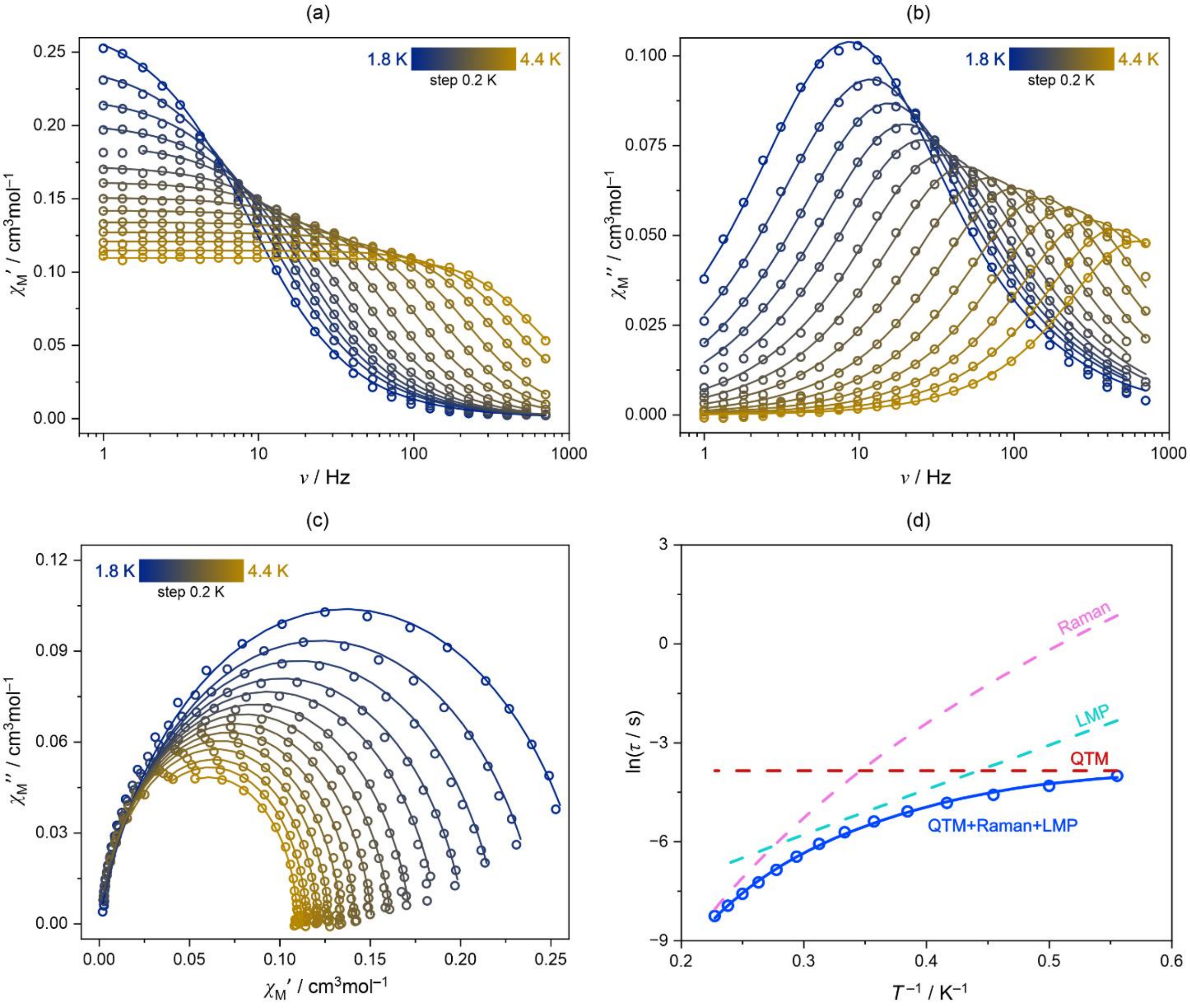

Figure S17. Temperature variable alternating-current (*ac*) magnetic characteristics of **2** at H_{dc} = 2 kOe (H_{ac} = 3 Oe), including the frequency dependences of the in-phase, χ_M', (a) and the out-of-phase, χ_M'', (b) components of complex magnetic susceptibility at the indicated temperatures, shown together with the corresponding Argand plots (c), and the temperature dependence of resulting relaxation times (d). Solid lines in (a–c) represent the best-fit curves corresponding to the generalized Debye model (equation (1) in the main article). The solid line in (d) represents the best fit to the model (equation (2) in the main article) involving four different magnetic relaxation processes, including the Direct process, QTM, Raman process, and local-mode process (LMP) (the Direct process was not necessary to be included for the case of this compound). The contributions from each of these processes to the overall magnetic relaxation are depicted by dashed lines. The fitting procedure was done simultaneously with the fitting of the related field dependence of relaxation time (Figure S16). The resulting best-fit parameters are gathered in Tables 1 and S14. All steps of the analysis were performed using the relACs program.[S12]

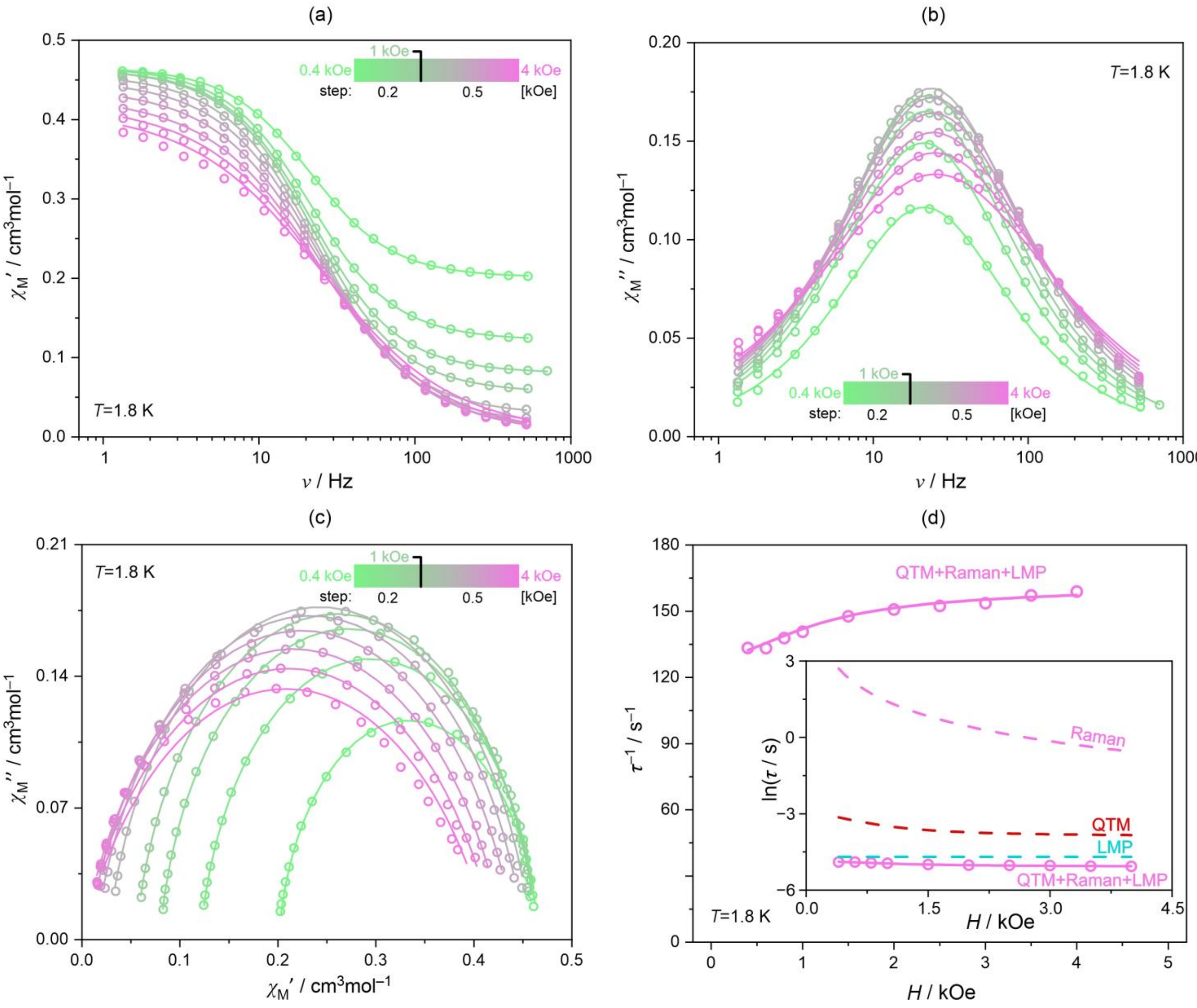

Figure S18. Field (direct-current, *dc*) variable alternating-current (*ac*) magnetic characteristics of **3** at T = 1.8 K (H_{ac} = 3 Oe), including the frequency dependences of the in-phase, χ_M', (a) and the out-of-phase, χ_M'', (b) components of complex magnetic susceptibility at the indicated *dc* fields, shown together with the corresponding Argand plots (c), and the field dependence of resulting relaxation times (d, presented in the form of $\tau^{-1}(H)$ in the main part and the form of ln(τ) versus H in the inset). Solid lines in (a–c) represent the best-fit curves corresponding to the generalized Debye model (equation (1) in the main article). The solid line in (d) represents the best fit to the model (equation (2) in the main article) involving four different magnetic relaxation processes, including the Direct process, QTM, Raman process, and local-mode process (LMP) (the Direct process was not necessary to be included for the case of this compound). The contributions from each of these processes to the overall magnetic relaxation are depicted by dashed lines (d, the inset). The fitting procedure was done simultaneously with the fitting of the related temperature dependence of relaxation time (Figure S19). The resulting best-fit parameters are gathered in Tables 1 and S14. All steps of the analysis were performed using the relACs program.[S12]

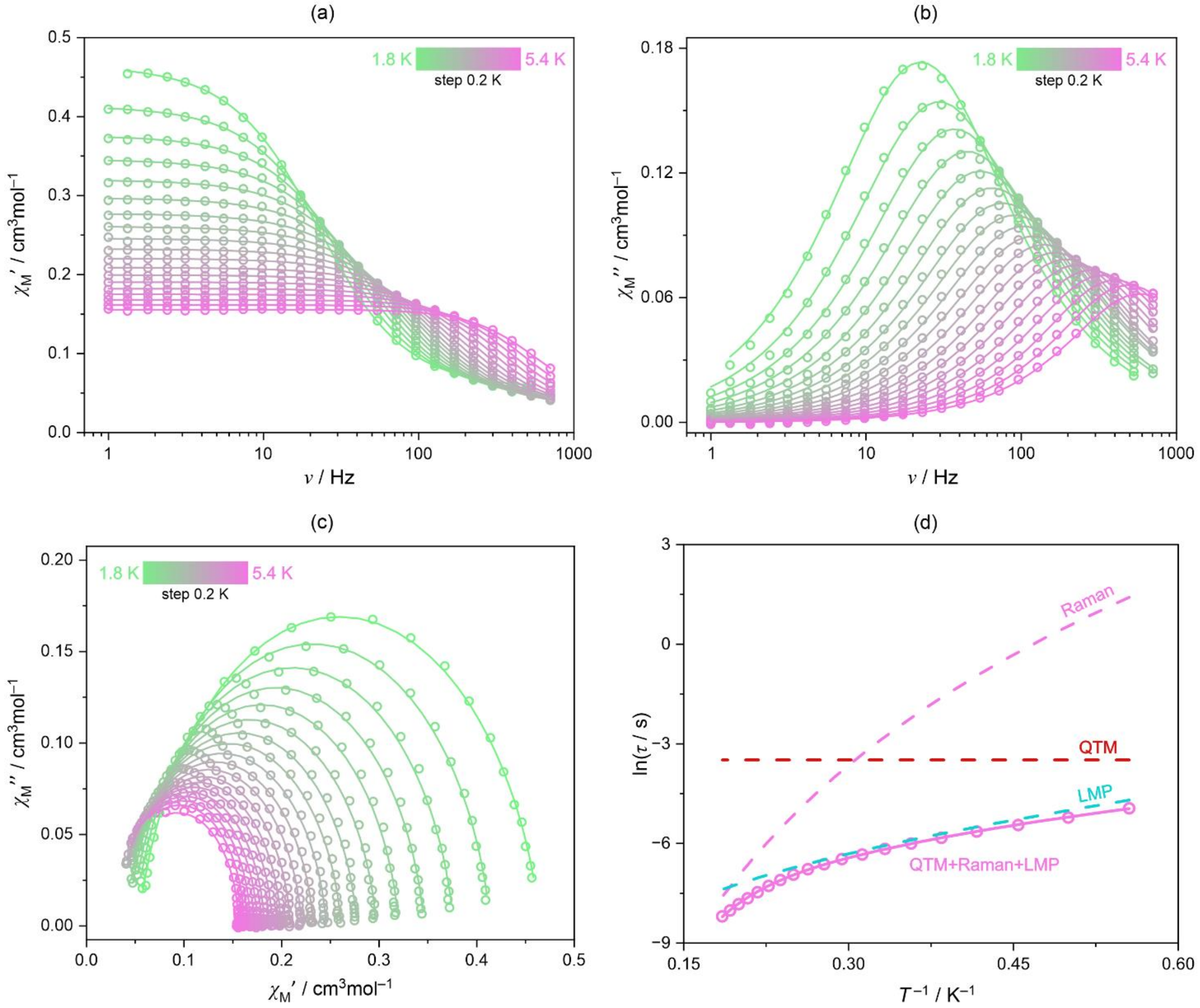

Figure S19. Temperature variable alternating-current (*ac*) magnetic characteristics of **3** at H_{dc} = 1 kOe (H_{ac} = 3 Oe), including the frequency dependences of the in-phase, χ_M', (a) and the out-of-phase, χ_M'', (b) components of complex magnetic susceptibility at the indicated temperatures, shown together with the corresponding Argand plots (c), and the temperature dependence of resulting relaxation times (d). Solid lines in (a–c) represent the best-fit curves corresponding to the generalized Debye model (equation (1) in the main article). The solid line in (d) represents the best fit to the model (equation (2) in the main article) involving four different magnetic relaxation processes, including the Direct process, QTM, Raman process, and local-mode process (LMP) (the Direct process was not necessary to be included for the case of this compound). The contributions from each of these processes to the overall magnetic relaxation are depicted by dashed lines. The fitting procedure was done simultaneously with the fitting of the related field dependence of relaxation time (Figure S18). The resulting best-fit parameters are gathered in Tables 1 and S14. All steps of the analysis were performed using the relACs program.[S12]

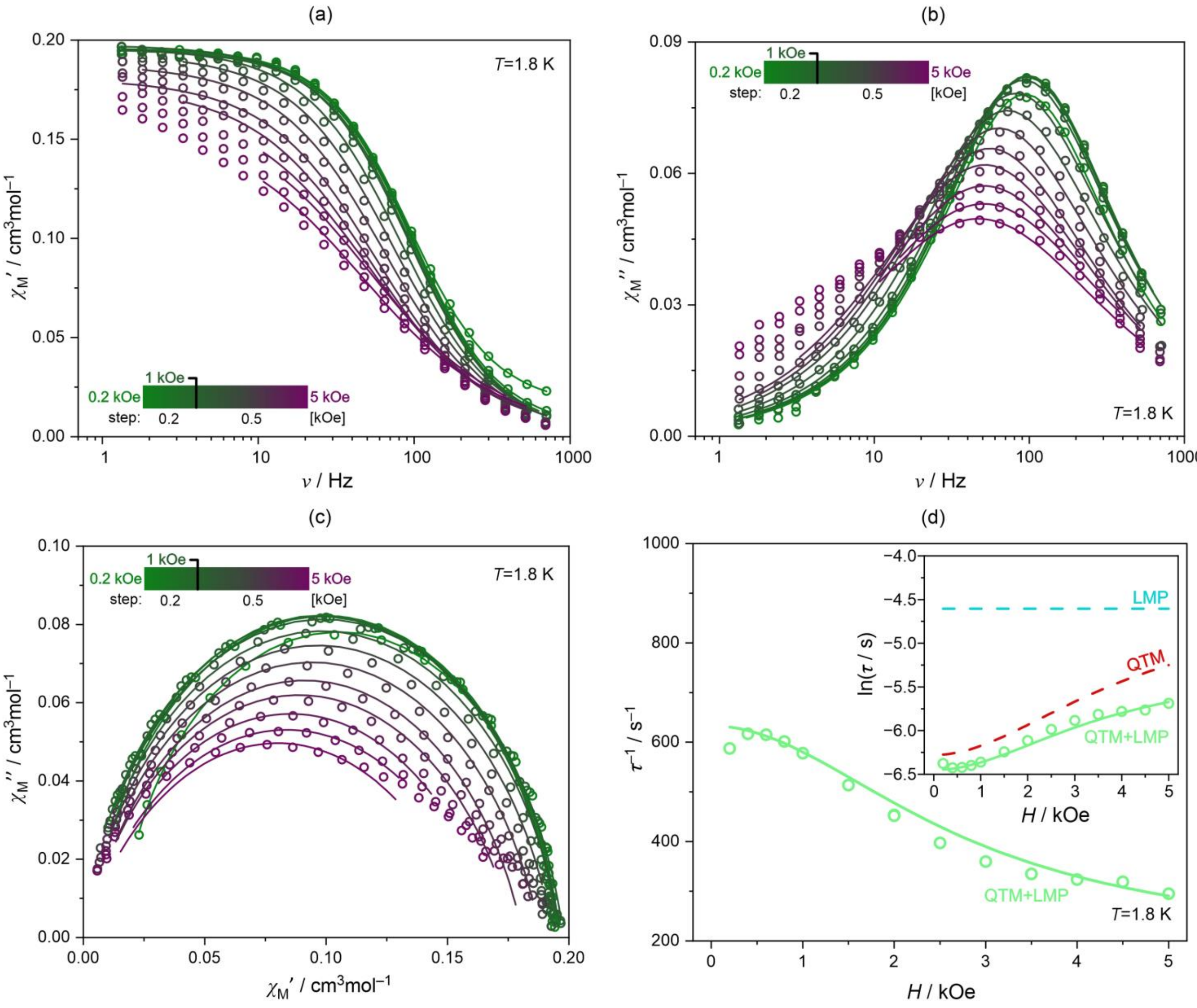

Figure S20. Field (direct-current, *dc*) variable alternating-current (*ac*) magnetic characteristics of **4** at T = 1.8 K (H_{ac} = 3 Oe), including the frequency dependences of the in-phase, χ_M', (a) and the out-of-phase, χ_M'', (b) components of complex magnetic susceptibility at the indicated *dc* fields, shown together with the corresponding Argand plots (c), and the field dependence of resulting relaxation times (d, presented in the form of $\tau^{-1}(H)$ in the main part and the form of ln(τ) versus H in the inset). Solid lines in (a–c) represent the best-fit curves corresponding to the generalized Debye model (equation (1) in the main article). The solid line in (d) represents the best fit to the model (equation (2) in the main article) involving four different magnetic relaxation processes, including the Direct process, QTM, Raman process, and local-mode process (LMP) (the Direct process as well as the Raman process were not necessary to be included for the case of this compound). The contributions from each of these processes to the overall magnetic relaxation are depicted by dashed lines (d, the inset). The fitting procedure was done simultaneously with the fitting of the related temperature dependence of relaxation time (Figure S21). The resulting best-fit parameters are gathered in Tables 1 and S14. All steps of the analysis were performed using the relACs program.[S12]

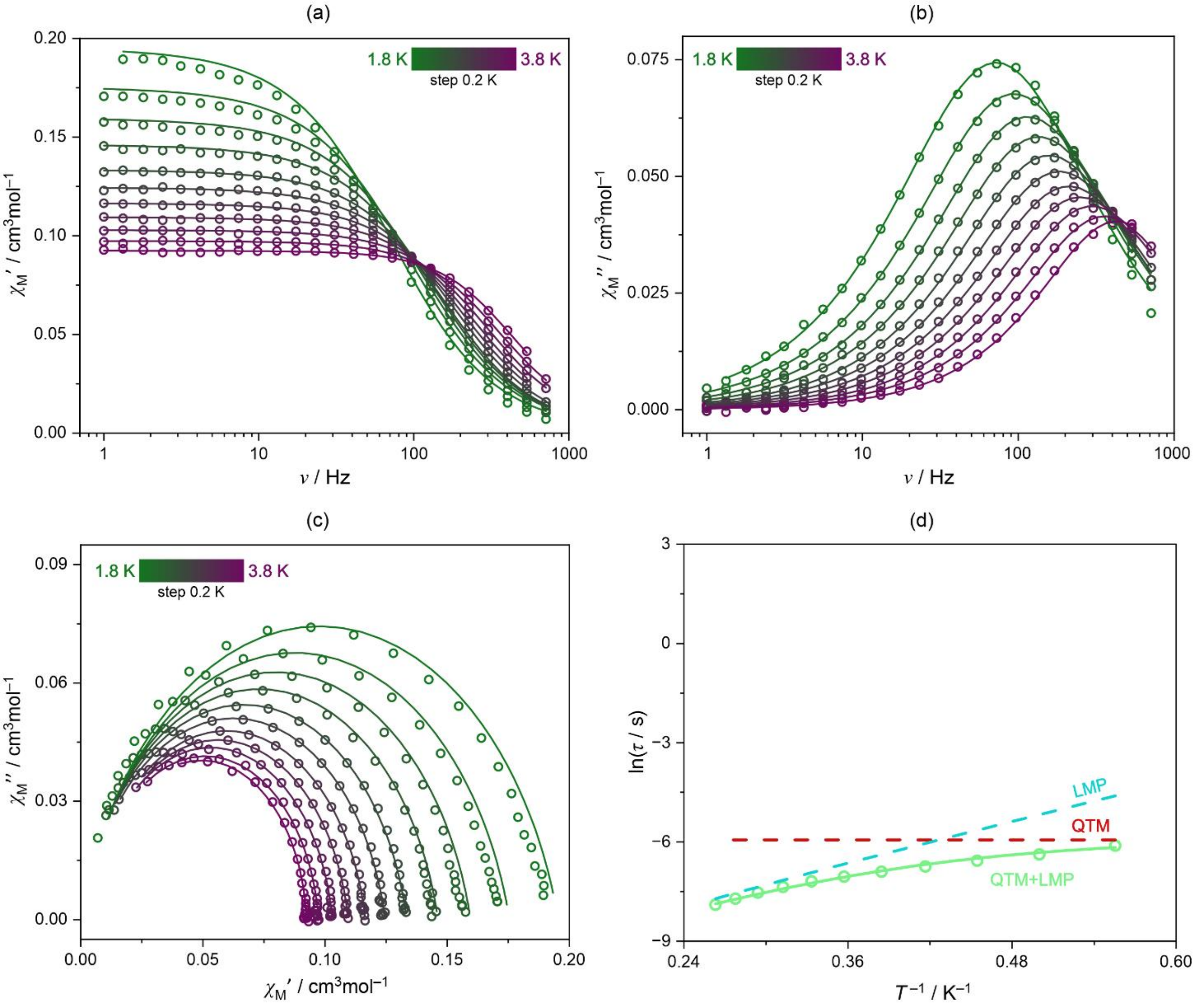

Figure S21. Temperature variable alternating-current (*ac*) magnetic characteristics of **4** at H_{dc} = 2 kOe (H_{ac} = 3 Oe), including the frequency dependences of the in-phase, χ_M', (a) and the out-of-phase, χ_M'', (b) components of complex magnetic susceptibility at the indicated temperatures, shown together with the corresponding Argand plots (c), and the temperature dependence of resulting relaxation times (d). Solid lines in (a–c) represent the best-fit curves corresponding to the generalized Debye model (equation (1) in the main article). The solid line in (d) represents the best fit to the model (equation (2) in the main article) involving four different magnetic relaxation processes, including the Direct process, QTM, Raman process, and local-mode process (LMP) (the Direct process as well as the Raman process were not necessary to be included for the case of this compound). The contributions from each of these processes to the overall magnetic relaxation are depicted by dashed lines. The fitting procedure was done simultaneously with the fitting of the related field dependence of relaxation time (Figure S20). The resulting best-fit parameters are gathered in Tables 1 and S14. All steps of the analysis were performed using the relACs program.[S12]

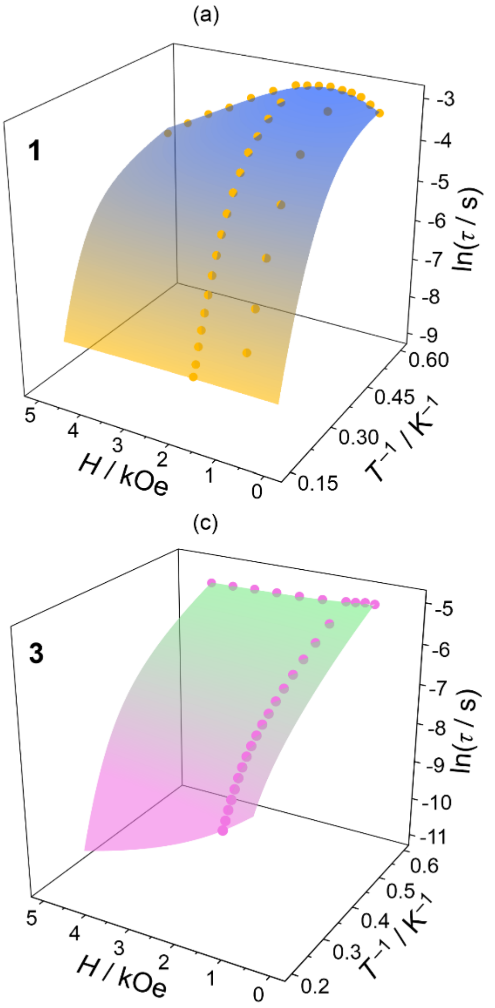

Figure S22. Three-dimensional views of the surface of multivariable $\tau(H, T)$ dependences of **1** (a), **2** (b), **3** (c), and **4** (d), representing the best-fit results following the equation (2) (see the main text), shown together with the experimental points (colored circles) of the relaxation times obtained from the *ac* magnetic studies (Figures S14–S21). The related best-fit parameters are gathered in Tables 1 and S14. Note that for compound **1** (a), we used the additional set of *ac* data gathered at H_{dc} = 1 kOe (not shown in Figures S14–S21). The presented hypersurfaces represent the 3-D fitting performed within the relACs program.[S12]

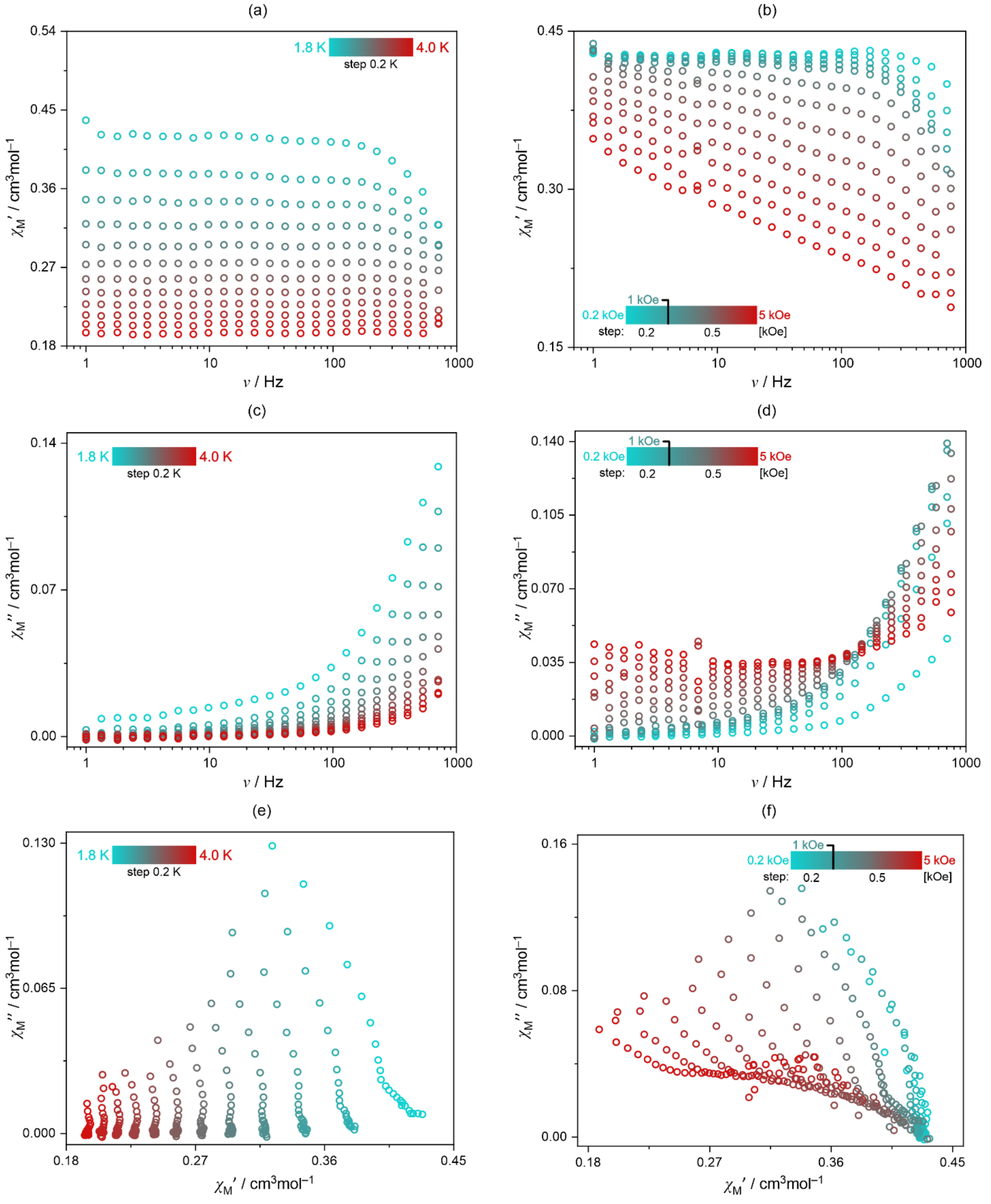

Figure S23. Temperature variable *ac* magnetic characteristics under the *dc* field of 1 kOe of **5**, including the frequency dependences of the in-phase, χ_M', (a) and the out-of-phase, χ_M'', (c) components of complex magnetic susceptibility at the indicated temperatures, shown together with the corresponding Argand plots (e), and field (dc) variable *ac* magnetic characteristics at T = 1.8 K of **5**, including the frequency dependences of the in-phase, χ_M', (b) and the out-of-phase, χ_M'', (d) components of complex magnetic susceptibility at the indicated *dc* fields, shown together with the corresponding Argand plots (f). Due to the lack of distinct maxima of the χ_M'', the presented experimental data was not fitted.

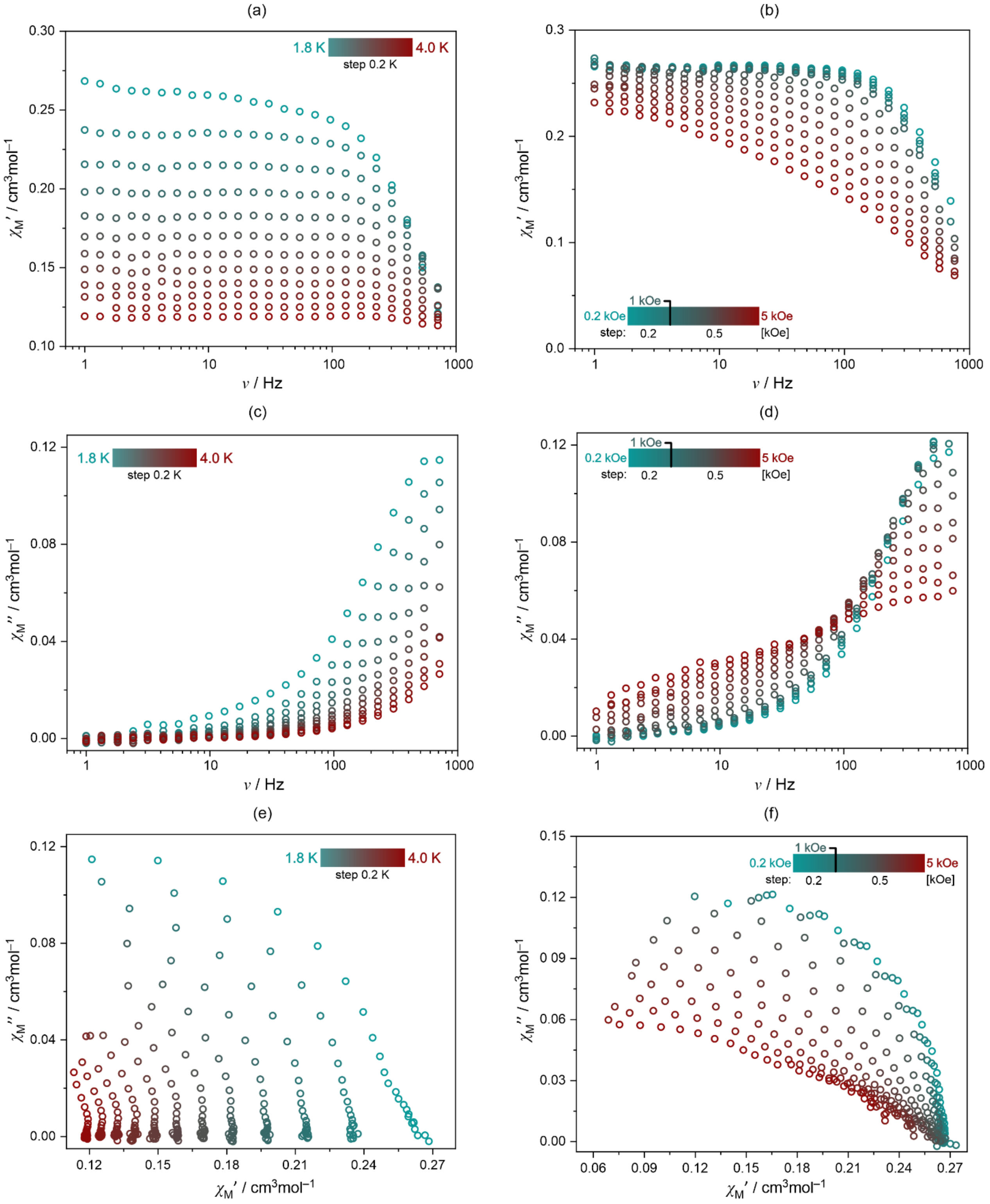

Figure S24. Temperature variable *ac* magnetic characteristics under the *dc* field of 1 kOe of **6**, including the frequency dependences of the in-phase, χ_M', (a) and the out-of-phase, χ_M'', (c) components of complex magnetic susceptibility at the indicated temperatures, shown together with the corresponding Argand plots (e), and field (dc) variable *ac* magnetic characteristics at *T* = 1.8 K of **6**, including the frequency dependences of the in-phase, χ_M', (b) and the out-of-phase, χ_M'', (d) components of complex magnetic susceptibility at the indicated *dc* fields, shown together with the corresponding Argand plots (f). Due to the lack of distinct maxima of the χ_M'', the presented experimental data was not fitted.

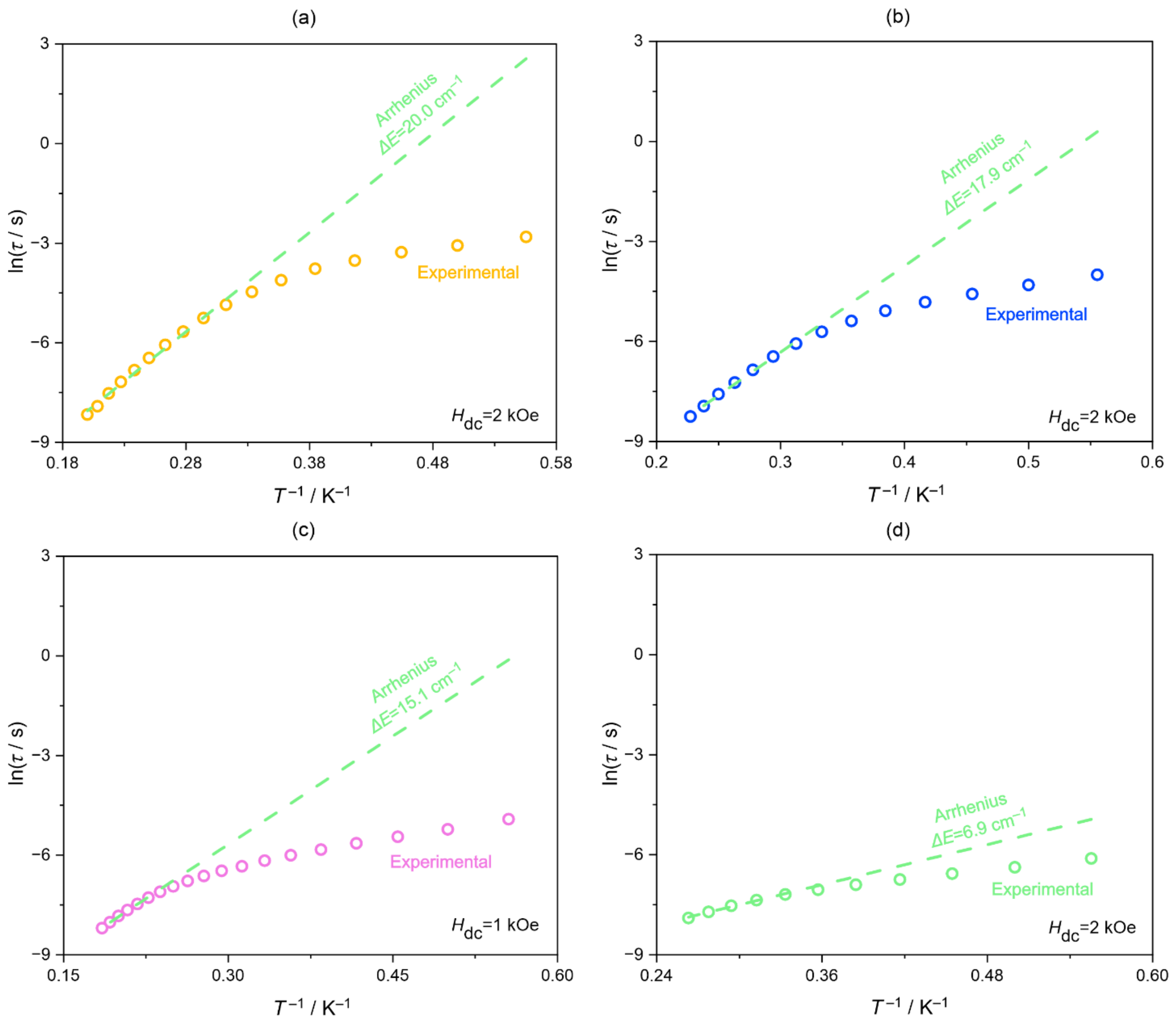

Figure S25. Results of the fitting of the high-temperature range of the temperature dependences of magnetic relaxation times of **1** (a), **2** (b), **3** (c), and **4** (d), using only the Arrhenius-type contribution related to a possible Orbach relaxation process (see equation (3) in the main text). The resulting energy barriers (*ΔE*) were indicated above the respective best-fit curves (dashed lines). The experimental points (empty circles) come from the *ac* magnetic studies of each compound (see Figures S15, S17, S19, and S21 for comparison).

Table S14. The best-fit parameters of the 3-D fitting of combined results of field- and temperature-dependent magnetic relaxation times, $\tau(H, T)$, for **1**–**4** according to the model (equation (2) in the main article) involving four different magnetic relaxation processes, including the Direct process (parameters given in yellow color), QTM (parameters given in red color), Raman process (parameters given in pink color), and local-mode process (LMP, parameters given in light blue color). The related best-fit curves are presented in Figures S14–S22.

Compound	1	2	3	4
A_{dir} / $s^{-1}\cdot K^{-1}\cdot Oe^{-m}$	$1.2(7)\cdot10^{-10}$	-	-	-
m	3.120(5)	-	-	-
B_1 / s^{-1}	22(2)	66(3)	19(3)	533(72)
B_2 / Oe^{-2}	$2.8(4)\cdot10^{-6}$	$3.8(1)\cdot10^{-7}$	$7.8(4)\cdot10^{-7}$	$1.3(4)\cdot10^{-7}$
B_3 / Oe^{-2}	$1.4(1)\cdot10^{-6}$	$2.0(1)\cdot10^{-7}$	$1.9(2)\cdot10^{-6}$	$2.2(4)\cdot10^{-8}$
C_{Raman} / $s^{-1}\cdot K^{-n}\cdot Oe^{-k}$	0.004(4)	0.001(2)	$1.09(2)\cdot10^{-7}$	-
n	8.4(6)	9(1)	8.2(2)	-
k	0	0	1.41(6)	0
$\hbar\omega$ / cm^{-1}	8.9(9)	9(1)	3(1)	6(1)
D / s^{-1}	$1.7(4)\cdot10^{3}$	$1(1)\cdot10^{4}$	$1.3(7)\cdot10^{3}$	$2.8(3)\cdot10^{4}$

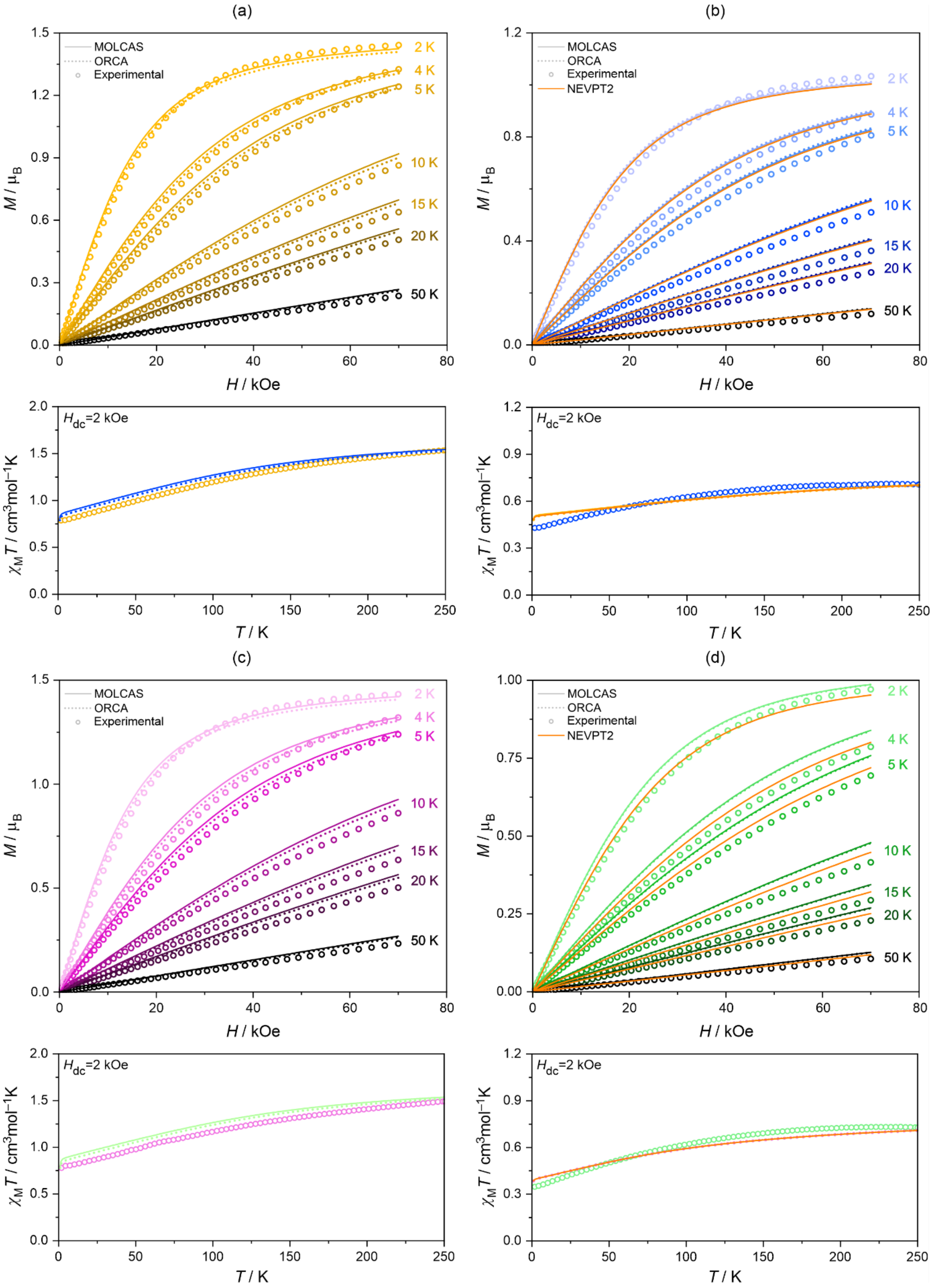

Figure S26. Direct-current (*dc*) magnetic characteristics of **1** (a), **2** (b), **3** (c), and **4** (d), including the field dependences of molar magnetization at the indicated temperatures (top parts for each compound) and the temperature dependences of the $\chi_M T$ product at the indicated *dc* field (bottom parts for each compound). Experimental results are given as colored empty circles while the solid lines represent the results of SlothPy-proceeded *ab initio* calculations obtained within the MOLCAS software (solid lines) and the ORCA (dotted lines) software. For **2** (b) and **4** (d), the analogous set of results obtained using the special NEVPT2 method of the ORCA software was also presented (orange solid lines).

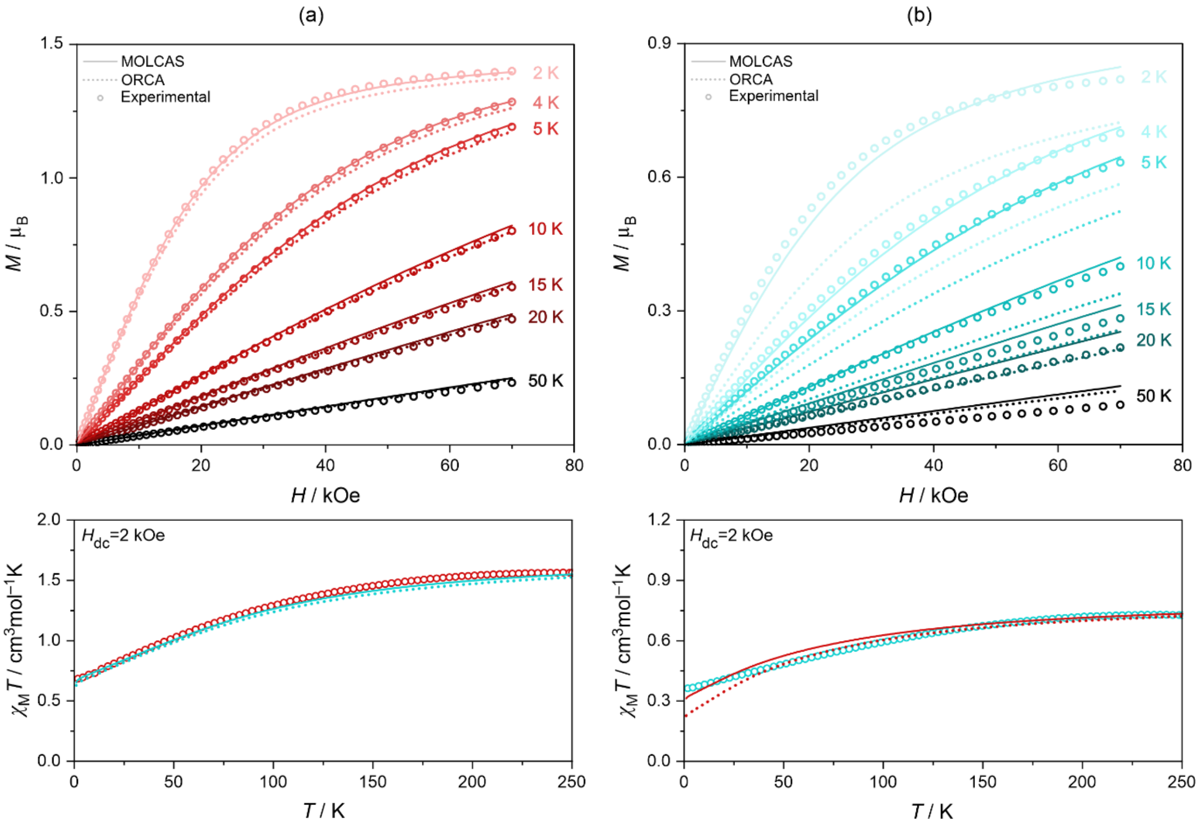

Figure S27. Direct-current (*dc*) magnetic characteristics of **5** (a) and **6** (b), including the field dependences of molar magnetization at the indicated temperatures (top parts for each compound) and the temperature dependences of the $\chi_M T$ product at the indicated *dc* field (bottom parts for each compound). Experimental results are given as colored empty circles, while the solid lines represent the results of SlothPy-proceeded *ab initio* calculations obtained within the MOLCAS software (solid lines) and the ORCA (dotted lines) software.

Comment on Figure S27. For **6** (Figure S27b), the computational results show instabilities (especially among different software) that we could not resolve. There are many possible reasons for this, beginning with an inaccurate structural model that was arbitrarily chosen from different partial occupations of distinct crystallographic positions for $[Co^{III}(CN)_5I]^{3-}$ counter ions, ending with inaccuracies in a small active space, basis or our inability to further correct the results with perturbative approach due to the inclusion of heavy I^- ions. Therefore, computational results for **6** should not be fully trusted, showing only the general view of the properties of this system.

Table S15. Supplementary information regarding performed *dc* magnetic measurements for **1**–**6** and their analysis, including employed diamagnetic corrections (determined by taking the high-temperature signal's plateau obtained from the *ab initio* calculations as a criterium) and masses of the investigated powder samples. The set of related *dc* magnetic data is presented in Figures S26 and S27.

**1**	**2**	**3**	**4**	**5**	**6**
**Diamagnetic correction / $cm^3{\cdot}mol^{-1}$**					
41	111	34	87	115	128
**Sample mass / mg**					
19.1	16.4	18.1	18.1	16.9	18.9

Table S16. Energy splitting and pseudo-*g*-tensor components of the $^{4}I_{9/2}$ ground-state term of Nd^{III} centers in **1**, shown with the compositions of ground Kramers doublets in the |m$_J$⟩ basis, calculated using MOLCAS and ORCA.

Energy and pseudo-*g*-tensor components (g_x, g_y, g_z) of 5 ground Kramers doublets calculated for compound 1 in MOLCAS				
Doublet no.	Energy / cm^{-1}	Pseudo-*g*-tensor components		
		g_x	g_y	g_z
**1.**	0.000	0.8494	1.3991	4.9660
**2.**	128.284	0.5839	2.3737	3.8966
**3.**	204.734	0.1380	1.2099	2.5604
**4.**	307.082	0.2471	2.3233	3.3877
**5.**	358.237	0.1143	2.3722	3.4199
**Composition of the five ground Kramers doublets in the |*m$_J$*⟩ basis on the "z" quantization axis within *J*=9/2 manifold (contribution over 0.1% shown) calculated for compound 1 in MOLCAS**				
**1st doublet**	**2nd doublet**	**3rd doublet**	**4th doublet**	**5th doublet**
72.7% |±9/2⟩<br>1.2% |±7/2⟩<br>9.5% |±5/2⟩<br>5.5% |±3/2⟩<br>11.1% |±1/2⟩	10.6% |±9/2⟩<br>18.5% |±7/2⟩<br>50.7% |±5/2⟩<br>16.2% |±3/2⟩<br>4.1% |±1/2⟩	1.8% |±9/2⟩<br>24.7% |±7/2⟩<br>6.4% |±5/2⟩<br>59.5% |±3/2⟩<br>7.6% |±1/2⟩	6.0% |±9/2⟩<br>27.0% |±7/2⟩<br>22.3% |±5/2⟩<br>15.1% |±3/2⟩<br>29.7% |±1/2⟩	9.0% |±9/2⟩<br>28.6% |±7/2⟩<br>11.0% |±5/2⟩<br>3.7% |±3/2⟩<br>47.6% |±1/2⟩
**Energy and pseudo-*g*-tensor components (g_x, g_y, g_z) of 5 ground Kramers doublets calculated for compound 1 in ORCA**				
Doublet no.	Energy / cm^{-1}	Pseudo-*g*-tensor components		
		g_x	g_y	g_z
**1.**	0.000	0.8560	1.4500	4.8718
**2.**	129.365	0.5689	2.3608	3.7437
**3.**	206.750	0.1425	1.1761	2.8371
**4.**	308.620	0.2452	2.4650	3.2001
**5.**	362.987	0.1408	2.2840	3.4343
**Composition of the five ground Kramers doublets in the |*m$_J$*⟩ basis on the "z" quantization axis within *J*=9/2 manifold (contribution over 0.1% shown) calculated for compound 1 in ORCA**				
**1st doublet**	**2nd doublet**	**3rd doublet**	**4th doublet**	**5th doublet**
70.9% |±9/2⟩<br>2.2% |±7/2⟩<br>9.8% |±5/2⟩<br>5.7% |±3/2⟩<br>11.4% |±1/2⟩	11.9% |±9/2⟩<br>18.3% |±7/2⟩<br>47.5% |±5/2⟩<br>17.0% |±3/2⟩<br>5.3% |±1/2⟩	1.3% |±9/2⟩<br>27.1% |±7/2⟩<br>6.6% |±5/2⟩<br>55.5% |±3/2⟩<br>9.5% |±1/2⟩	7.0% |±9/2⟩<br>24.1% |±7/2⟩<br>22.8% |±5/2⟩<br>17.8% |±3/2⟩<br>28.3% |±1/2⟩	8.9% |±9/2⟩<br>28.3% |±7/2⟩<br>13.3% |±5/2⟩<br>4.0% |±3/2⟩<br>45.5% |±1/2⟩

Table S17. Energy splitting and pseudo-*g*-tensor components of the $^2F_{5/2}$ ground-state term of Ce^{III} centers in **2**, shown with the compositions of ground Kramers doublets in the $|m_J\rangle$ basis, calculated using MOLCAS and ORCA.

Energy and pseudo-*g*-tensor components (g_x, g_y, g_z) of 3 ground Kramers doublets calculated for compound 2 in MOLCAS				
Doublet no.	Energy / cm^{-1}	Pseudo-*g*-tensor components		
		g_x	g_y	g_z
**1.**	0.000	0.4091	0.6140	3.9656
**2.**	298.254	0.1454	0.6834	2.9586
**3.**	573.824	0.6908	1.6867	3.0619

Composition of the three ground Kramers doublets in the $\|m_J\rangle$ basis on the "z" quantization axis within *J*=5/2 manifold (contribution over 0.1% shown) calculated for compound 2 in MOLCAS		
1st doublet	2nd doublet	3rd doublet
97.2% $\|\pm5/2\rangle$<br>2.1% $\|\pm3/2\rangle$<br>0.7% $\|\pm1/2\rangle$	1.5% $\|\pm5/2\rangle$<br>72.4% $\|\pm3/2\rangle$<br>26.1% $\|\pm1/2\rangle$	1.3% $\|\pm5/2\rangle$<br>25.5% $\|\pm3/2\rangle$<br>73.2% $\|\pm1/2\rangle$

Energy and pseudo-*g*-tensor components (g_x, g_y, g_z) of 3 ground Kramers doublets calculated for compound 2 in ORCA				
Doublet no.	Energy / cm^{-1}	Pseudo-*g*-tensor components		
		g_x	g_y	g_z
**1.**	0.000	0.4392	0.6354	3.9327
**2.**	281.734	0.1114	0.6679	3.0113
**3.**	545.176	0.6852	1.6733	3.0437

Composition of the three ground Kramers doublets in the $\|m_J\rangle$ basis on the "z" quantization axis within *J*=5/2 manifold (contribution over 0.1% shown) calculated for compound 2 in ORCA		
1st doublet	2nd doublet	3rd doublet
96.9% $\|\pm5/2\rangle$<br>2.3% $\|\pm3/2\rangle$<br>0.8% $\|\pm1/2\rangle$	1.7% $\|\pm5/2\rangle$<br>69.5% $\|\pm3/2\rangle$<br>28.8% $\|\pm1/2\rangle$	1.4% $\|\pm5/2\rangle$<br>28.2% $\|\pm3/2\rangle$<br>70.4% $\|\pm1/2\rangle$

Table S18. Energy splitting and pseudo-*g*-tensor components of the $^4I_{9/2}$ ground-state term of Nd^{III} centers in **3**, shown with the compositions of ground Kramers doublets in the |m$_J$⟩ basis, calculated using MOLCAS and ORCA.

Energy and pseudo-*g*-tensor components (g_x, g_y, g_z) of 5 ground Kramers doublets calculated for compound 3 in MOLCAS				
Doublet no.	Energy / cm^{-1}	Pseudo-*g*-tensor components		
		g_x	g_y	g_z
**1.**	0.000	0.6696	1.3177	5.0727
**2.**	132.154	0.7398	1.2839	4.2914
**3.**	212.647	0.9492	1.8297	2.8224
**4.**	303.591	0.7443	1.6379	4.2120
**5.**	384.313	0.1890	2.1356	4.1135
**Composition of the five ground Kramers doublets in the |*m$_J$*⟩ basis on the "z" quantization axis within *J*=9/2 manifold (contribution over 0.1% shown) calculated for compound 3 in MOLCAS**				
**1st doublet**	**2nd doublet**	**3rd doublet**	**4th doublet**	**5th doublet**
74.6% |±9/2⟩<br>0.8% |±7/2⟩<br>10.0% |±5/2⟩<br>3.0% |±3/2⟩<br>11.6% |±1/2⟩	6.7% |±9/2⟩<br>5.7% |±7/2⟩<br>45.3% |±5/2⟩<br>36.8% |±3/2⟩<br>5.5% |±1/2⟩	5.0% |±9/2⟩<br>24.4% |±7/2⟩<br>27.1% |±5/2⟩<br>40.9% |±3/2⟩<br>2.6% |±1/2⟩	3.3% |±9/2⟩<br>48.7% |±7/2⟩<br>14.1% |±5/2⟩<br>14.8% |±3/2⟩<br>19.1% |±1/2⟩	10.3% |±9/2⟩<br>20.4% |±7/2⟩<br>3.5% |±5/2⟩<br>4.5% |±3/2⟩<br>61.3% |±1/2⟩
**Energy and pseudo-*g*-tensor components (g_x, g_y, g_z) of 5 ground Kramers doublets calculated for compound 3 in ORCA**				
Doublet no.	Energy / cm^{-1}	Pseudo-*g*-tensor components		
		g_x	g_y	g_z
**1.**	0.000	0.7098	1.4126	4.9463
**2.**	131.836	0.7647	1.1810	4.2075
**3.**	211.715	0.8101	1.7796	3.0190
**4.**	308.519	0.7661	1.6761	4.1599
**5.**	386.894	0.1807	2.1027	4.0664
**Composition of the five ground Kramers doublets in the |*m$_J$*⟩ basis on the "z" quantization axis within *J*=9/2 manifold (contribution over 0.1% shown) calculated for compound 3 in ORCA**				
**1st doublet**	**2nd doublet**	**3rd doublet**	**4th doublet**	**5th doublet**
73.0% |±9/2⟩<br>0.8% |±7/2⟩<br>10.7% |±5/2⟩<br>3.3% |±3/2⟩<br>12.2% |±1/2⟩	7.4% |±9/2⟩<br>5.0% |±7/2⟩<br>42.9% |±5/2⟩<br>38.5% |±3/2⟩<br>6.2% |±1/2⟩	5.2% |±9/2⟩<br>26.0% |±7/2⟩<br>28.0% |±5/2⟩<br>38.5% |±3/2⟩<br>2.2% |±1/2⟩	3.6% |±9/2⟩<br>46.4% |±7/2⟩<br>14.2% |±5/2⟩<br>15.3% |±3/2⟩<br>20.6% |±1/2⟩	10.8% |±9/2⟩<br>21.8% |±7/2⟩<br>4.2% |±5/2⟩<br>4.4% |±3/2⟩<br>58.8% |±1/2⟩

Table S19. Energy splitting and pseudo-*g*-tensor components of the $^2F_{5/2}$ ground-state term of Ce^{III} centers in **4**, shown with the compositions of ground Kramers doublets in the |m$_J$⟩ basis, calculated using MOLCAS and ORCA.

Energy and pseudo-*g*-tensor components (g_x, g_y, g_z) of 3 ground Kramers doublets calculated for compound 4 in MOLCAS				
Doublet no.	Energy / cm^{-1}	Pseudo-*g*-tensor components		
		g_x	g_y	g_z
**1.**	0.000	1.1045	1.4093	3.0674
**2.**	181.446	0.3058	1.3755	2.7544
**3.**	462.412	0.3207	0.7904	3.6895

Composition of the three ground Kramers doublets in the |m_J⟩ basis on the "z" quantization axis within *J*=5/2 manifold (contribution over 0.1% shown) calculated for compound 4 in MOLCAS		
1st doublet	2nd doublet	3rd doublet
79.7% |±5/2⟩<br>13.4% |±3/2⟩<br>6.9% |±1/2⟩	14.8% |±5/2⟩<br>43.5% |±3/2⟩<br>41.7% |±1/2⟩	5.6% |±5/2⟩<br>43.1% |±3/2⟩<br>51.3% |±1/2⟩

Energy and pseudo-*g*-tensor components (g_x, g_y, g_z) of 3 ground Kramers doublets calculated for compound 4 in ORCA				
Doublet no.	Energy / cm^{-1}	Pseudo-*g*-tensor components		
		g_x	g_y	g_z
**1.**	0.000	1.1484	1.3822	3.0567
**2.**	178.546	0.2248	1.4240	2.7420
**3.**	453.492	0.3620	0.8969	3.6250

Composition of the three ground Kramers doublets in the |m_J⟩ basis on the "z" quantization axis within *J*=5/2 manifold (contribution over 0.1% shown) calculated for compound 4 in ORCA		
1st doublet	2nd doublet	3rd doublet
80.2% |±5/2⟩<br>13.9% |±3/2⟩<br>5.9% |±1/2⟩	14.8% |±5/2⟩<br>42.7% |±3/2⟩<br>42.4% |±1/2⟩	5.0% |±5/2⟩<br>43.4% |±3/2⟩<br>51.6% |±1/2⟩

Table S20. Energy splitting and pseudo-g-tensor components of the $^4I_{9/2}$ ground-state term of Nd^{III} centers in **5**, shown with the compositions of ground Kramers doublets in the $|m_J\rangle$ basis, calculated using MOLCAS and ORCA.

Energy and pseudo-g-tensor components (g_x, g_y, g_z) of 5 ground Kramers doublets calculated for compound 5 in MOLCAS				
Doublet no.	Energy / cm^{-1}	Pseudo-g-tensor components		
		g_x	g_y	g_z
**1.**	0.000	1.7894	2.5854	3.4006
**2.**	114.472	0.6927	2.0887	3.1769
**3.**	144.831	0.4534	0.7417	3.6048
**4.**	253.702	0.4530	1.3989	4.7947
**5.**	325.030	1.5952	2.6005	3.2617
**Composition of the five ground Kramers doublets in the $|m_J\rangle$ basis on the "z" quantization axis within J=9/2 manifold (contribution over 0.1% shown) calculated for compound 5 in MOLCAS**				
**1st doublet**	**2nd doublet**	**3rd doublet**	**4th doublet**	**5th doublet**
33.1% $|\pm9/2\rangle$<br>19.8% $|\pm7/2\rangle$<br>26.2% $|\pm5/2\rangle$<br>17.2% $|\pm3/2\rangle$<br>3.7% $|\pm1/2\rangle$	19.5% $|\pm9/2\rangle$<br>5.1% $|\pm7/2\rangle$<br>39.7% $|\pm5/2\rangle$<br>14.7% $|\pm3/2\rangle$<br>21.0% $|\pm1/2\rangle$	20.7% $|\pm9/2\rangle$<br>7.8% $|\pm7/2\rangle$<br>9.8% $|\pm5/2\rangle$<br>43.3% $|\pm3/2\rangle$<br>18.4% $|\pm1/2\rangle$	14.1% $|\pm9/2\rangle$<br>25.6% $|\pm7/2\rangle$<br>7.6% $|\pm5/2\rangle$<br>17.4% $|\pm3/2\rangle$<br>35.4% $|\pm1/2\rangle$	12.6% $|\pm9/2\rangle$<br>41.7% $|\pm7/2\rangle$<br>16.7% $|\pm5/2\rangle$<br>7.5% $|\pm3/2\rangle$<br>21.5% $|\pm1/2\rangle$
**Energy and pseudo-g-tensor components (g_x, g_y, g_z) of 5 ground Kramers doublets calculated for compound 5 in ORCA**				
Doublet no.	Energy / cm^{-1}	Pseudo-g-tensor components		
		g_x	g_y	g_z
**1.**	0.000	1.7621	2.6596	3.2231
**2.**	100.775	0.4253	2.1699	3.1768
**3.**	151.889	0.3383	1.2937	3.3040
**4.**	255.130	0.3123	1.5558	4.3534
**5.**	325.736	1.5037	2.5520	3.2749
**Composition of the five ground Kramers doublets in the $|m_J\rangle$ basis on the "z" quantization axis within J=9/2 manifold (contribution over 0.1% shown) calculated for compound 5 in ORCA**				
**1st doublet**	**2nd doublet**	**3rd doublet**	**4th doublet**	**5th doublet**
4.6% $|\pm9/2\rangle$<br>70.0% $|\pm7/2\rangle$<br>19.6% $|\pm5/2\rangle$<br>4.3% $|\pm3/2\rangle$<br>1.5% $|\pm1/2\rangle$	31.3% $|\pm9/2\rangle$<br>1.4% $|\pm7/2\rangle$<br>6.3% $|\pm5/2\rangle$<br>20.2% $|\pm3/2\rangle$<br>40.7% $|\pm1/2\rangle$	21.2% $|\pm9/2\rangle$<br>5.1% $|\pm7/2\rangle$<br>7.7% $|\pm5/2\rangle$<br>19.3% $|\pm3/2\rangle$<br>46.8% $|\pm1/2\rangle$	15.7% $|\pm9/2\rangle$<br>13.9% $|\pm7/2\rangle$<br>30.1% $|\pm5/2\rangle$<br>33.7% $|\pm3/2\rangle$<br>6.5% $|\pm1/2\rangle$	27.2% $|\pm9/2\rangle$<br>9.7% $|\pm7/2\rangle$<br>36.2% $|\pm5/2\rangle$<br>22.5% $|\pm3/2\rangle$<br>4.5% $|\pm1/2\rangle$

Table S21. Energy splitting and pseudo-*g*-tensor components of the $^2F_{5/2}$ ground-state term of Ce^{III} centers in **6**, shown with the compositions of ground Kramers doublets in the $|m_J\rangle$ basis, calculated using MOLCAS and ORCA.

Energy and pseudo-*g*-tensor components (g_x, g_y, g_z) of 3 ground Kramers doublets calculated for compound 6 in MOLCAS				
Doublet no.	Energy / cm^{-1}	Pseudo-*g*-tensor components		
		g_x	g_y	g_z
**1.**	0.000	0.2239	0.9208	3.0237
**2.**	72.577	1.5222	1.6407	2.1248
**3.**	256.357	0.3381	0.9452	2.6283

Composition of the three ground Kramers doublets in the $|m_J\rangle$ basis on the "z" quantization axis within *J*=5/2 manifold (contribution over 0.1% shown) calculated for compound 6 in MOLCAS		
1st doublet	2nd doublet	3rd doublet
73.8% $|\pm5/2\rangle$<br>3.3% $|\pm3/2\rangle$<br>22.8% $|\pm1/2\rangle$	20.3% $|\pm5/2\rangle$<br>18.1% $|\pm3/2\rangle$<br>61.6% $|\pm1/2\rangle$	5.8% $|\pm5/2\rangle$<br>78.6% $|\pm3/2\rangle$<br>15.6% $|\pm1/2\rangle$

Energy and pseudo-*g*-tensor components (g_x, g_y, g_z) of 3 ground Kramers doublets calculated for compound 6 in ORCA				
Doublet no.	Energy / cm^{-1}	Pseudo-*g*-tensor components		
		g_x	g_y	g_z
**1.**	0.000	0.0243	0.8215	2.5506
**2.**	70.111	0.6542	1.5016	2.4185
**3.**	253.899	0.4144	1.0612	2.4246

Composition of the three ground Kramers doublets in the $|m_J\rangle$ basis on the "z" quantization axis within *J*=5/2 manifold (contribution over 0.1% shown) calculated for compound 6 in ORCA		
1st doublet	2nd doublet	3rd doublet
46.6% $|\pm5/2\rangle$<br>25.6% $|\pm3/2\rangle$<br>27.8% $|\pm1/2\rangle$	39.4% $|\pm5/2\rangle$<br>23.3% $|\pm3/2\rangle$<br>37.3% $|\pm1/2\rangle$	14.0% $|\pm5/2\rangle$<br>51.1% $|\pm3/2\rangle$<br>35.0% $|\pm1/2\rangle$

Table S22. Energy splitting and pseudo-*g*-tensor components of the $^2F_{5/2}$ ground-state term of Ce^{III} centers in compounds **2** and **4**, shown with the compositions of ground Kramers doublets in the |m_J⟩ basis, calculated using NEVPT2 method within ORCA program.

Energy and pseudo-*g*-tensor components (g_x, g_y, g_z) of 3 ground Kramers doublets calculated for compound 2 using the NEVPT2 method in ORCA				
Doublet no.	Energy / cm^{-1}	Pseudo-*g*-tensor components		
		g_x	g_y	g_z
**1.**	0.000	0.4155	0.6380	3.9176
**2.**	296.678	0.1055	0.6593	3.0060
**3.**	560.963	0.6753	1.6792	3.0353

Composition of the three ground Kramers doublets in the |m_J⟩ basis on the "z" quantization axis within *J*=5/2 manifold (contribution over 0.1% shown) calculated for compound 2 using the NEVPT2 method in ORCA		
1st doublet	2nd doublet	3rd doublet
96.9% |±5/2⟩<br>2.2% |±3/2⟩<br>0.9% |±1/2⟩	1.8% |±5/2⟩<br>70.3% |±3/2⟩<br>27.9% |±1/2⟩	1.3% |±5/2⟩<br>27.5% |±3/2⟩<br>71.2% |±1/2⟩

Energy and pseudo-*g*-tensor components (g_x, g_y, g_z) of 3 ground Kramers doublets calculated for compound 4 using the NEVPT2 method in ORCA				
Doublet no.	Energy / cm^{-1}	Pseudo-*g*-tensor components		
		g_x	g_y	g_z
**1.**	0.000	1.0952	1.4673	2.8824
**2.**	194.334	0.2438	1.3336	2.7879
**3.**	474.688	0.3715	0.9472	3.5953

Composition of the three ground Kramers doublets in the |m_J⟩ basis on the "z" quantization axis within *J*=5/2 manifold (contribution over 0.1% shown) calculated for compound 4 using the NEVPT2 method in ORCA		
1st doublet	2nd doublet	3rd doublet
76.1% |±5/2⟩<br>14.9% |±3/2⟩<br>9.0% |±1/2⟩	17.1% |±5/2⟩<br>34.4% |±3/2⟩<br>48.6% |±1/2⟩	6.8% |±5/2⟩<br>50.7% |±3/2⟩<br>42.5% |±1/2⟩

(a)

(b)

(c)

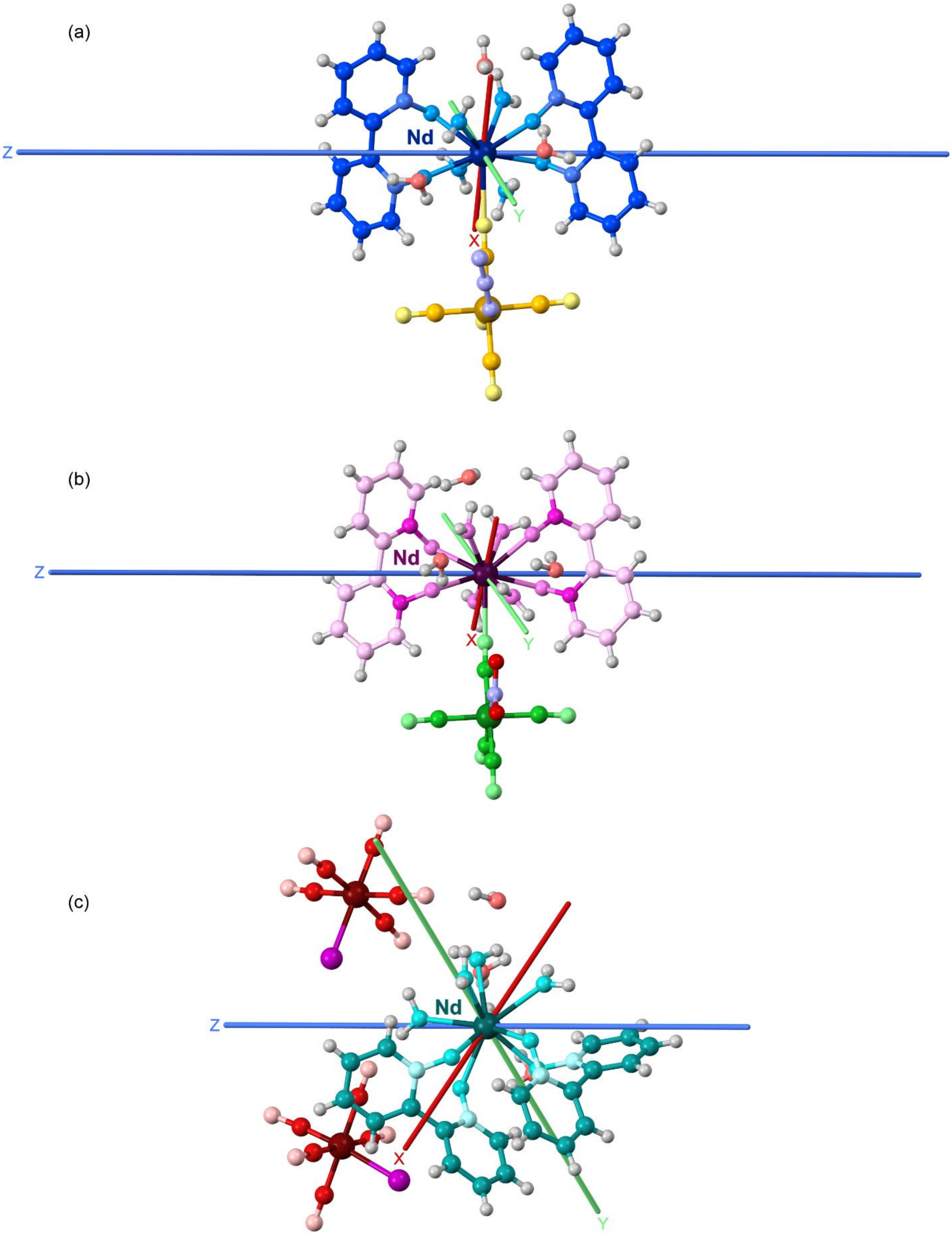

Figure S28. Fragments of the crystal structures containing the magnetic Nd(III) center of **1** (a), **3** (b), and **5** (c), which were used for the *ab initio* calculations shown with the determined main magnetic axes marked with red (X-axis), green (Y-axis), and blue (Z-axis). Lengths of these axes are directly related to the g_x, g_y, and g_z components of the pseudo-*g*-tensor of the ground Kramers doublet (see Tables S16, S18, and S20).

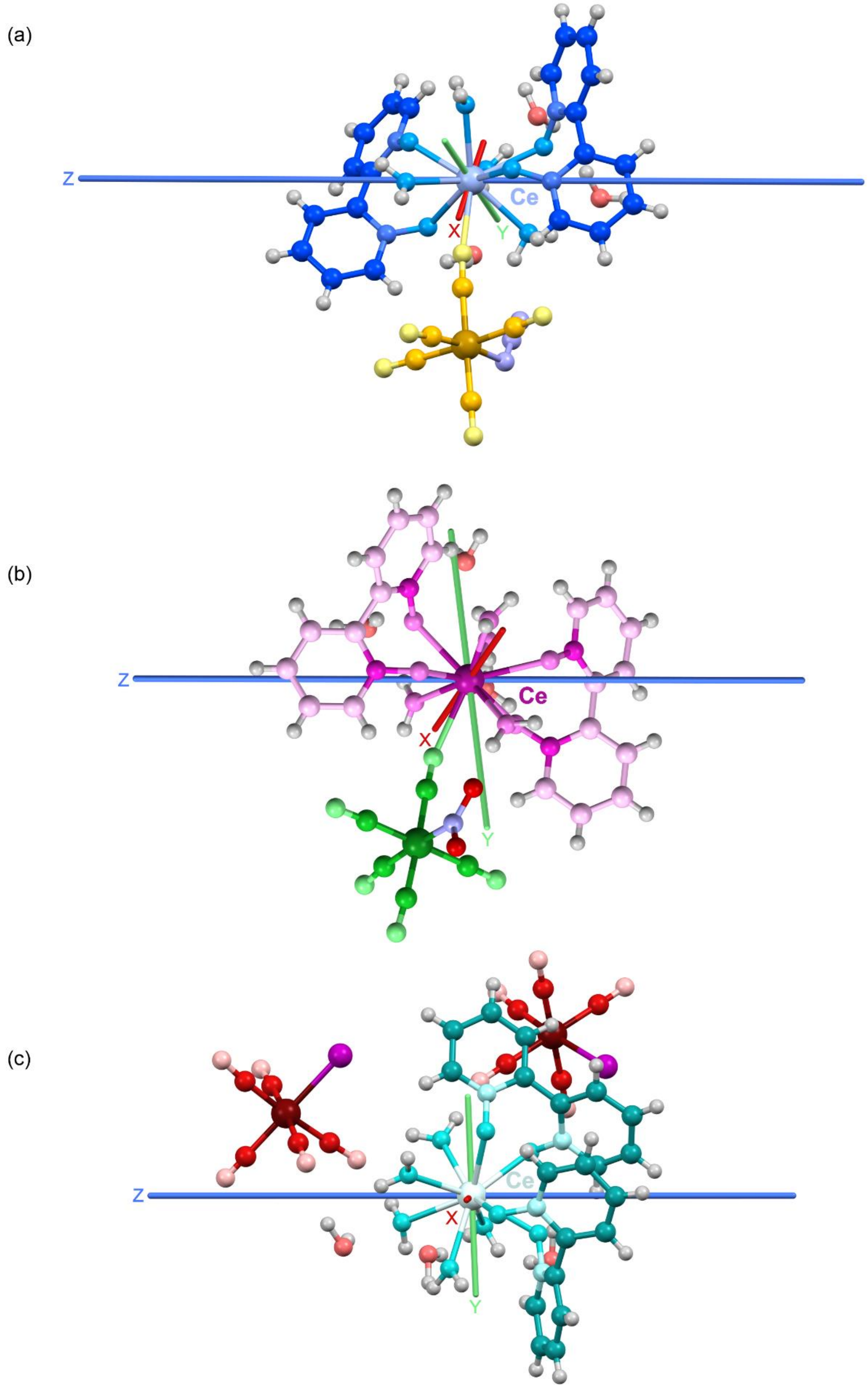

Figure S29. Fragments of the crystal structures containing the magnetic Ce(III) center of **2** (a), **4** (b), and **6** (c), which were used for the *ab initio* calculations shown with the determined main magnetic axes marked with red (X-axis), green (Y-axis), and blue (Z-axis). Lengths of these axes are directly related to the g_x, g_y, and g_z components of the pseudo-*g*-tensor of the ground Kramers doublet (see Tables S17, S19, and S21).

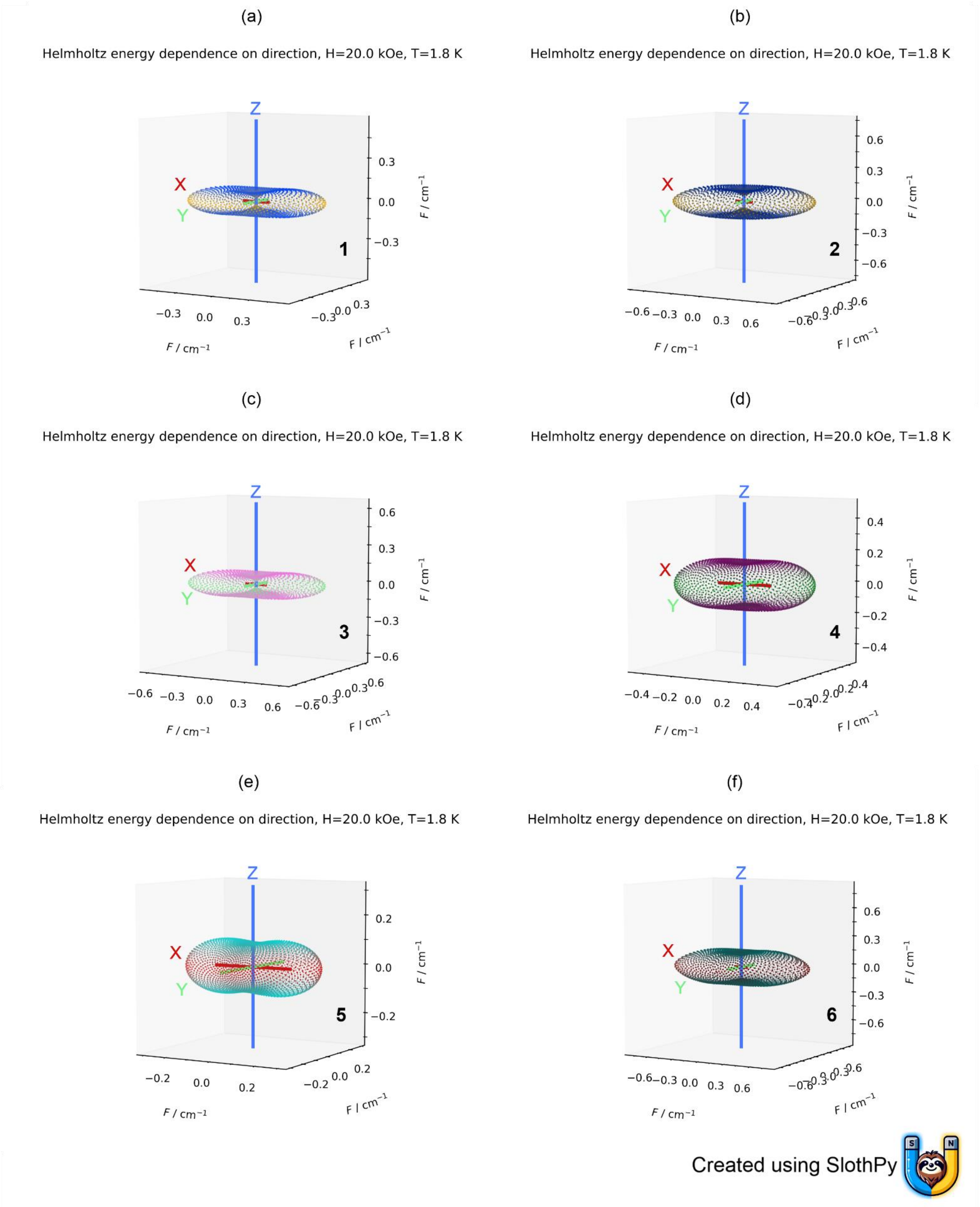

Figure S30. Dependences of Helmholtz energy of **1** (a), **2** (b), **3** (c), **4** (d), **5** (e), and **6** (f) on the direction of applied *dc* magnetic field of H = 20 kOe at T = 1.8 K, presented together with the main magnetic axes of the ground Kramers doublets, all simulated using the SlothPy software (using Matplotlib as a plotting library)[S13] from the results of *ab initio* calculations.

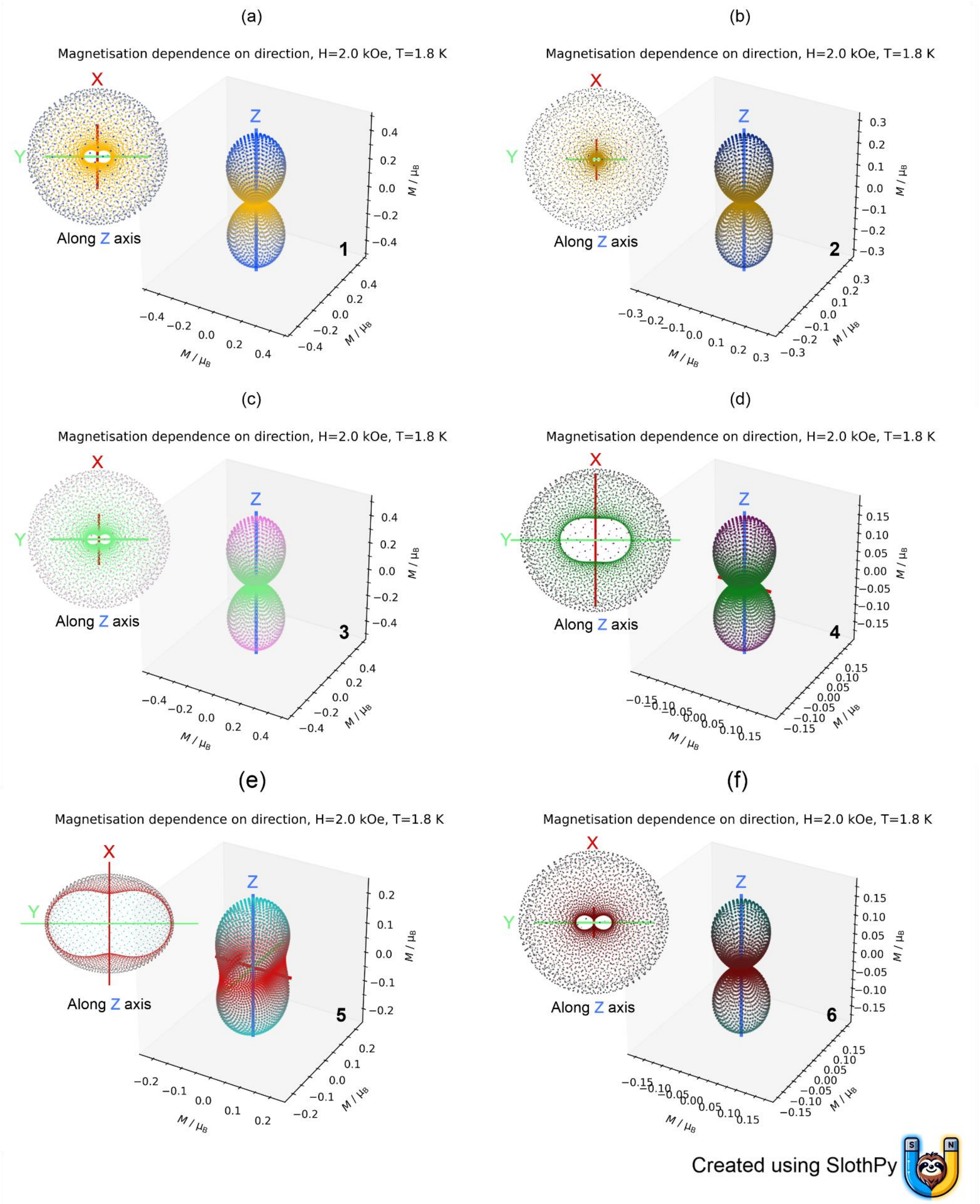

Figure S31. Dependences of magnetization of **1** (a), **2** (b), **3** (c), **4** (d), **5** (e), and **6** (f) on the direction of applied *dc* magnetic field of H = 2.0 kOe at T = 1.8 K, presented together with the main magnetic axes of the ground Kramers doublets, all simulated using the SlothPy software (using Matplotlib as a plotting library)[S13] from the results of *ab initio* calculations. The magnified cross-sections of the view through the Z-axis are additionally shown in the upper left corner for each compound.

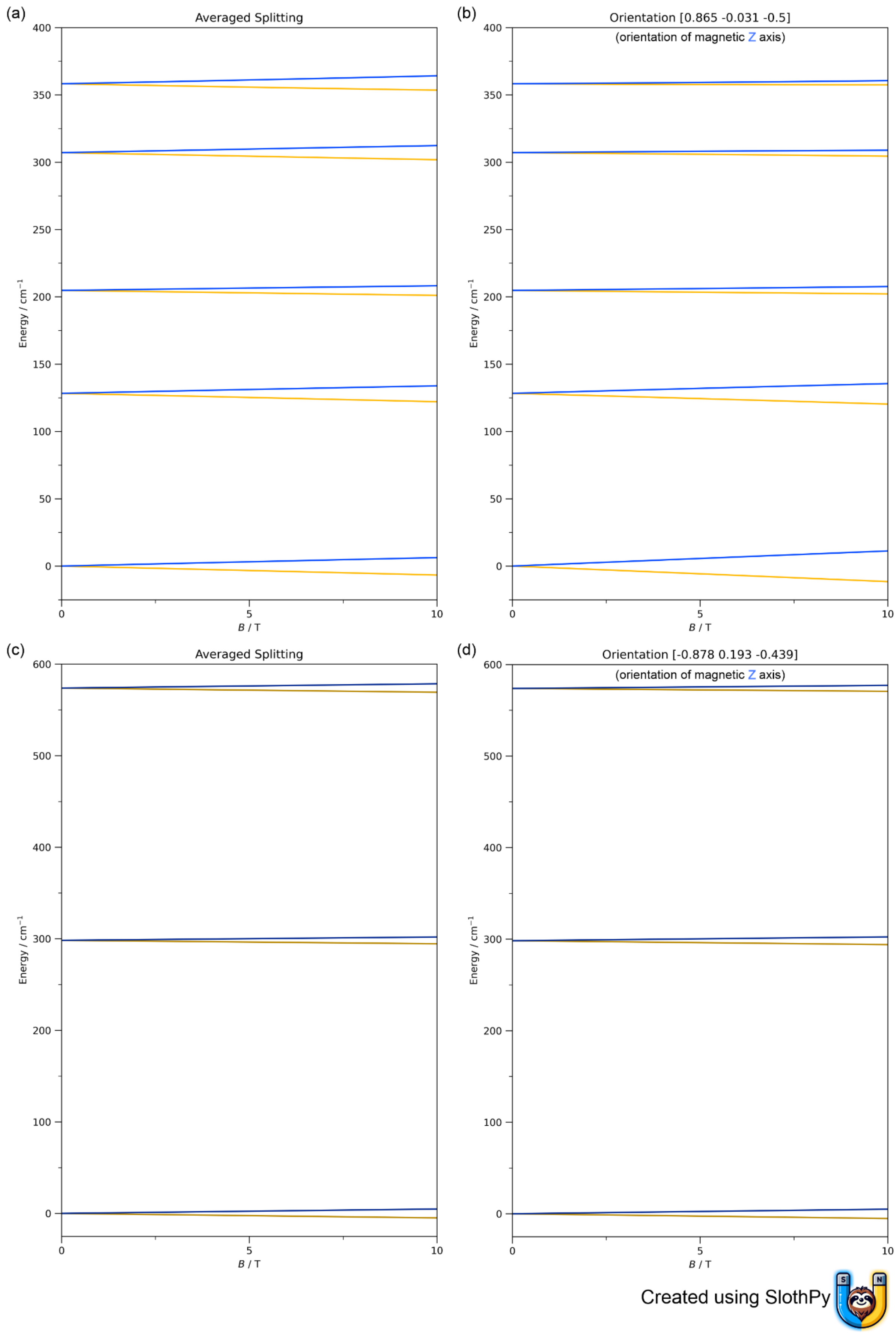

Figure S32. Zeeman splitting of Kramers doublets within the ground multiplets in the function of magnetic field strength in the form averaged over the grid (a and c) and applied along the orientation of Z main magnetic axis (b and d). Parts (a) and (b) correspond to compound **1** while parts (c) and (d) to compound **2**. The graphs were created using SlothPy (based on the results of *ab initio* calculations), which uses Matplotlib as a plotting library.[S13]

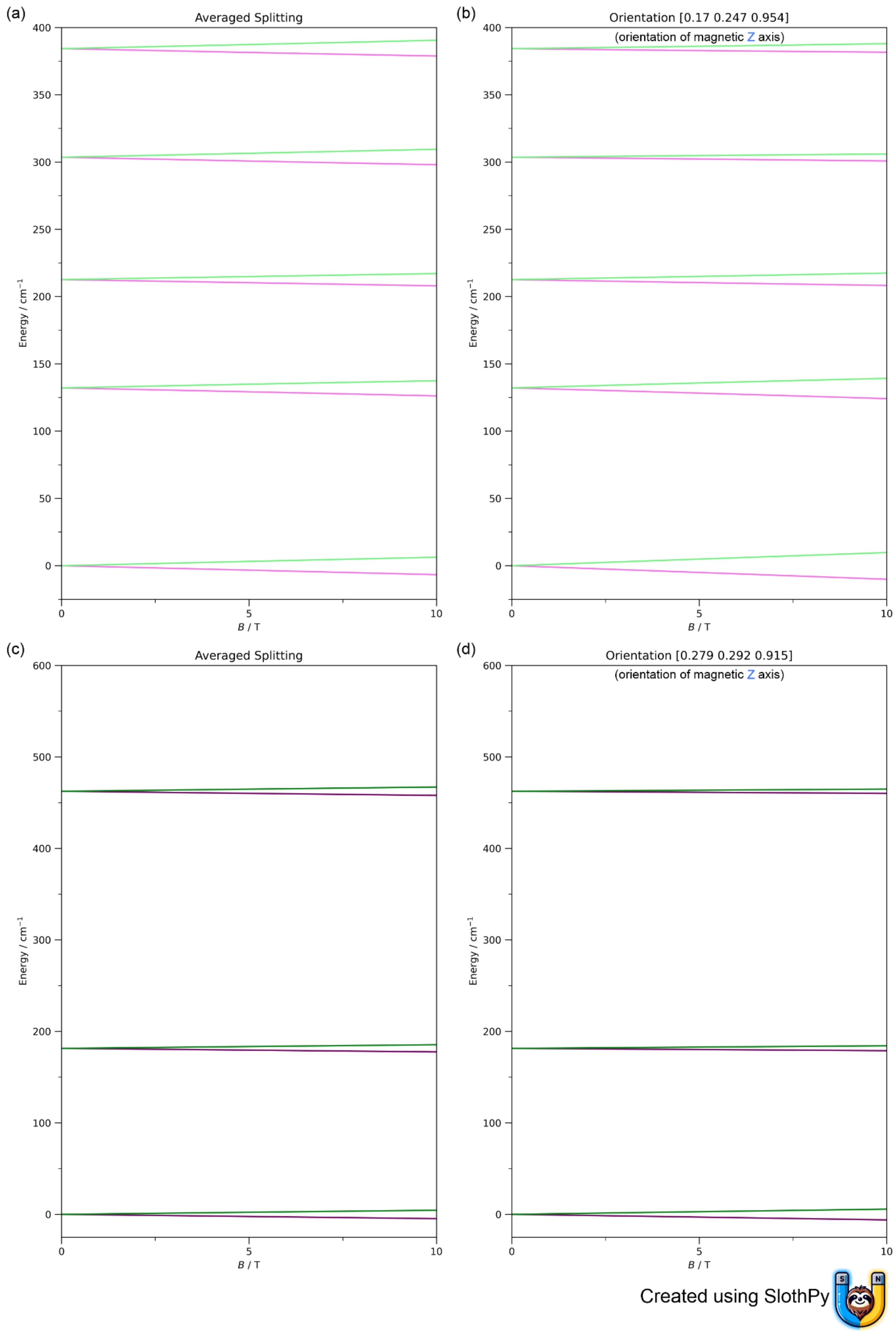

Figure S33. Zeeman splitting of Kramers doublets within the ground multiplets in the function of magnetic field strength in the form averaged over the grid (a and c) and applied along the orientation of Z main magnetic axis (b and d). Parts (a) and (b) correspond to compound **3** while parts (c) and (d) to compound **4**. The graphs were created using SlothPy (based on the results of *ab initio* calculations), which uses Matplotlib as a plotting library.[S13]

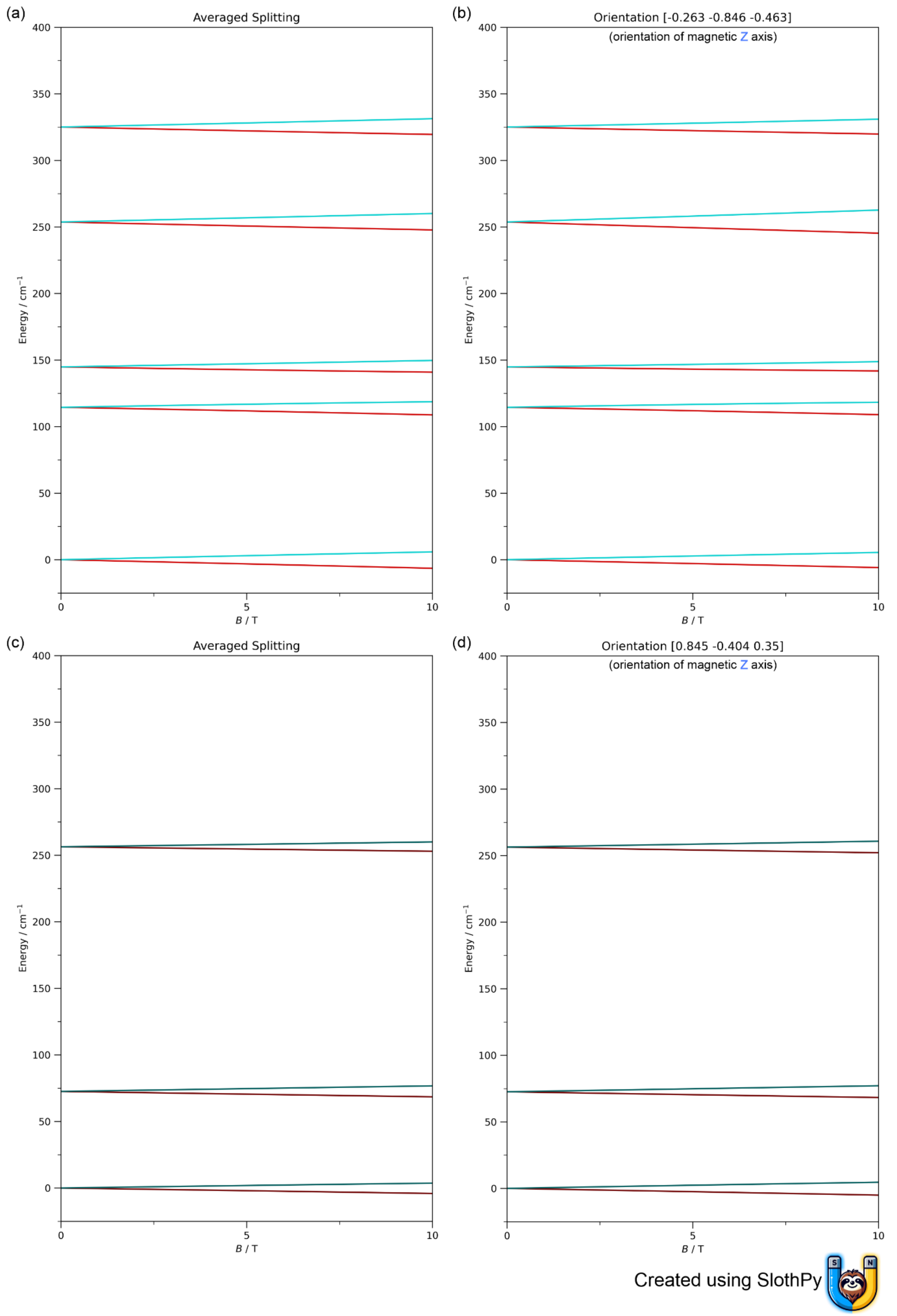

Figure S34. Zeeman splitting of Kramers doublets within the ground multiplets in the function of magnetic field strength in the form averaged over the grid (a and c) and applied along the orientation of Z main magnetic axis (b and d). Parts (a) and (b) correspond to compound **5** while parts (c) and (d) to compound **6**. The graphs were created using SlothPy (based on the results of *ab initio* calculations), which uses Matplotlib as a plotting library.[S13]

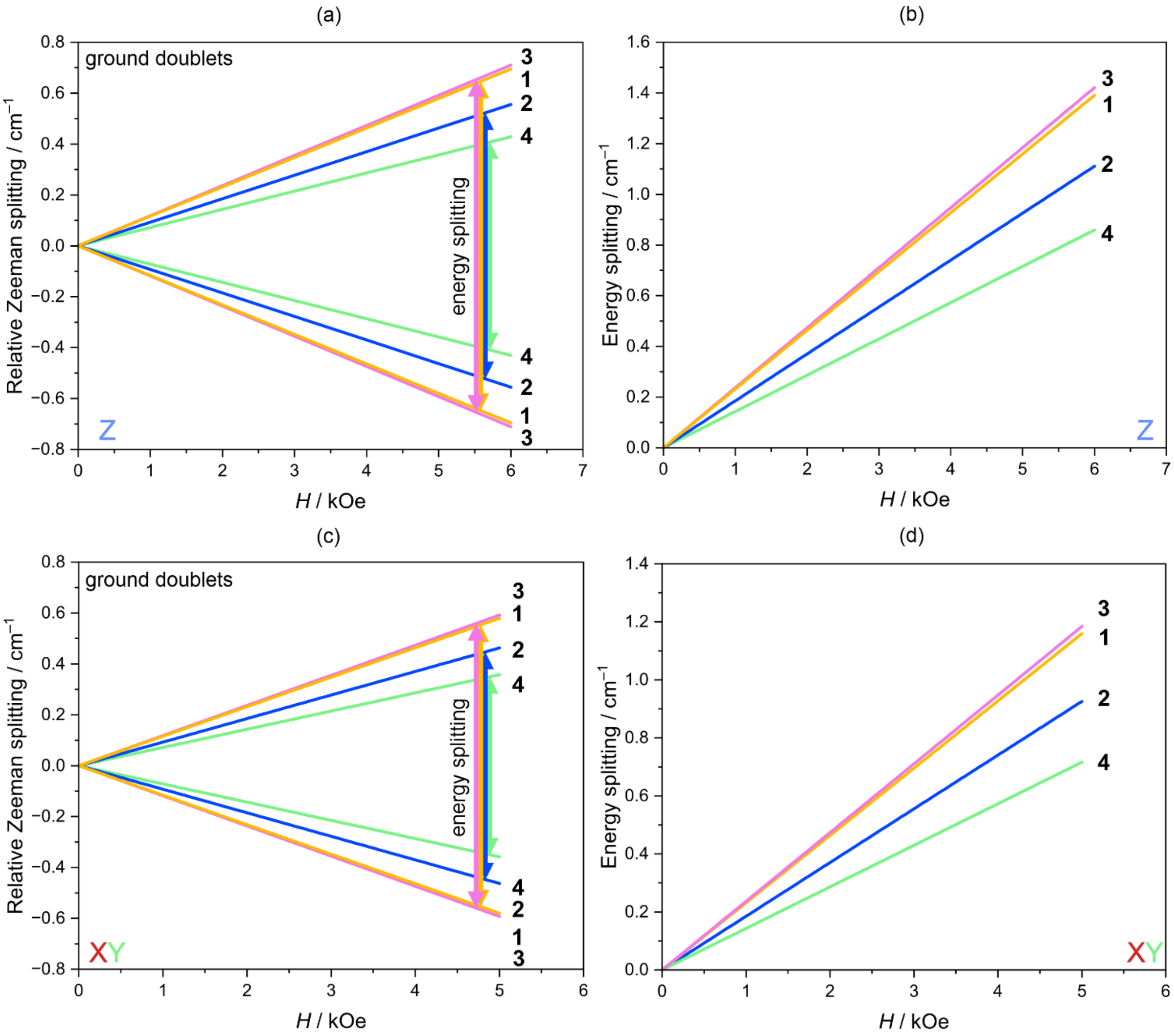

Figure S35. Zeeman splitting of the ground Kramers doublet in the function of the magnitude of the magnetic field, *H*, applied in the indicated range in the direction of Z (a) and X+Y magnetic axes (c) for compounds **1**–**4**, and the visualization of the related changes in energy difference within the doublet (b and d, for Z and X+Y axes, respectively).

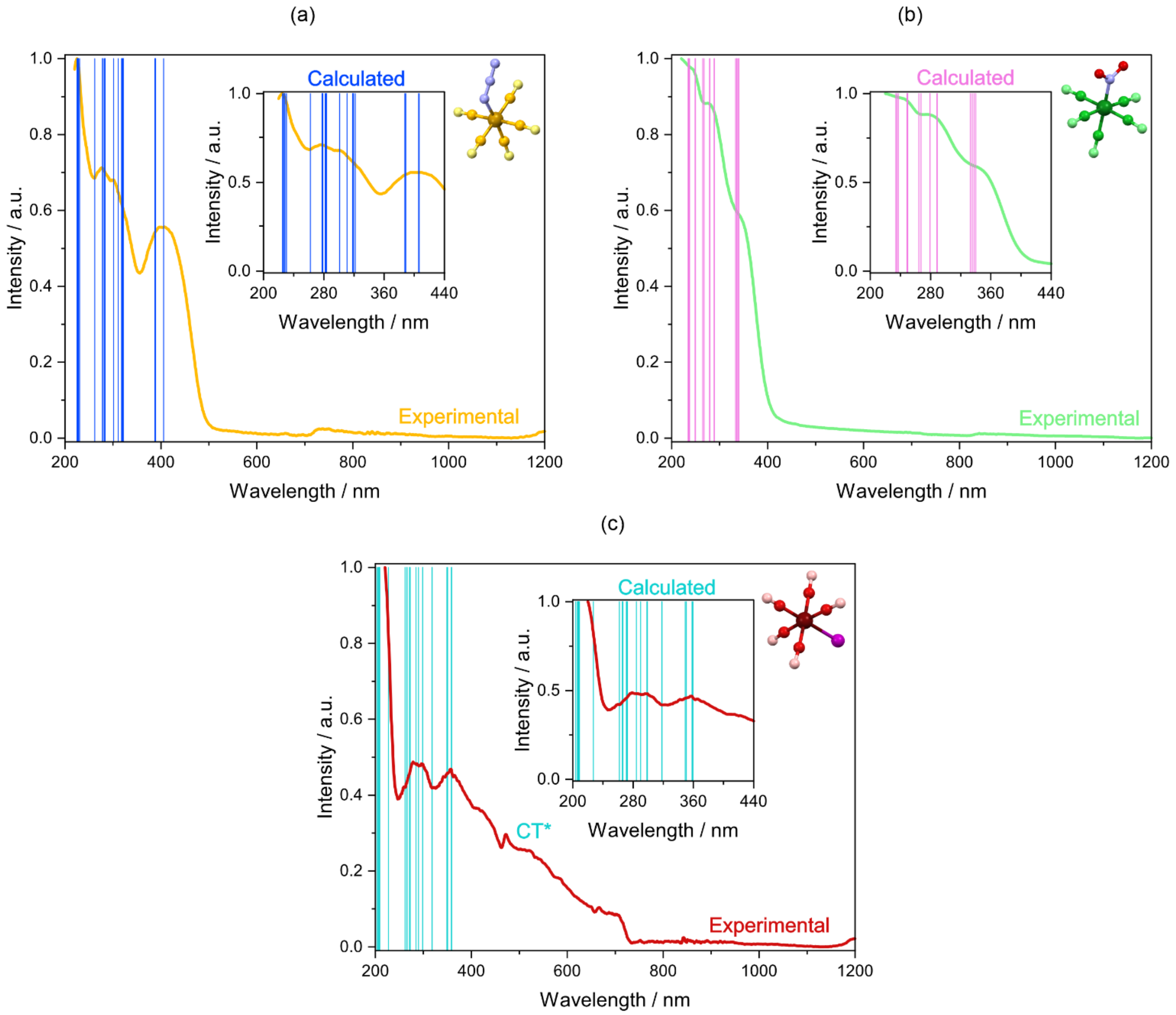

Figure S36. *Ab initio* (CASSCF/NEVPT2) calculated wavelengths (colored vertical lines) of light absorption bands for Co^{3+} ions in $[Co^{III}(CN)_5(N_3)]^{3-}$ complexes of **1** (a), $[Co^{III}(CN)_5(NO_2)]^{3-}$ complexes of **2** (b), and $[Co^{III}(CN)_5I]^{3-}$ complexes of **3**, compared with the experimental solid-state UV-vis-NIR absorption spectra (indicated colored lines) of the precursor complexes (potassium salts, see Experimental Details; the analogous comparison with the spectra of the compounds is presented in Figure S37). The fragments used for these calculations (Co(III) complexes) are shown in the upper right corner of each part of the figure. All the spectra were normalized. In part (c), the charge-transfer band (CT*) was indicated; this assignment was made based on the lack of the related electronic transitions of the purely Co(III) origin in this region (i.e., the lack of *ab initio* calculated absorption bands in this region as they represent the Co(III) d-d electronic transitions).

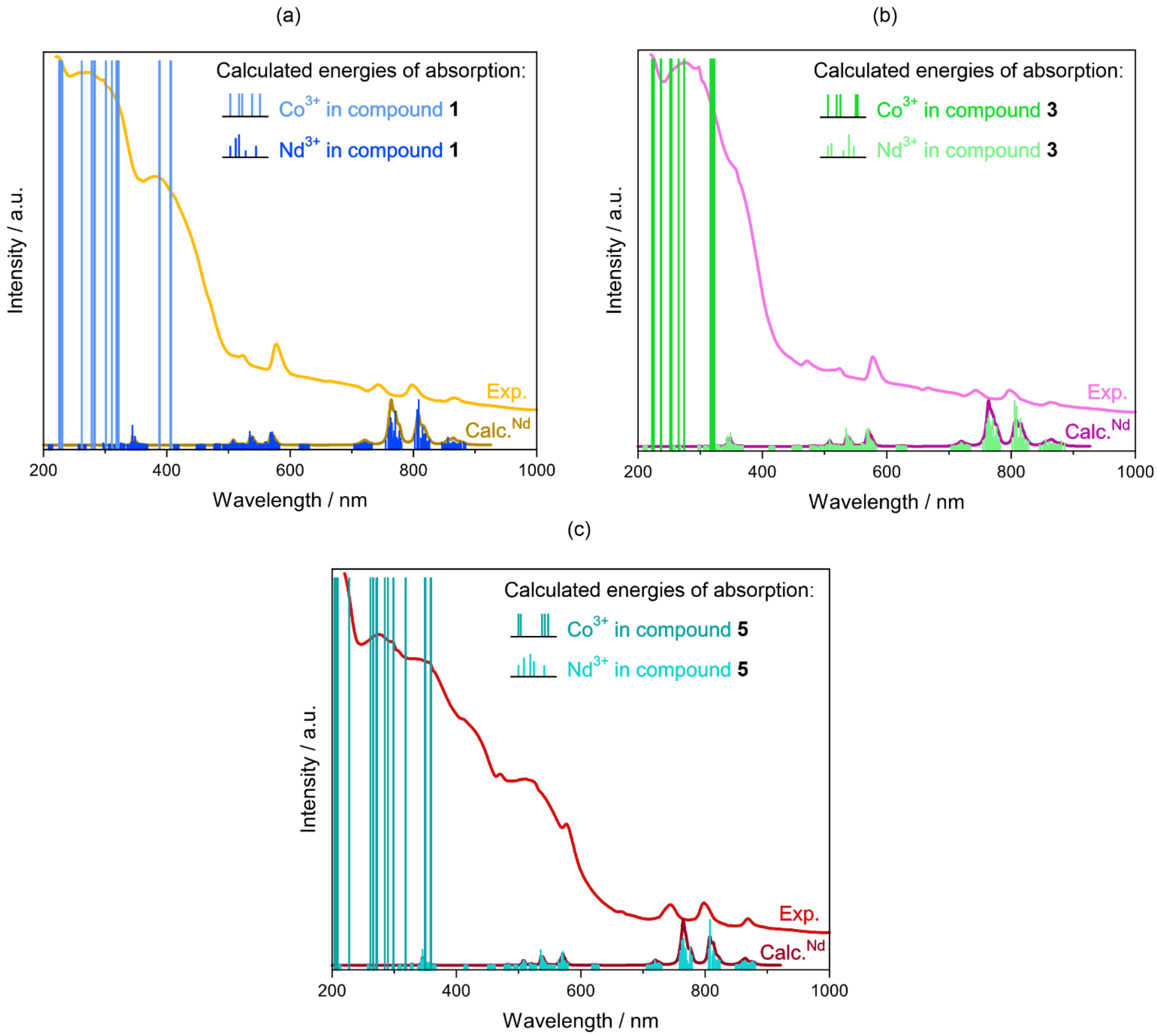

Figure S37. *Ab initio* (CASSCF) calculated wavelengths (colored vertical lines) of light absorption bands for Co^{3+} and Nd^{3+} ions embedded in compounds **1** (a), **3** (b), and **5** (c), compared with the experimental solid-state UV-vis-NIR spectra of these compounds (Exp.). The calculated spectra (Calc.Nd) correspond to the absorption bands related to f-f electronic transitions of Nd(III) centers. The relative intensities for the related bands were calculated and visualized by the heights of colored bars. All the spectra were normalized. Calculations were performed for the temperature of 300 K to match experimental conditions.

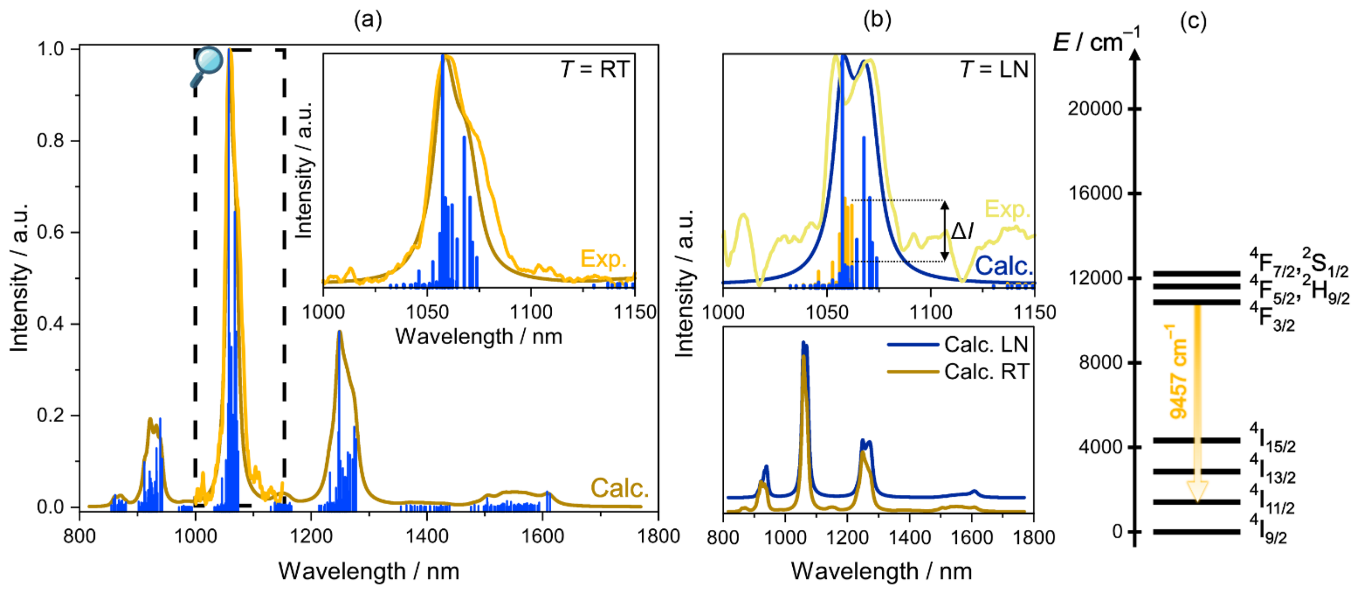

Figure S38. The *ab initio* (CASSCF) calculated emission spectrum of compound **1** (Calc., with calculated wavelengths, representing transition energies, and intensities shown as vertical bars) at room temperature (RT) with the magnification of the area where the experimental spectrum (Exp.) was recorded for comparison (a), the comparison of the calculated emission spectra at RT and liquid nitrogen temperature (LN) (b, bottom), shown with the colored bars representing the transition energies and relative intensities of the bands and compared with the experiment at LN (b, top), and the fragment of the energy level diagram for Nd(III) centers in **1** with the indicated transition energy found in experiment (c). In part (b, top), the largest difference in the calculated intensity between RT and LN was indicated as *ΔI*. All the spectra were normalized.

Comment to Figure S38 and the two next Figures (S39 and S40). The reader should note that it was only possible to experimentally observe $^4F_{3/2} \rightarrow {}^2I_{11/2}$ transitions, while simulations grant us access to all of them. Because of this, the recorded spectra do not give us further insight into the $^4I_{9/2}$ ground multiplet studied for magnetism. To match the simulated photoluminescence spectra perfectly with the experimental one, we employed the scaling of 0.75 (different than for the absorption spectra, see the main text for details) to account for the observed Stokes shift, and we used for the convolution Lornetzians of FWHM = 100 cm^{-1}.

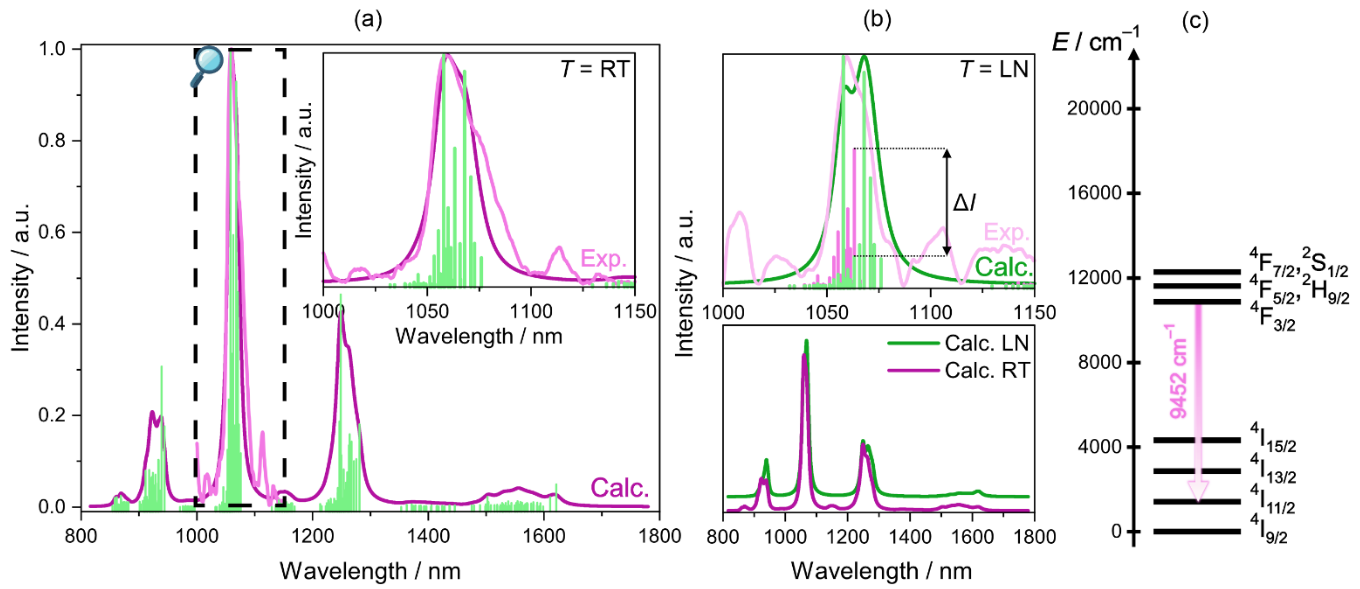

Figure S39. The *ab initio* (CASSCF) calculated emission spectrum of compound **3** (Calc., with calculated wavelengths, representing transition energies, and intensities shown as vertical bars) at room temperature (RT) with the magnification of the area where the experimental spectrum (Exp.) was recorded for comparison (a), the comparison of the calculated emission spectra at RT and liquid nitrogen temperature (LN) (b, bottom), shown with the colored bars representing the transition energies and relative intensities of the bands and compared with the experiment at LN (b, top), and the fragment of the energy level diagram for Nd(III) centers in **3** with the indicated transition energy found in experiment (c). In part (b, top), the largest difference in the calculated intensity between RT and LN was indicated as *ΔI*. All the spectra were normalized.

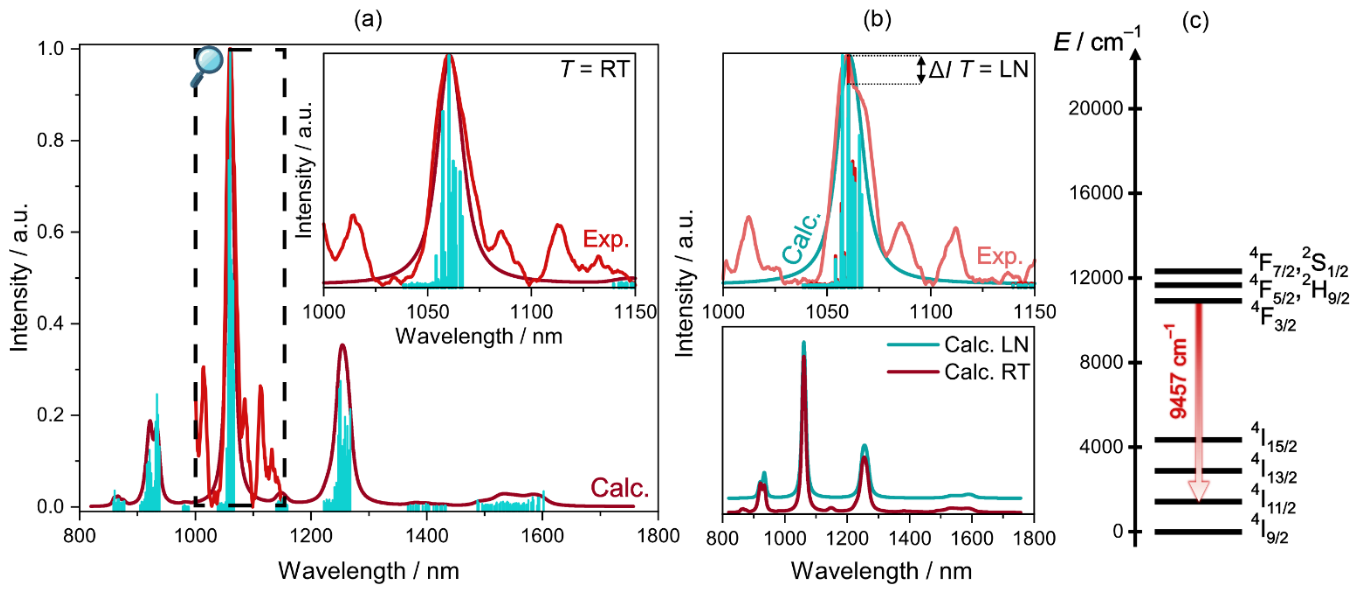

Figure S40. The *ab initio* (CASSCF) calculated emission spectrum of compound **5** (Calc., with calculated wavelengths, representing transition energies, and intensities shown as vertical bars) at room temperature (RT) with the magnification of the area where the experimental spectrum (Exp.) was recorded for comparison (a), the comparison of the calculated emission spectra at RT and liquid nitrogen temperature (LN) (b, bottom), shown with the colored bars representing the transition energies and relative intensities of the bands and compared with the experiment at LN (b, top), and the fragment of the energy level diagram for Nd(III) centers in **5** with the indicated transition energy found in experiment (c). In part (b, top), the largest difference in the calculated intensity between RT and LN was indicated as *ΔI*. All the spectra were normalized.

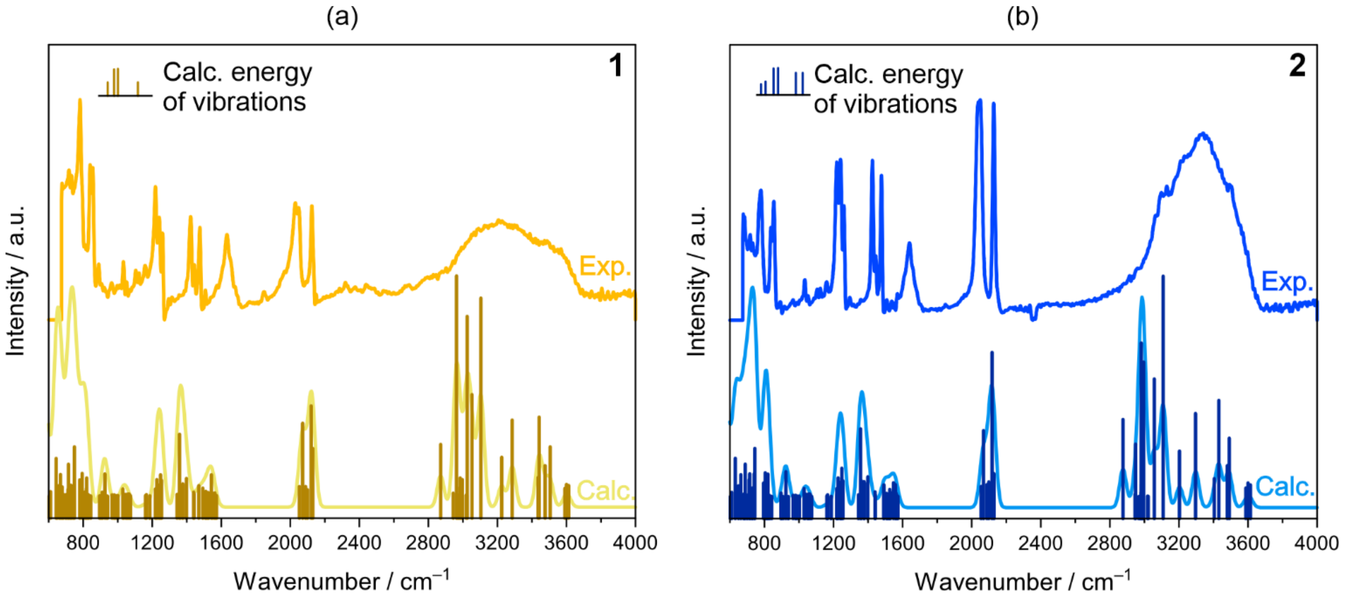

Figure S41. DFT-calculated (Calc.) and experimental (Exp.) infrared (IR) spectra of compounds **1** (a) and **2** (b). Computational spectra were obtained by convolution with Lorentzian functions of FWHM = 10 cm^{-1}, using the calculated energies and relative intensities (visualized also as vertical bars). The energies of the calculated vibrational modes were scaled by a factor of 0.92. They are gathered in Table S23. Information about experimental IR spectra can be found in Figure S2.

Table S23. (part 1) DFT-calculated energies of vibrational modes for compounds **1** and **2**.

Vibration number	Energy of vibration / cm⁻¹		Vibration number	Energy of vibration / cm⁻¹		Vibration number	Energy of vibration / cm⁻¹	
	Compound 1	Compound 2		Compound 1	Compound 2		Compound 1	Compound 2
6	10.85	12.13	54	237.84	233.25	102	532.62	533.11
7	18.97	19.50	55	240.18	245.34	103	538.04	535.01
8	23.04	21.26	56	250.99	247.24	104	552.63	553.56
9	26.42	27.67	57	251.74	249.89	105	555.22	555.03
10	31.31	31.02	58	258.21	256.97	106	555.30	555.62
11	36.52	36.77	59	261.21	259.76	107	558.40	557.60
12	38.43	38.71	60	268.08	265.40	108	559.35	558.53
13	44.08	44.26	61	270.36	267.22	109	560.07	563.22
14	52.58	50.53	62	277.57	276.25	110	581.17	576.65
15	57.93	55.99	63	279.60	277.87	111	603.23	603.18
16	60.64	60.24	64	286.56	282.59	112	603.58	603.43
17	61.85	61.68	65	294.93	290.00	113	610.09	610.08
18	72.51	68.99	66	298.31	302.97	114	643.94	630.64
19	74.48	73.10	67	305.37	307.88	115	649.79	649.93
20	76.89	73.46	68	311.45	310.54	116	661.26	663.95
21	80.35	78.31	69	316.34	313.73	117	667.62	679.13
22	83.16	81.64	70	318.86	317.80	118	678.09	681.81
23	84.61	82.99	71	320.34	321.14	119	679.45	685.67
24	91.59	87.58	72	329.96	329.50	120	685.60	688.92
25	92.57	90.52	73	335.17	331.94	121	698.47	698.58
26	94.54	92.29	74	340.18	335.11	122	700.60	700.65
27	100.44	99.16	75	348.73	349.59	123	711.53	711.96
28	104.27	103.29	76	359.03	359.89	124	715.13	714.62
29	107.04	105.10	77	365.18	365.97	125	719.08	718.78
30	113.46	112.46	78	370.83	380.42	126	728.30	728.45
31	116.24	113.87	79	380.11	382.55	127	728.58	729.60
32	117.20	115.97	80	394.54	399.33	128	730.36	730.08
33	122.07	118.67	81	403.60	403.52	129	741.28	740.46
34	125.87	122.73	82	408.21	407.93	130	748.64	744.44
35	128.00	127.36	83	418.92	419.25	131	779.51	794.73
36	134.08	134.30	84	424.50	425.09	132	793.68	804.43
37	142.89	144.23	85	430.38	430.73	133	810.90	807.11
38	149.71	146.47	86	449.82	455.30	134	812.31	811.15
39	151.40	147.83	87	454.96	459.98	135	817.88	813.71
40	157.54	155.04	88	464.88	464.49	136	818.50	818.59
41	162.57	159.06	89	467.84	469.03	137	819.83	819.56
42	165.80	165.04	90	469.74	469.70	138	821.37	821.28
43	171.89	169.57	91	480.57	481.96	139	829.33	828.66
44	179.33	178.12	92	484.14	484.27	140	831.43	832.88
45	182.30	179.37	93	496.60	495.83	141	843.22	842.24
46	184.95	184.42	94	502.73	502.36	142	895.00	895.55
47	189.26	186.82	95	504.14	503.63	143	900.86	896.19
48	194.12	191.40	96	505.62	505.14	144	902.23	902.08
49	197.68	195.03	97	509.29	509.12	145	910.22	902.54
50	208.88	204.05	98	510.09	509.71	146	911.18	910.49
51	220.30	214.82	99	513.19	513.65	147	926.27	924.79
52	229.40	224.32	100	515.10	514.55	148	936.74	936.89
53	235.61	230.20	101	530.88	530.41	149	938.46	939.55

Table S23. (part 2) DFT-calculated energies of vibrational modes for compounds **1** and **2**.

Vibration number	Energy of vibration / cm^{-1}		Vibration number	Energy of vibration / cm^{-1}		Vibration number	Energy of vibration / cm^{-1}	
	Compound 1	Compound 2		Compound 1	Compound 2		Compound 1	Compound 2
150	940.68	940.22	180	1344.10	1343.98	210	2870.96	2876.02
151	967.63	965.77	181	1358.12	1357.24	211	2944.67	2946.24
152	980.23	980.08	182	1361.78	1361.42	212	2962.90	2949.38
153	980.43	980.53	183	1376.61	1376.48	213	2965.27	2965.13
154	984.60	984.76	184	1377.49	1376.64	214	2971.82	2971.70
155	991.48	990.44	185	1396.94	1396.87	215	2976.95	2977.15
156	999.99	1000.22	186	1398.38	1398.21	216	2977.30	2977.29
157	1001.48	1001.34	187	1441.00	1441.06	217	2981.70	2980.63
158	1028.89	1029.32	188	1442.20	1441.86	218	2983.62	2981.66
159	1036.09	1035.68	189	1470.11	1491.88	219	2985.20	2983.43
160	1048.83	1048.89	190	1493.07	1492.19	220	2986.19	2985.91
161	1052.64	1052.08	191	1508.96	1507.48	221	2987.16	2987.09
162	1059.57	1059.48	192	1509.63	1508.53	222	2992.57	2987.62
163	1066.63	1065.60	193	1513.23	1512.29	223	2992.65	2991.85
164	1072.78	1072.90	194	1518.00	1517.94	224	2993.80	2992.62
165	1073.75	1073.64	195	1521.81	1521.83	225	2996.51	2992.63
166	1160.94	1160.87	196	1522.43	1525.37	226	3019.27	2996.04
167	1166.01	1165.15	197	1527.04	1528.10	227	3023.40	3018.15
168	1185.65	1185.68	198	1543.36	1547.29	228	3024.09	3022.16
169	1186.38	1186.03	199	1549.57	1557.84	229	3052.23	3057.26
170	1218.49	1218.85	200	1561.54	1561.55	230	3104.01	3108.79
171	1219.84	1219.19	201	1562.39	1562.22	231	3224.55	3203.84
172	1226.26	1225.95	202	1570.85	1570.79	232	3286.04	3296.63
173	1227.12	1226.83	203	1571.35	1571.55	233	3436.24	3404.46
174	1240.34	1238.26	204	2052.33	2054.22	234	3441.48	3431.26
175	1241.38	1240.79	205	2070.70	2067.82	235	3475.61	3481.15
176	1243.39	1242.44	206	2087.92	2088.25	236	3505.26	3491.55
177	1243.52	1243.10	207	2099.04	2098.93	237	3591.45	3588.26
178	1247.93	1248.05	208	2120.27	2117.37	238	3600.05	3600.11
179	1254.48	1253.78	209	2130.04	2128.22	239	3612.74	3614.34

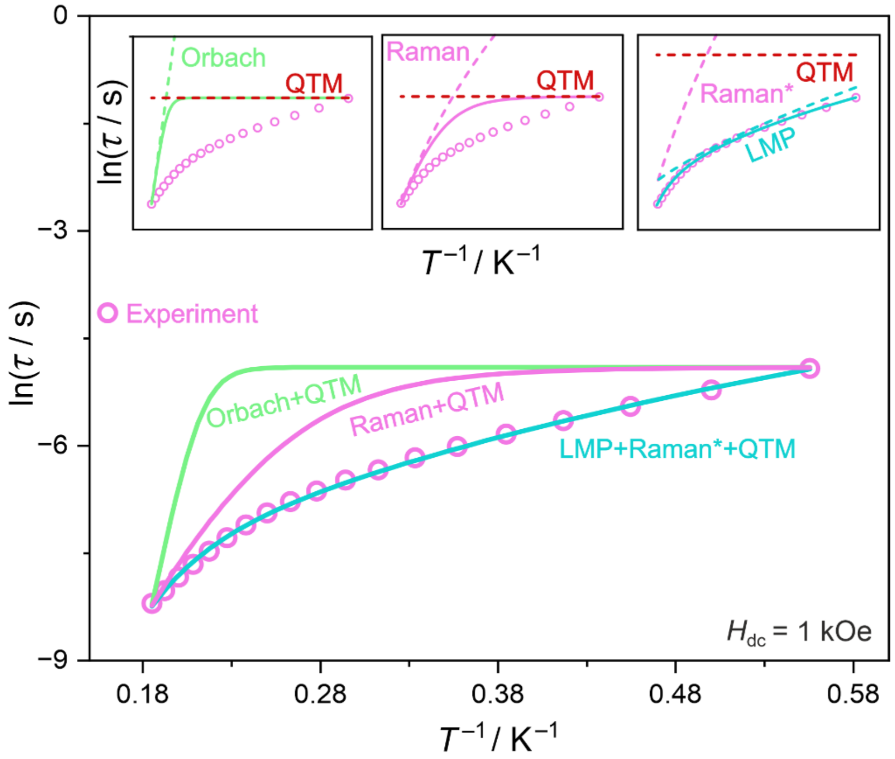

Figure S42. Comparison of curves representing different potential combinations of typical magnetic relaxation processes (Orbach, QTM, Raman, Local Mode Process = LMP) with experimental relaxation times for **3**. Smaller graphs represent a few considered model separately, additionally showing their components. For comparison, see the related Figures S18 and S19, and the main text.

Comment for Figure S42, on using the LMP process in the fitting of *ac* magnetic data in reported compounds. It is worth explaining why we decided to use the LMP term, associated with the simultaneous absorption and emission of a pair of low-lying degenerate phonons for doublet in zero *dc* field where Kramer's degeneracy is not broken. We can justify it by the previously mentioned minimal Zeeman splitting, which in the direction of the main magnetic axis in H_{dc} = 2 kOe does not exceed 0.5 cm^{-1} even for **3**. That value is well within the phonon line width and their dispersion. Moreover, it could eventually turn out that LMP is indeed weakly field-dependent because of the splitting introduced by the magnetic field, and what is manifesting in our model (which neglects any field dependence of LMP) as a direct process for **1** and field-dependent Raman for **3** is indeed driven by the *D* parameter LMP modulation. That cannot be answered without thorough dynamical simulations.

Description of Supporting Movies

Movie 1. Visualization of the vibrational mode showing the wavenumber (10.85 cm^{-1}) that is closest to the determined best-fit value of the wavenumber of the local mode process (LMP) of slow magnetic relaxation in compound **1** (compare the best-fit parameters from Table 1 with the calculated energies of vibrations provided in Table S23).

Movie 2. Visualization of the vibrational mode showing the wavenumber (12.13 cm^{-1}) that is closest to the determined best-fit value of the wavenumber of the local mode process (LMP) of slow magnetic relaxation in compound **2** (compare the best-fit parameters from Table 1 with the calculated energies of vibrations provided in Table S23).

Movie 3. Evolution of the magnetic-field dependence (at the indicated temperature) of the directional susceptibility in the form of its product with temperature for compound **1** (see Figure 4 for comparison).

Movie 4. Evolution of the temperature dependence (at the indicated magnetic field) of the directional susceptibility in the form of its product with temperature for compound **1** (see Figure 4 for comparison).

Movie 5. Evolution of the magnetic-field dependence (at the indicated temperature) of the directional susceptibility in the form of its product with temperature for compound **5** (see Figure 4 for comparison).

Movie 6. Evolution of the temperature dependence (at the indicated magnetic field) of the directional susceptibility in the form of its product with temperature for compound **5** (see Figure 4 for comparison).

Additional discussion on scaling of the computational results to the experimental data

As already mentioned in the main manuscript, the scaling factors were employed for the results of theoretical calculations (both DFT as well as the part of *ab initio*) to match the experimental data. In the case of DFT, we employed the IR spectra to scale the computational results, which resulted in a scaling factor of 0.92. In general, it is well-known that because of anharmonic effects, incomplete incorporation of electron correlation, and the use of finite basis sets, DFT methods pretty much always give frequencies larger than experimentally observed; therefore, they have to always be calibrated using some scaling factors based on, e.g., IR spectra. Such treatment is possible because of the relatively uniform error of DFT methods for vibrational modes. Moreover, the broadly cited specialized paper on this issue indicates that for the PBE0 functional,[S14] used during our investigation, a scaling factor of around 0.95, depending on a basis set, should be used. We found a factor of 0.92 for the best recreation of the experiment by our DFT approach; however, it is also dependent on Lorentzian functions FWHM used to convolute the spectra. It is worth mentioning that such calibrations are widely used in the investigation of Single-Molecule Magnets by the combined DFT and *ab initio* methodology,[S15] where the similar PBE functional was used, and the best calibration of the results was found for a scaling factor of 0.9485. We could have also used a factor closer to 0.95 or exactly this number, but it would not alter our conclusions since the lowest frequencies would change only a little bit, e.g., for **1** from 10.85 to 11.20 cm^{-1}, which is still relatively close to the obtained experimental best-fit parameter of $\hbar\omega$ = 8.9 ± 0.9 cm^{-1}. Thus, we decided to leave the primary scaling factor of 0.92, which was obtained with the help of IR spectra as discussed above.

Then, we also use scaling factors for the multiconfigurational CASSCF method used for the simulation of optical properties to match experimental absorption UV-vis as well as luminescence spectra. Here, the origins of overestimation are multiple. First, the calculations were conducted using relatively small basis sets with minimal active space (only 4f orbitals) and for experimental geometry from XRD experiments. Therefore, they were not performed for the ground energy minimum of the method. Second, there is a complete lack of inclusion of dynamical electron correlation for Nd(III) compounds because we were not able to use CASPT2 or NEVPT2 treatments (for their importance, see, e.g., ref. S16) due to the number of electron configurations and size of clusters. Next, simply taking energy differences between SA-CASSCF states for transitions is an approximation itself as it omits possible orbital relaxation for the excited states. To overcome the last mentioned problem, one should turn to methods within the linear response theory formalism such as MC-RPA[S17] for CASSCF which can be seen as the exact analog of TD-DFT for electronic transitions of systems with DFT-optimised ground electron density. Unfortunately, to our knowledge, there are no quantum chemical packages offering the inclusion of Spin-Orbit effects within this formalism yet. Finally, State-Average CASSCF calculation, in which the orbitals are optimized with respect to a weighted mean of all states of interest (Mennucci, B., Computational Spectroscopy: Methods, Experiments, Applications, **2010**, *5*, 151–171.) instead of optimization for each state separately, introduces intrinsic error closely related to states and orbitals relaxation for each transition. Moreover, simulations are conducted for gas phase clusters while the experiment is recorded for the solid state. In the manuscript, we compared results for the absorption transitions of $[Co^{III}(CN)_5(X)]^{3-}$ precursors, which, because of small sizes, were treated with CASSCF/NEVPT2 level of theory and Nd^{III}-based compounds treated without dynamical electron correlation effects and scaling needed for matching experimental spectra is indeed smaller for previous (scaling factor of 0.93) than for later (scaling factor of 0.82). Currently, even the most advanced approaches with the inclusion of phonon effects for precise predicting of phosphorescence rates for small organic molecules use rigid shifts to match transition energies to the experiment (see, e.g., ref. S18). In this regard, used scaling factors are reasonable and represent the character of the employed theoretical methodologies and their intrinsic limitations.

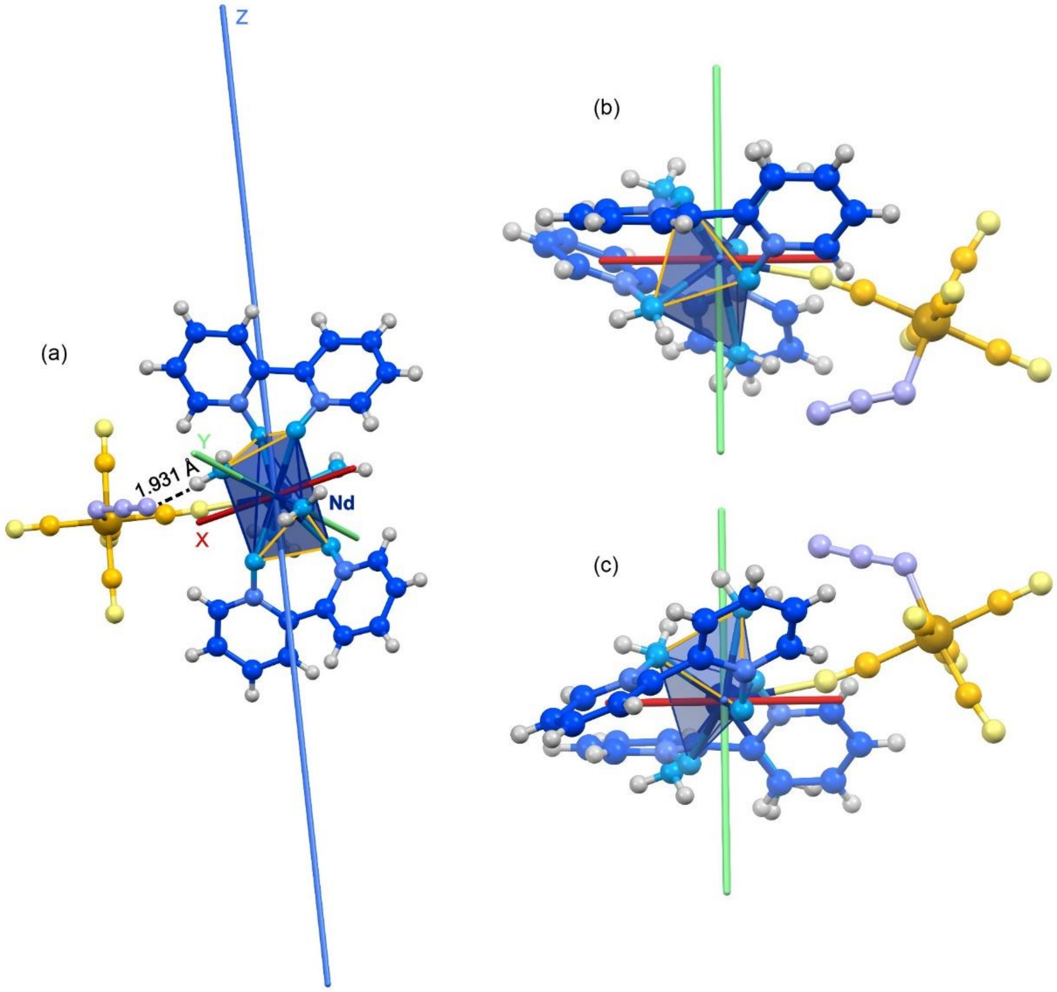

Figure S43. Visualization of the arrangement of the *ab-initio*-calculated main magnetic axes (X, Y, Z) within the dinuclear molecule of compound **1**, presented in three different views (a, b, c) which were selected to illustrate the position of the Z-axis in the relation to the trigonal prismatic part (blue polyhedron with yellow edges for the triangular walls) of the coordination polyhedron of Nd(III) centers that can be described as a strongly distorted tricapped trigonal prism (Table S11). For comparison, please also check Figure S28 where the same magnetic axes were presented differently, to compare their alignment with the relative position of two embedded 2,2'-bpdo ligands. The distance between the azido ligand of Co(III) complexes and the hydrogen atom of the coordinated water molecule was marked as this interaction seems to be crucial for the structural distortion of the trigonal prismatic fragment of the Nd(III) polyhedron (see the text of the manuscript for details).

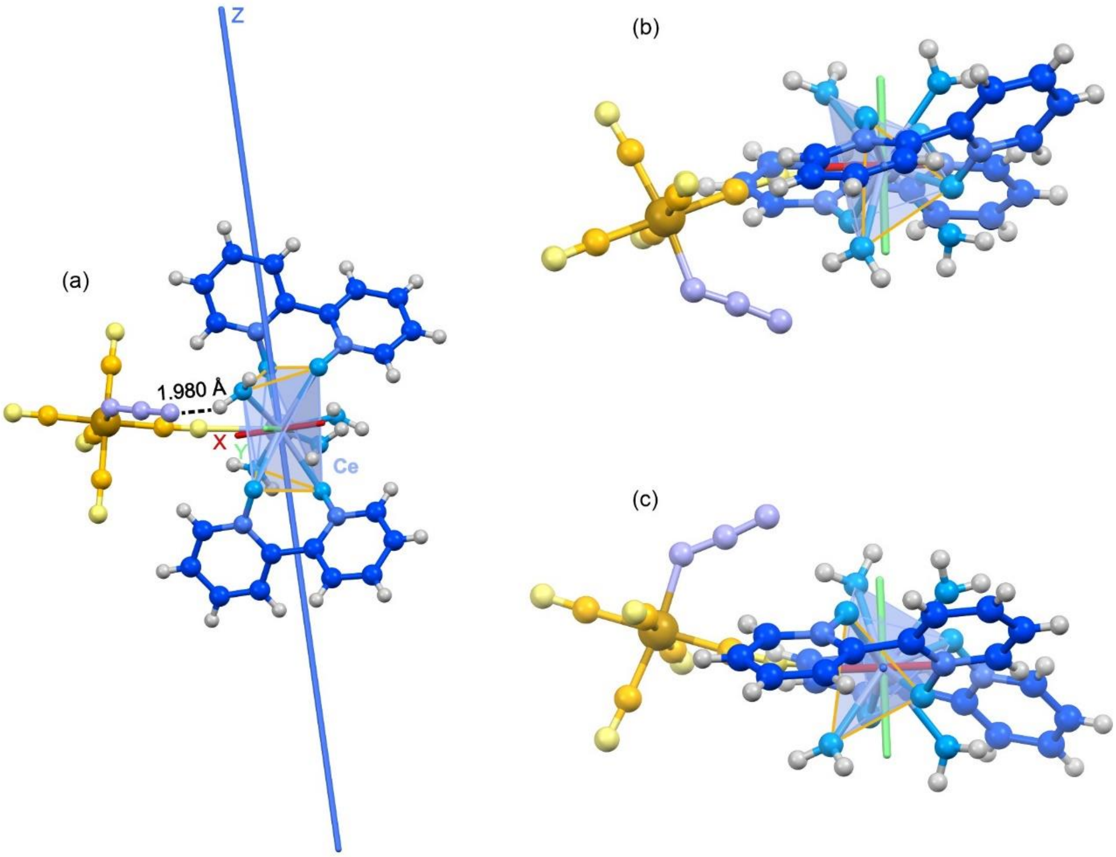

Figure S44. Visualization of the arrangement of the *ab-initio*-calculated main magnetic axes (X, Y, Z) within the dinuclear molecule of compound **2**, presented in three different views (a, b, c) which were selected to illustrate the position of the Z-axis in the relation to the trigonal prismatic part (blue polyhedron with yellow edges for the triangular walls) of the coordination polyhedron of Ce(III) centers that can be described as a strongly distorted tricapped trigonal prism (Table S11). For comparison, please also check Figure S29 where the same magnetic axes were presented differently, to compare their alignment with the relative position of two embedded 2,2'-bpdo ligands. The distance between the azido ligand of Co(III) complexes and the hydrogen atom of the coordinated water molecule was marked as this interaction seems to be crucial for the structural distortion of the trigonal prismatic fragment of the Ce(III) polyhedron (see the text of the manuscript for details).

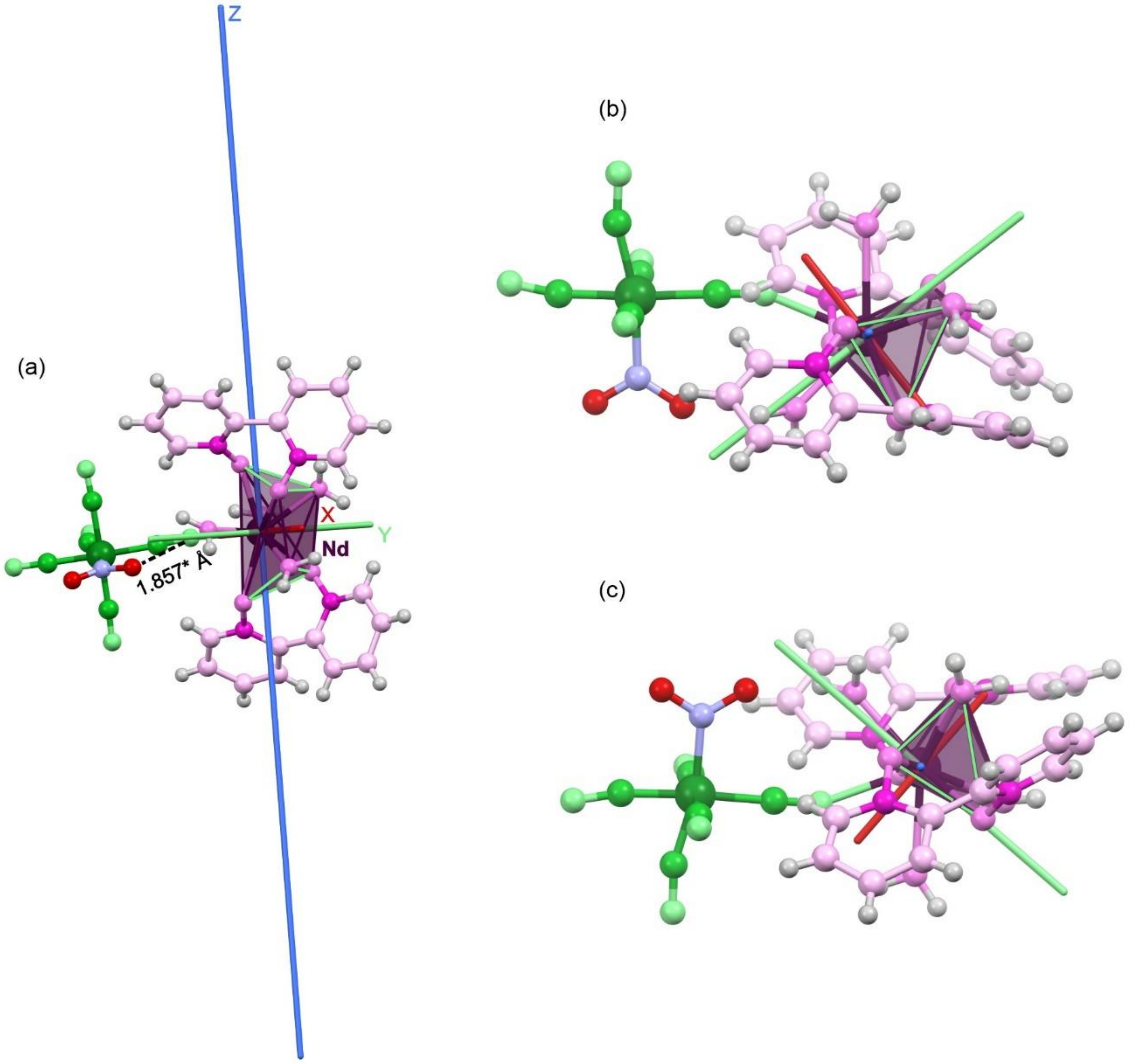

Figure S45. Visualization of the arrangement of the *ab-initio*-calculated main magnetic axes (X, Y, Z) within the dinuclear molecule of compound **3**, presented in three different views (a, b, c) which were selected to illustrate the position of the Z-axis in the relation to the trigonal prismatic part (blue polyhedron with yellow edges for the triangular walls) of the coordination polyhedron of Nd(III) centers that can be described as a strongly distorted tricapped trigonal prism (Table S11). For comparison, please also check Figure S28 where the same magnetic axes were presented differently, to compare their alignment with the relative position of two embedded 2,2'-bpdo ligands. The distance between the nitrito ligand of Co(III) complexes and the coordinated water molecule (the asterisk was given as the average position of two H-atoms was provided) was marked as this interaction seems to be crucial for the structural distortion of the trigonal prismatic fragment of the Nd(III) polyhedron (see the text of the manuscript for details).

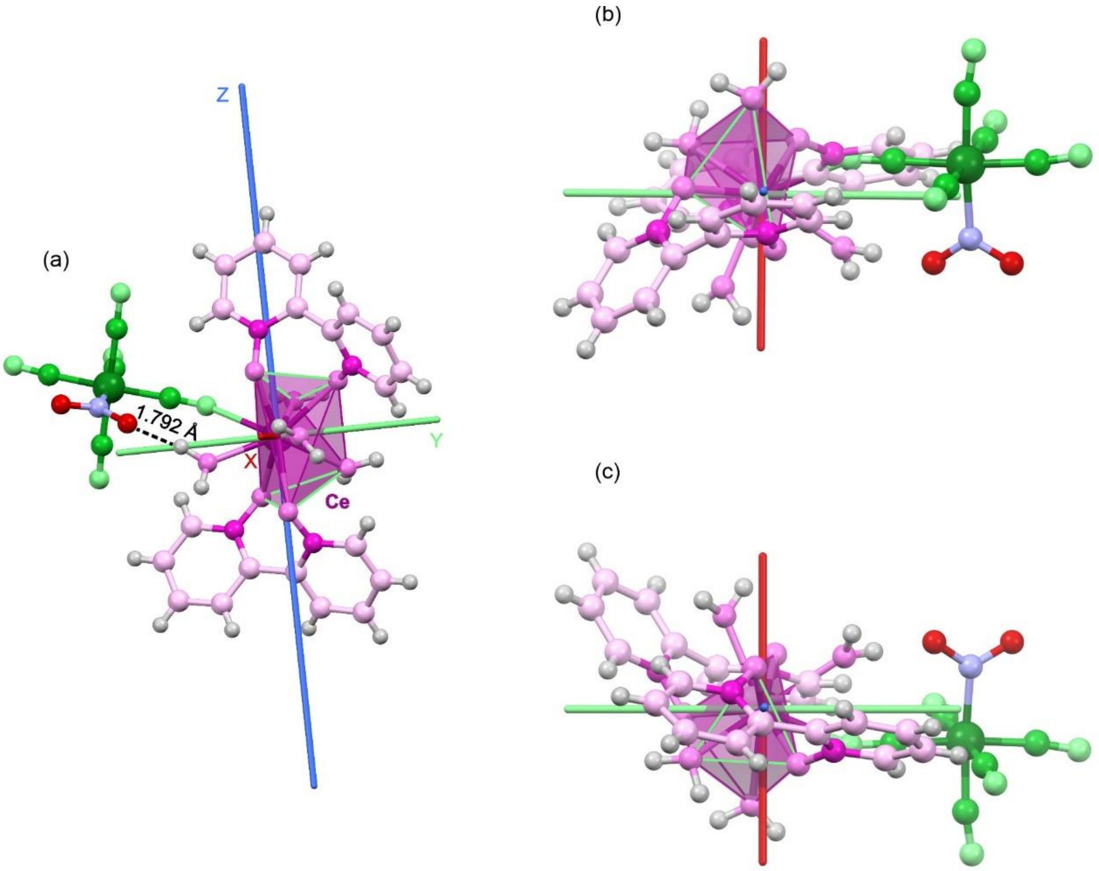

Figure S46. Visualization of the arrangement of the *ab-initio*-calculated main magnetic axes (X, Y, Z) within the dinuclear molecule of compound **4**, presented in three different views (a, b, c) which were selected to illustrate the position of the Z-axis in the relation to the trigonal prismatic part (blue polyhedron with yellow edges for the triangular walls) of the coordination polyhedron of Ce(III) centers that can be described as a strongly distorted tricapped trigonal prism (Table S11). For comparison, please also check Figure S29 where the same magnetic axes were presented differently, to compare their alignment with the relative position of two embedded 2,2'-bpdo ligands. The distance between the nitrito ligand of Co(III) complexes and the hydrogen atom of the coordinated water molecule was marked as this interaction seems to be crucial for the structural distortion of the trigonal prismatic fragment of the Ce(III) polyhedron (see the text of the manuscript for details).

References to the Supporting Information

(S1) Linhard, M.; Flygare, H. Über Komplexverbindungen. IV. Azido-Pentamminkobalt(III)-Komplexe *Z. Anorg. Allg. Chem.* **1950**, *262*, 328–343.

(S2) Barca, R.; Ellis, J.; Tsao, M.-S.; Willmarth, W. K. The Pentacyano Complexes of Cobalt(III). IV. Kinetics and Mechanism of the Substitution of Water in $Co(CN)_5OH_2^{2-}$ by Pyridine, Ammonia, Hydrazine, and Hydrazinium Ion. *Inorg. Chem*. **1967**, *6*, 243–248.

(S3) Flor, T.; Casabó, J. New Synthesis of Acidopentacyanocobaltate(III) Complexes. *Synth. React. Inorg. Met.-Org. Chem*. **1986**, *16*, 795–800.

(S4) Zakrzewski, J. J.; Kumal, K.; Zychowicz, M.; Jankowski, R.; Wyczesany, M.; Sieklucka, B.; Ohkoshi, S.; Chorazy S. Combined Experimental and Ab Initio Methods for Rationalization of Magneto-Luminescent Properties of Yb^{III} Nanomagnets Embedded in Cyanido/Thiocyanidometallate-Based Crystals. *J. Phys. Chem. Lett.* **2021**, *12*, 10558–10566

(S5) Sheldrick, G. M. *SHELXT* - Integrated Space-Group and Crystal-Structure Determination. *Acta Cryst.* **2015**, A71, 3–8.

(S6) Farrugia, L. J. WinGX and ORTEP for Windows an Update. *J. Appl. Crystallogr.* **2012**, *45*, 849–854.

(S7) Llunell, M.; Casanova, D.; Cirera, J.; Bofill, J.; Alemany, P.; Alvarez, S.; Pinsky, M.; Avnir, D. SHAPE v. 2.1. Program for the Calculation of Continuous Shape Measures of Polygonal and Polyhedral Molecular Fragments, University of Barcelona Barcelona, Spain, 2013.

(S8) Casanova, D.; Cirera, J.; Llunell, M.; Alemany, P.; Avnir, D.; Alvarez, S. Minimal Distortion Pathways in Polyhedral Rearrangements. *J. Am. Chem. Soc.* **2004**, *126*, 1755–1763.

(S9) Ruinz-Martines, A.; Casanova, D.; Alvarez S. Polyhedral Structures with an Odd Number of Vertices Nine-Coordinate Metal Compounds. *Chem. Eur. J.* **2008**, *14*, 1291–1303.

(S10) Sinha, S. P. Spectroscopic Investigations of Some Neodymium Complexes. Spectroscopic Investigations of Some Neodymium Complexes. *Spectrochem. Acta* **1966**, *22*, 57–62.

(S11) Mohan, S.; Thind, K. S.; Sharma, G.; Gerward, L. Spectroscopic Investigations of Nd^{3+} Doped Fluoro- and Chloro-Borate Glasses. *Spectrochem. Acta A* **2008**, *70*, 1173–1179.

(S12) Liberka, M.; Zychowicz, M.; Zychowicz, W.; Chorazy, S. Neutral Dicyanidoferrate(II) Metalloligands for the Rational Design of Dysprosium(III) Single-Molecule Magnets. *Chem. Commun*. **2022**, *58*, 6381–6384.

(S13) Hunter, J. D. Matplotlib: A 2D Graphics Environment. *Comput. Sci. Eng.* **2007**, *9*, 90–95.

(S14) Merrick, J. P.; Moran, D.; Radom, L. An Evaluation of Harmonic Vibrational Frequency Scale Factors. *J. Phys. Chem. A* **2007**, *111*, 11683–11700.

(S15) Goodwin, C. A. P.; Ortu, F.; Reta, D.; Chilton, N. F.; Mills, D. P. Molecular magnetic hysteresis at 60 kelvin in dysprosocenium. *Nature* **2017**, *548*, 439–442.

(S16) Babetto, L.; Carlotto, S.; Carlotto, A.; Rancan, M.; Bottaro, G.; Armelao, L.; Casarin, M. Multireference *Ab Initio* Investigation on Ground and Low-Lying Excited States: Systematic Evaluation of *J–J* Mixing in a Eu^{3+} Luminescent Complex. *Inorg. Chem.* **2021**, *60*, 315–324.

(S17) Jörgensen, P.; Jensen, H. J. A.; Olsen, J. Linear response calculations for large scale multiconfiguration self-consistent field wave functions. *J. Chem. Phys.* **1988**, *89*, 3654–3661.

(S18) de Souza, B.; Farias, G.; Neese, F.; Izsák, R. Predicting Phosphorescence Rates of Light Organic Molecules Using Time-Dependent Density Functional Theory and the Path Integral Approach to Dynamics. *J. Chem. Theory Comput.* **2019**, *15*, 1896–1904.
