# Supplementary figures and images for: Synergy of Experiment and Broadened Exploration of Ab Initio Calculations for Understanding of Lanthanide–Pentacyanidocobaltate Molecular Nanomagnets and Their Optical Properties
Source: Inorg Chem. 2024 Sep 2;63(41):19213–26. doi: 10.1021/acs.inorgchem.4c02793 (PMC11483780; doi:10.1021/acs.inorgchem.4c02793)

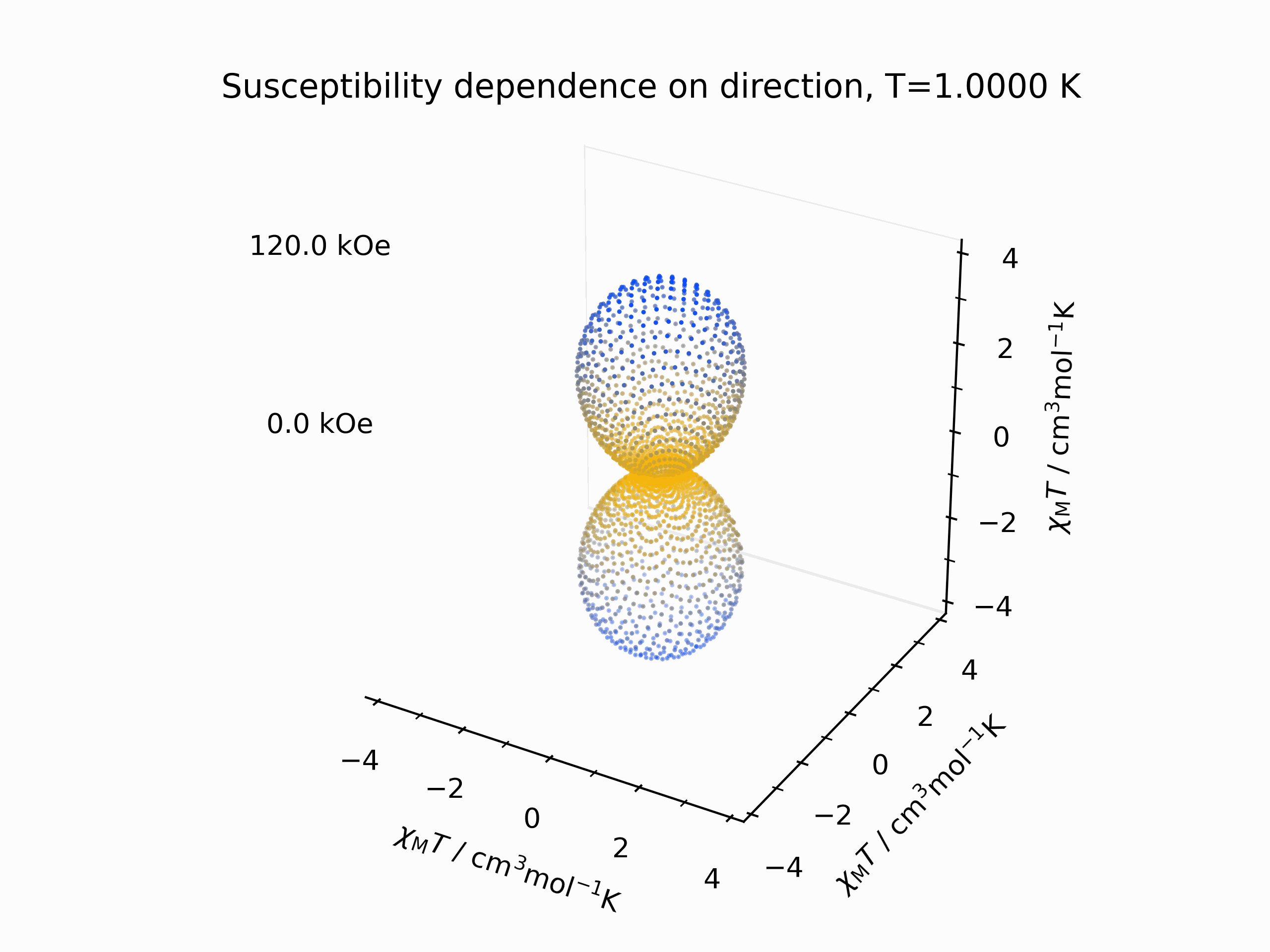

Supplement: Supplementary file 4 — ic4c02793_si_004.zip [file ic4c02793_si_004.zip › Supporting Movie 3.gif]

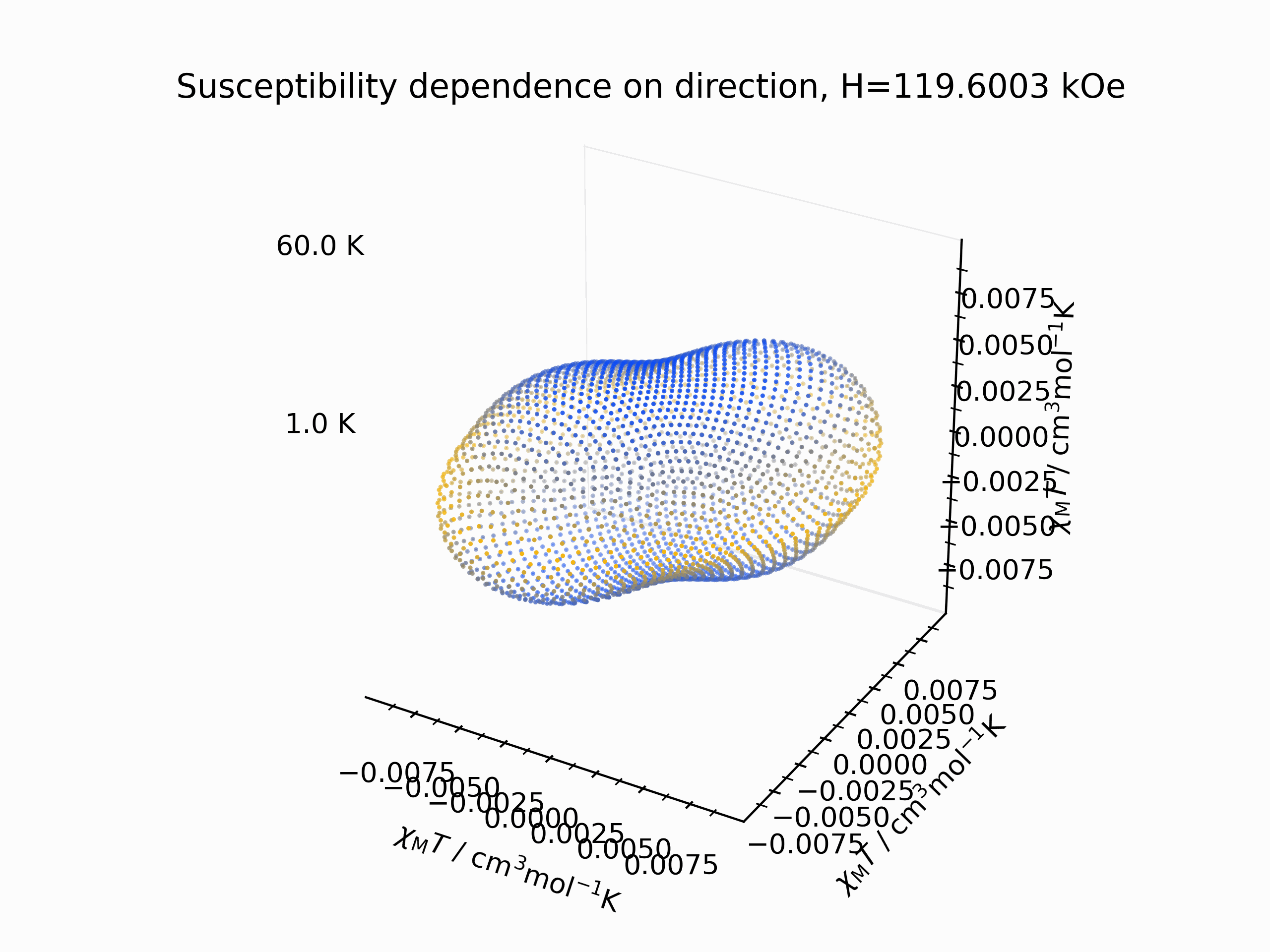

Supplement: Supplementary file 4 — ic4c02793_si_004.zip [file ic4c02793_si_004.zip › Supporting Movie 4.gif]

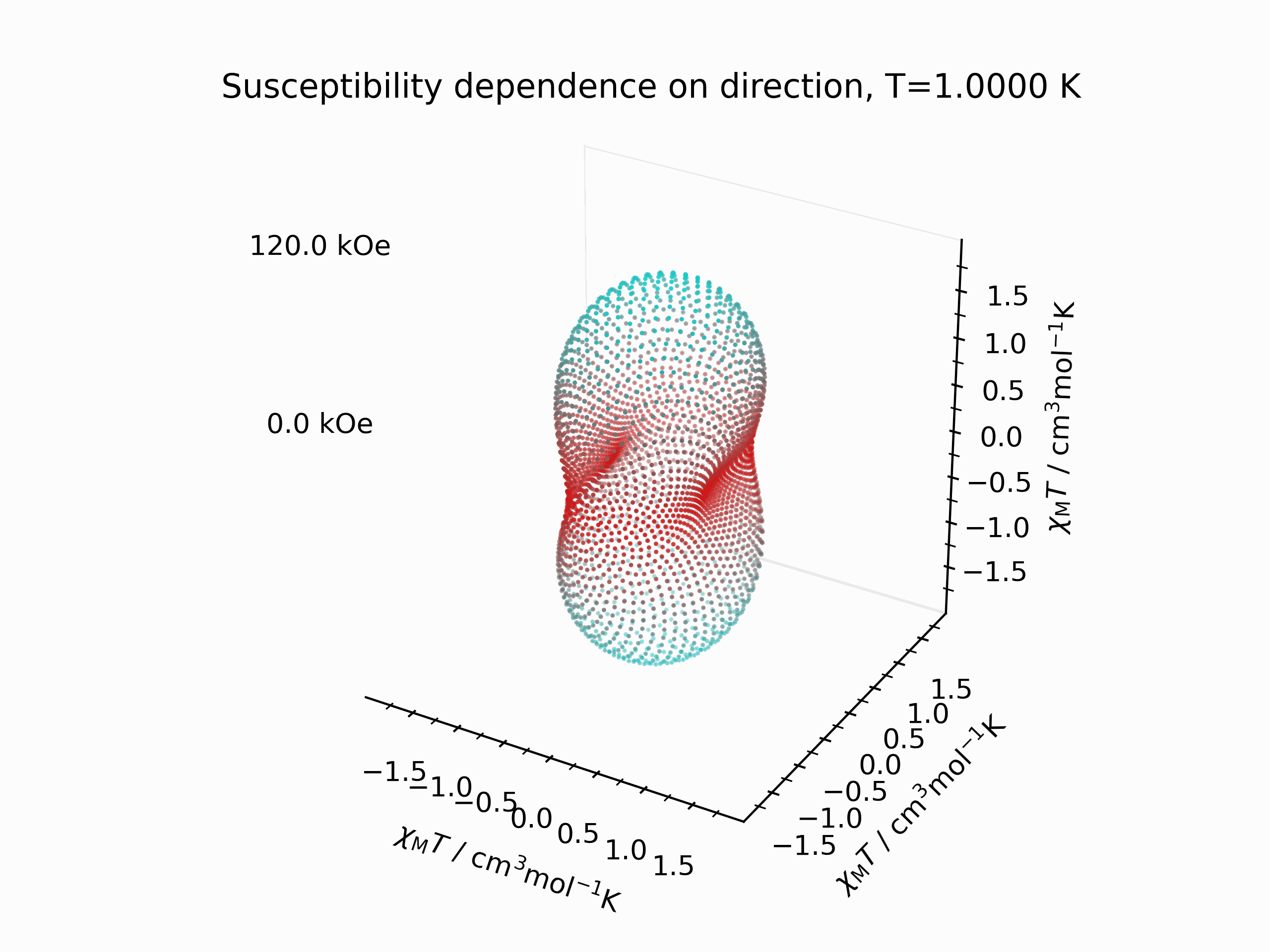

Supplement: Supplementary file 4 — ic4c02793_si_004.zip [file ic4c02793_si_004.zip › Supporting Movie 5.gif]

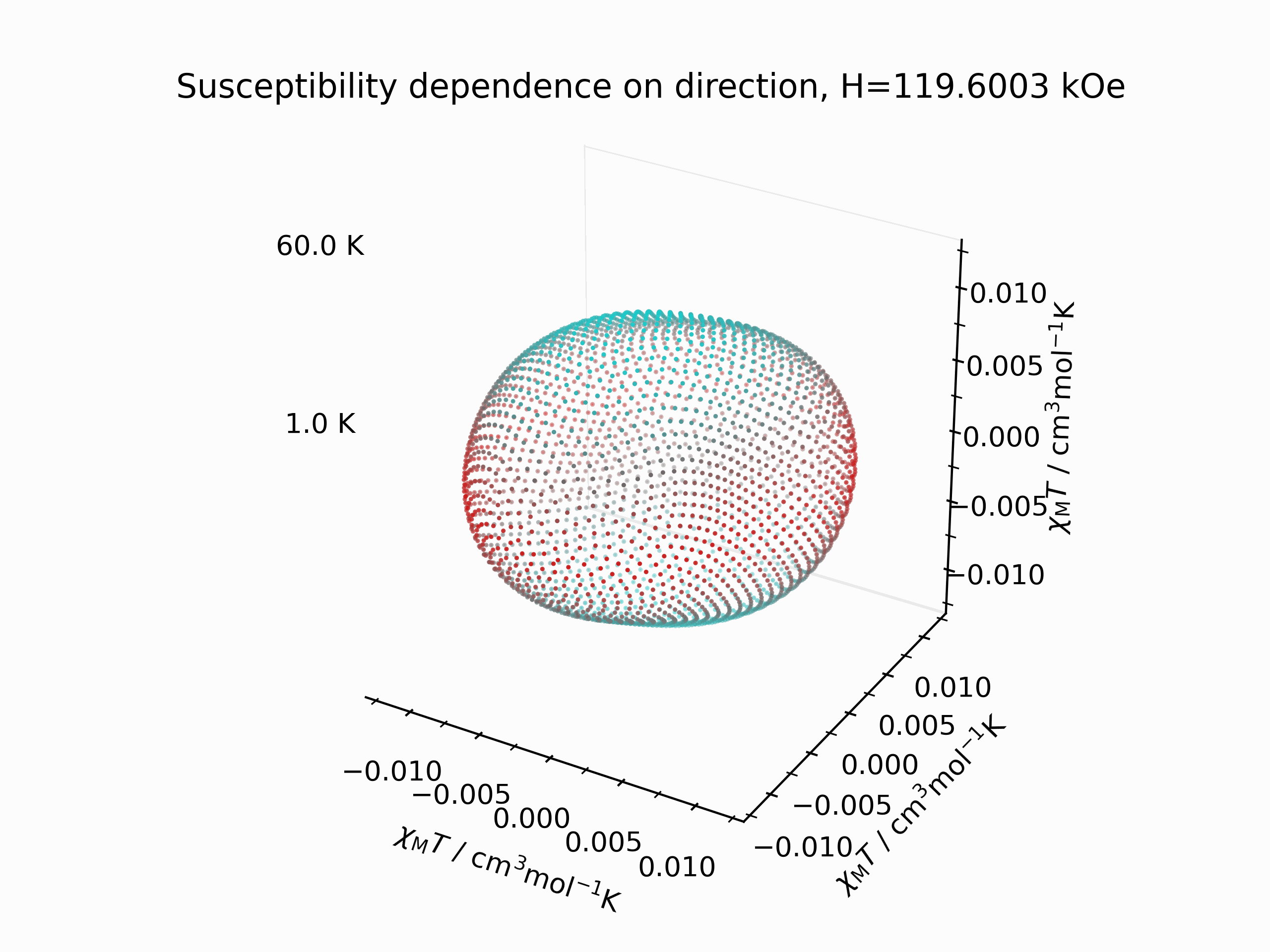

Supplement: Supplementary file 4 — ic4c02793_si_004.zip [file ic4c02793_si_004.zip › Supporting Movie 6.gif]
